# Role of Glutamatergic Projections from Lateral Habenula to Ventral Tegmental Area in Inflammatory Pain-Related Spatial Working Memory Deficits

**DOI:** 10.3390/biomedicines11030820

**Published:** 2023-03-08

**Authors:** Mobina Alemi, Ana Raquel Pereira, Mariana Cerqueira-Nunes, Clara Monteiro, Vasco Galhardo, Helder Cardoso-Cruz

**Affiliations:** 1 Instituto de Investigação e Inovação em Saúde—Pain Neurobiology Group, Universidade do Porto, Rua Alfredo Allen 208, 4200-135 Porto, Portugal; 2 Instituto de Biologia Molecular e Celular, Universidade do Porto, Rua Alfredo Allen 208, 4200-135 Porto, Portugal; 3 Faculdade de Medicina, Departamento de Biomedicina—Unidade de Biologia Experimental, Universidade do Porto, Rua Doutor Plácido da Costa, 4200-450 Porto, Portugal; 4 Programa Doutoral em Neurociências, Faculdade de Medicina, Universidade do Porto, Rua Doutor Plácido da Costa, 4200-450 Porto, Portugal

**Keywords:** lateral habenula, glutamatergic projections, ventral tegmental area, optogenetic inhibition, working memory deficits, inflammatory pain

## Abstract

The lateral habenula (LHb) and the ventral tegmental area (VTA), which form interconnected circuits, have important roles in the crucial control of sensory and cognitive motifs. Signaling in the LHb-VTA pathway can be exacerbated during pain conditions by a hyperactivity of LHb glutamatergic neurons to inhibit local VTA DAergic cells. However, it is still unclear whether and how this circuit is endogenously engaged in pain-related cognitive dysfunctions. To answer this question, we modulated this pathway by expressing halorhodopsin in LHb neurons of adult male rats, and then selectively inhibited the axonal projections from these neurons to the VTA during a working memory (WM) task. Behavioral performance was assessed after the onset of an inflammatory pain model. We evaluated the impact of the inflammatory pain in the VTA synapses by performing immunohistochemical characterization of specific markers for GABAergic (GAD65/67) and dopaminergic neurons (dopamine transporter (DAT), dopamine D2 receptor (D2r) and tyrosine hydroxylase (TH)). Our results revealed that inhibition of LHb terminals in the VTA during the WM delay-period elicits a partial recovery of the performance of pain animals (in higher complexity challenges); this performance was not accompanied by a reduction of nociceptive responses. Finally, we found evidence that the pain-affected animals exhibit VTA structural changes, namely with an upregulation of GAD65/67, and a downregulation of DAT and D2r. These results demonstrate a role of LHb neurons and highlight their responsibility in the stability of the local VTA network, which regulates signaling in frontal areas necessary to support WM processes.

## 1. Introduction

Increasing evidence indicates that pain-related aberrant activity in the lateral habenula (LHb) is associated with depressive symptoms with excessive negative focus leading to high-level cognitive dysfunctions [1,2,3]. In this regard, the LHb appears like a plausible player for working memory (WM) as it receives afferents from the medial prefrontal cortex (mPFC) [4,5], a brain structure also affected during pain states [6,7,8]. Recently, the LHb has been implicated in spatial WM mainly through the cholinergic component [9,10]. Additionally, the LHb is a main input to dopaminergic regions [11], shaping indirectly the dopaminergic drive [12]. This is particularly important as dopamine (DA) is strictly involved in WM encoding [13,14]. In fact, the LHb primary output region is ventral tegmental area (VTA), and both areas share bidirectional connections [15,16,17].

The hypothesis of pain-related cognitive and mood impairments postulates that dysfunctions can result from a deficiency in VTA DA signaling induced by hyperactivity in LHb [3,18,19,20]. The LHb has an inhibitory effect on VTA through its glutamatergic neurons acting on VTA GABAergic neurons, functioning as a control for the VTA DA signaling [11,21]. On the other hand, VTA DA neurons project to several areas through mesocortical and mesolimbic pathways, thus having important roles in cognitive flexibility, motivation and reward-valence processing [22,23,24,25,26]. In addition, LHb also receives diverse related signals originating from dopaminergic structures, which participate in the regulation of positive and negative feedback mechanisms during the cognitive demand [2,16,27]. Thus, functional disruption of the LHb-VTA pathway will affect the regulation of stimuli salience and valence processing, and play a pivotal role in the onset of cognitive alterations that characterize the pathophysiology of chronic pain. 

Here, we hypothesized that LHb-mediated DA suppression, under chronic pain conditions, may interfere with the correct integration and processing of spatial WM information, and that the LHb-dependent facilitation of VTA DA signaling might be needed to improve the normal local network performance by enhancing clusters of neuronal activity that encode reward-valence information during WM encoding. To test this idea, we applied an optogenetic approach to determine whether LHb glutamatergic neurons projecting into the VTA play a role in pain-related WM deficits. We further evaluated the impact of the inflammatory pain in the VTA structural phenotype, and thus, immunohistochemical characterization of the expression of specific markers for GABAergic and dopaminergic neurons was performed.

## 2. Materials and Methods

### 2.1. Rodent Model

Experiments were performed in adult male Sprague-Dawley rats (weight 250–350 g; Charles River Laboratories, Saint Germain, Neulles, France). Before surgery, rats were housed in collective standard cages (type H, 3 per box), containing environmental enrichment, and kept in a 12 h light/dark cycle (lights on at 8:00 a.m.) with constant-controlled temperature (22 ± 2 °C) and humidity (50 ± 5%). Training and recording sessions were performed at approximately the same time each day during the light phase. The testing room was sound attenuated, moderately illuminated, and rich in visual cues. During the course of the experiments, all rats were food deprived to 90–95% of their *ad libitum* feeding, but with unlimited access to water. Rat weights were checked daily after surgery and their growth was monitored weekly compared with a standard curve for Sprague-Dawley rats. Rats were habituated to handling by the experimenters before the start of any experimental procedures. 

### 2.2. Stereotaxic Intracranial Virus Transfection, Optical Fiber and Optrode Implantion

The protocol for the optical fiber and optrode implantation followed the surgical procedures previously described [28]. The following stereotaxic coordinates in millimeters relative to the bregma were used to infuse the viruses into the LHb (3.3–3.5 mm posterior to bregma, 0.7–0.9 mm lateral to midline, depth 4.8 mm), and to implant the optical fiber in the VTA (5.4–6 mm anterior-posterior, 0.9–1.1 medial-lateral, dorsal-ventral 7.7–7.9 mm) [29]. The same coordinates were used to chronically implant the optrode structure for extracellular neuroelectrophysiological recordings. Prior to optical fiber or optrode implantation, an injection of 0.5 µL viral particles (see details below) was delivered in the LHb region at a rate of 0.1 µL/min with a 5 µL microsyringe (Model 7105 KH—tip type 3, Hamilton, Reno, NV, USA). After the infusion, the needle was maintained in place for an additional 15 min period to facilitate the diffusion of the viruses and then slowly withdrawn. Each optrode was composed of two multielectrode bundles, each containing 8 filaments of formvar-coated tungsten wire (35 µm diameter; California Fine Wire Company, Grover Beach, CA, USA) with impedances varying between 0.7 and 1.2 MΩ at 1 kHz. The optical fiber (Plexon Inc., Dallas, TX, USA) was attached to the central portion of the VTA bundle. Each optical fiber cannula was composed of a LC zirconia ferrule (2.5 mm of diameter and 10.5 mm of length), and an optical fiber (0.66 NA) with 200/230 µm of diameter and 12 mm of length. After surgery, analgesic ketoprofen (5 mg/kg) and antibiotic enrofloxacin (5 mg/kg) diluted 1/10 in saline solution were administered subcutaneously every 24 h for 5–7 days. Rats were allowed to recover for 14 days before behavioral testing sessions began, and their overall health status was checked daily.

### 2.3. Experimental Design and Behavioral Procedures

#### 2.3.1. Apparatus and Working Memory Task

**Figure 1:**
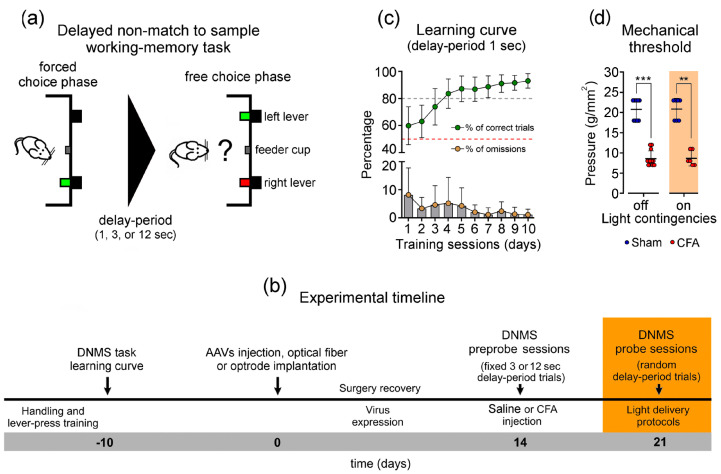
Working memory task, learning curve, and experimental timeline. (**a**) Diagram of delayed non-match to sample (DNMS) WM task used in this study. Each trial began with a single lever being exposed (forced-choice phase). When the rat pressed the lever, it was retracted and the delay-period was initiated. At the end of this period, both levers were exposed (free-choice phase), and to obtain a reward pellet, the rat needed to press the opposite lever selected in the forced-choice phase. (**b**) Timeline of the experimental protocol. In brief, after the learning curve each rat was injected with a virus to express eNpHR3.0 opsin in LHb neurons projecting to VTA, and implanted with an optical fiber in the VTA. After postoperative recovery (14 days), rats were subjected to saline injection (Sham group) or an inflammatory pain model (CFA group) and trained in the DNMS task using six-day single delay-period contingencies (3 or 12 s; ahead referred as pre-probe sessions). Optogenetic neuromodulation protocols were applied 7 days after saline or CFA injection (probe sessions). Light delivery was applied during the delay-period of the DNMS task using a fixed intensity of 5–6 mW at the fiber tip. Each probe session was composed of a random sequence of 3 and 12 s delay-period challenge trials, in equal numbers. Light neuromodulation protocols were applied in independent probe sessions. (**c**) DNMS task learning curve, gain in performance during 10 consecutive training sessions using a delay-period challenge of 1 s (top panel), and corresponding percentage of performed omissions (bottom panel). (**d**) Effects of contralateral photoinhibition of LHb neurons projecting to VTA in mechanical sensitivity threshold. Comparisons between experimental groups and light delivery protocols were based on non-parametric Kruskal-Wallis (KW) test followed by post hoc Dunn’s test. Values are presented as mean ± S. D. Significant results are indicated by ** when *p* < 0.01, and *** when *p* < 0.001.

To evaluate the impact on WM of the selective optogenetic inhibition of LHb-VTA pathway, rats were tested on a classical delayed non-match to sample task (DNMS) as described in our previous report [8,28]. Briefly, testing was conducted in a square arena (45 × 45 cm, long and wide; and 40 cm, high), containing 2 retractable levers and a feeder cup placed between them (Figure 1a). In this WM task, each trial comprised a forced-choice phase in which one of the levers was exposed until the rat pressed the lever (with a time limit of 20 s). After the lever press, a short delay-period was initiated. Behavioral performance was tested using 3 different delay-period challenges: 1 s (only during the learning curve), 3 or 12 s (in the pre-probe sessions) and a double random challenge of 3 and 12 s (in the probe sessions). After the delay-period, free-choice phase started in which both retractable levers were exposed until the rat pressed one (with a time limit of 30 s). A trial was considered correct when the rat pressed the lever that was not presented during the forced-choice phase. Correct trials were rewarded with one pellet. Trials in which the rat failed to make a lever response before the imposed time limit were considered omissions. Trials were separated by an interval of 10 s. A schematic timeline of the experimental design is illustrated in Figure 1b. Behavioral training sessions included different sessions for animals to be habituated and learn how to press levers and receive reward pellets (Figure 1c). After completing this first training stage, learning curve sessions were performed, in which rats did 50 trials/session for 10 consecutive days. Only rats that reached 80% of correct trials in the last 3 sessions of the learning curve were selected to continue the testing protocols. Each rat was then subjected to a stereotaxic surgery for the injection of optogenetic viral vectors, and to the implantation of either an optrode or a sole optical fiber cannula. After postoperative recovery (14 days), each rat was subjected to 6 pre-probe sessions (50 trials/session). Finally, probe sessions with 100 trials/session occurred 21 days after surgery. Probe sessions with or without light delivery were performed separately in the same day. In all cases, each session was structured with an equal number of left and right rewarded trials. The control of pellet feeder and retractable levers was fully automated using the OpenControl software adapted to this task [30]. The reward pellets used throughout the experiments were 45 mg sucrose dustless precision reward pellets (Bioserv—F0023, Frenchtown, NJ, USA). Acclimatization to the testing room was allowed for approximately 1 h. To avoid possible bias, in particular related to the testing group, rats belonging to different experimental groups were tested alternately. The experimenter was blind to group treatment.

#### 2.3.2. Inflammatory Pain Model

A persistent monoarthritis inflammatory pain model [31] was induced by injecting 50 µL of complete Freund’s adjuvant (CFA; 0.5 mg/mL, Sigma Aldrich, cat. No. F5881) under isoflurane anesthesia in the dorsal surface of the rat hindpaw contralateral to the optical fiber or optrode implantation (hereafter referred as CFA group). For control purposes, we applied the same volume of saline solution (NaCl 0.9% *w*/*v*) (hereafter referred as Sham group). Saline or CFA injections were performed 14 days after postoperative recovery. The sensory threshold for noxious stimulation was assessed by placing the rats individually in a circular chamber with a metal mesh floor and touching the plantar surface of the paw with von Frey filaments (Somedic, Sösdala, Sweden) for 8–10 s until slight buckling was caused, as previously described [32]. Rats were acclimated to the testing chamber for 20–30 min before the onset of examination. A traditional Dixon up-down method with von Frey filaments of incremental stiffness (5, 7, 8, 11, 14, 18, 23, 38, 49, 53, 90, 122, 135 g/mm^2^) was used to measure mechanical hypersensitivity. A positive response was considered if the paw was sharply withdrawn or flinching occurred. Each filament was applied 10 times with an interval of 10 s between stimuli. The sensory threshold was considered as a minimum of 5 positive responses. These measurements were performed 1 h after the end of the last behavioral probe session, without and with optogenetic stimulation in independent sessions. In the measurements with light delivery, we used a 10 s continuous pulse per trial (see details about physical stimulation parameters below). The response values are presented in grams per square millimeter.

#### 2.3.3. Optogenetics and Light Delivery

Adeno-associated viruses (AAVs) were obtained from the University of North Carolina Vector Core. Viral titer was 3.0 × 10^12^ particles/mL for AAV5-hSyn-eNpHR3.0-mCherry, and 4.0 × 10^12^ particles/mL for control virus AAV5-CaMKIIα-mCherry (Figure S1 (Supplementary Materials)). Viruses were housed in a −80 °C freezer until the day of infusion (details about injection are reported in Section 2.2 above). Rats expressing inhibitory halorhodopsin (eNpHR3.0 variant) were unilaterally illuminated using an orange LED light (620 nm, PlexonBright, Plexon Inc., Dallas, TX, USA). Prior to optical fiber or optrode implantation, the final light output of the LED source was adjusted to deliver 5–6 mW (irradiance = 158–189.6 mW/mm^2^) of fixed intensity at the end of the optical fiber (light intensity meter, model PM160, ThorLabs, Munich, Germany). Stimulation parameters were controlled with a TTL generator (Prizmatix Pulser 2.0, Holon, Israel) connected to a light LED source (Plexon LD-1 Single Channel LED-Driver; Plexon Inc., Dallas, TX, USA). Light was delivered to VTA in a focal area encompassing the recording multielectrode structure. The connection between the patch cable and the implanted optical fiber was completely covered with black adhesive tape to prevent light scattering from becoming a cue to the rats. Behavioral probe sessions were performed 21 days after viral injection to enable full viral expression. Optogenetic photoinhibition was applied during the delay-period of the DNMS task.

#### 2.3.4. Neuroelectrophysiological Recordings

Extracellular single-unit and local field potentials (LFPs) recordings were obtained from an implanted intracranial multielectrode array structure. The 16-channel multielectrode array was connected to a wireless high-frequency headstage transmitter (W16; Triangle Biosystems, Durham, NC, USA) that sent continuous analog signals to a Multineuron Acquisition Processor system (16MAP, Plexon Inc., Dallas, TX, USA). Neural signals were preamplified (10,000–20,000X) and digitized at 40 kHz. Voltage-time threshold windows were used to identify single-unit waveforms online (SortClient 2.6; Plexon) and validated offline using automatic and manual sorting techniques (Offline Sorter 2.8; Plexon Inc., Dallas, TX, USA) according to the cumulative criteria described in detail previously [33]. Extracellular LFFs signals were obtained by low-frequency (0.2–200 Hz) filtering of the raw signals. LFPs were preamplified and digitized at a 0.5 kHz sampling rate. These data were subsequently processed offline using the NeuroExplorer 4 software (NEX 4, Plexon Inc., Dallas, TX, USA) and exported to MatLab (R17, MathWorks, Natick, MA, USA) to be further analyzed using custom routines.

#### 2.3.5. Histological Validation

After the end of last recording sessions, rats were deeply anesthetized with pentobarbital sodium (150 mg/kg, i. p.) and transcardially perfused with 0.01 M phosphate buffer, pH = 7.2, in a 0.9% (*w*/*v*) saline solution, followed by 4% paraformaldehyde. Brains were removed, post fixed in 4% paraformaldehyde for 4 h and stored in 30% (*w*/*v*) sucrose solution before they were frozen, and later sectioned into 30 µm slices. Brain slices were used to locate optrode or optical fiber end position, and to quantify opsins expression in the tissue. The final location coordinates were identified using a rat brain atlas [29]. Stained sections were observed using a Zeiss Z1 Apotome microscope. Only rats with correct location of the implanted optrode and optical fiber, as well as sufficient viral expression were included in the data analysis.

#### 2.3.6. Immunohistochemical Characterization of the VTA Structural Phenotype

To further assess the effect of CFA treatment on VTA cells, and to observe the LHb terminals in VTA and their contact with inhibitory/excitatory cells, we injected a second set of animals with the AAV5-hSyn-eNpHR3.0-mCherry virus and after one week their hindpaw with saline or CFA (see details before). Finally, one week after the peripheral injection, the rats were sacrificed and their brains were post fixed and sectioned into 30 µm slices as described before. Sections were washed for 30 min in phosphate-buffered saline 0.1 M (PBS) and blocked with 10% normal goat serum (Biowest, Nuaille, France) in PBS-Triton (PBS-T) for 2 h. After washing with PBS-T, they were incubated two overnights with antibody against GAD67 (Cat. No. MAB5406; Merck Millipore, Algés, Portugal) or one overnight with antibodies against GAD65/67 (Cat. No. PA1-84572, Thermofisher Invitrogen, Carcavelos, Portugal), tyrosine hydroxylase (TH; Cat. No. MAB318, Merck Millipore), DA D2 receptor (D2r; Cat. No. MABN53, Merck Millipore) or DA transporter (DAT; Cat. No. MA5-24796, Thermofisher Invitrogen), respectively diluted 1:1000, 1:600, 1:500, 1:400 and 1:300 in 2% normal goat serum in PBS-T. Then sections were washed with PBS-T and incubated for 1 h with Alexa-488 goat anti-mouse (Cat. No. A-32723; Thermofisher Invitrogen), Alexa-568 goat anti-mouse (Cat. No. A-11004; Thermofisher Invitrogen), Alexa-488 goat anti-rabbit (Cat. No. A-11034; Thermofisher Invitrogen) or Alexa-647 goat anti-rabbit (Cat. No. A-32733; Thermofisher Invitrogen) antibodies, respectively diluted in 1:1000, 1:800 or 1:500 in PBS-T. Finally, sections were washed well with PBS, and cell nuclei were stained with DAPI (2 mg/mL in glycerol-mounting media). Stained sections were observed using a Zeiss Z1 Apotome microscope. For both experimental groups, all images of each marker were acquired in the same session with the same physical exposure parameters. Average fluorescence intensity per area in the images was quantified using Fiji software version 1.53c [34,35] (Section 3.2.).

### 2.4. Data Analysis and Representation

While data used in this study came from well-trained rats, we identified the probe sessions that had at least 5% incorrect trials so that further analysis could be made to determine the response latency to the free-choice lever press during incorrect trials. To validate the functional effects of light delivery on recorded units, we compared the background activity during a 6 s light-on versus light-off baseline period for each neuron. To further characterize light-dependent alterations in LFP signals, we analyzed the power spectral density (PSD) of LHb and VTA recorded channels. The PSD of the LFP signals were computed using Fast Fourier Transform analysis (FFT, 512-point), and the P. Welch method as the spectral estimator (MatLab native function), over the 0–50 Hz range of frequencies (0.19 Hz of resolution). Five LFPs frequency bands were considered: 1–4 Hz (delta, *δ*), 4–9 Hz (theta, *θ*), 9–15 Hz (alpha, *α*), 15–30 Hz (beta, *β*), and 30–50 (slow-gamma, *γ*). Then, we performed a two-sample Kolmogorov-Smirnov test (ks2-test, *p* < 0.05) to identify differences on firing and power distributions. For behavioral experiments, subject numbers were determined by pilot studies and power analysis (power = 0.80, significance level = 0.05, effect size = 15–30%). The Kolmogorov-Smirnov (*KS*) test (with Dallal-Wilkinson-Lilliefor corrected *p*-value) was used to determine whether datasets were normally distributed (GraphPad Prism 8.0, San Diego, CA, USA). For single comparisons, we used the parametric *t*-test for unpaired samples (with Welch’s correction) or non-parametric Mann-Whitney test. For multiple comparisons, we used the non-parametric Kruskal-Wallis analysis of ranks (KW) (with post hoc Dunn’s test). All effects presented as statistically significant exceeded an α-threshold of 0.05. All independence tests were two-tailed. Data in the text are presented as mean ± S. D. Custom Python (version 3.8) and MatLab routines were used to classify behavioral data. Details about key resources used in this study were included in Table 1.

## 3. Results

### 3.1. Working Memory Task and Learning Curve

In this study, we used a classical DNMS WM task to test the impact of an inflammatory pain condition in the LHb neurons projecting to VTA during the cognitive demand (Figure 1a). The experimental timeline for behavioral testing is illustrated in Figure 1b. After the habituation and handling period, rats were trained using a single delay-period challenge of 1 s (learning curve phase). Rats progressed through the training phases at different rates. From an initial group of 38 rats, 37 rats reached the inclusion criterion within 10 daily training sessions (Figure 1c, top panel). This performance progress was also accompanied by a reduction in the percentage of trial omissions (Figure 1c, bottom panel). After the learning curve, seven rats were excluded due to surgical complications, and nine rats were excluded by incorrect optical fiber position or viral expression. 

### 3.2. Impact of Inhibition of LHb Glutamatergic Terminals into the VTA on Inflammatory Pain

To determine the role of LHb eNpHR3.0-expressing neurons projecting to the VTA in nociceptive responses, we assessed mechanical withdrawal thresholds using von Frey test in both Sham and CFA rats. The optogenetic photoinhibition of LHb glutamatergic terminals within the VTA did not reduce nociceptive responses in CFA-treated rats (7 days after induction; KW = 26.39, *p* < 0.0001; Sham vs. CFA, light-off: *p* < 0.001, and light-on: *p* < 0.01, post hoc Dunn’s test; Figure 1d).

### 3.3. Specific Optogenetic Inhibition Strategy of LHb Glutamatergic Neurons Terminals into the VTA

**Figure 2:**
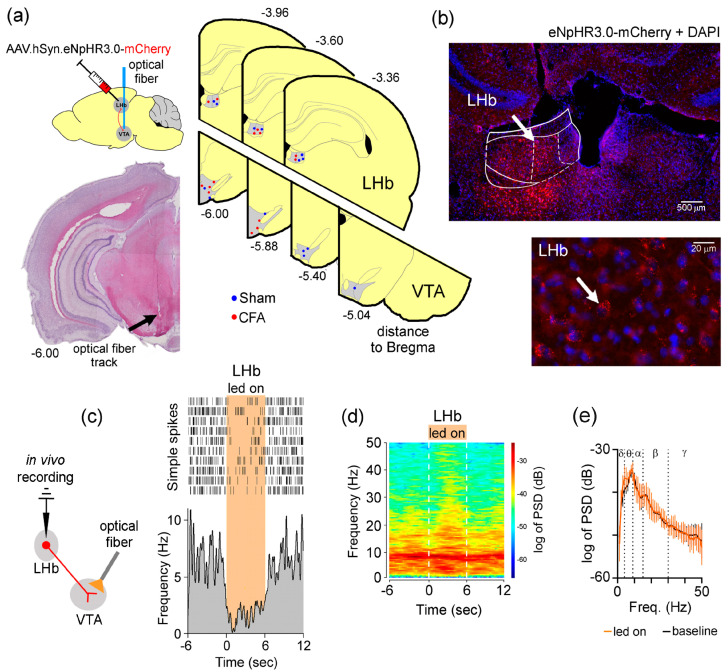
Optical fiber location, virus expression in LHb neuron projecting to VTA, and functional electrophysiological validation of the selective optogenetic neuromodulation protocol. (**a**) Top left panel illustrates the experimental paradigm for optogenetic viral particles infusion in the LHb and optical fiber implantation in the VTA, and bottom left panel represents an example of a histological brain section consisting the optical fiber tip location in the VTA. Right coronal diagrams illustrating the virus infusion locations in the LHb (top panel) and the optical fiber locations in the VTA (right bottom panels). Top and bottom numbers represent the anterior-posterior distance (in millimeters) relative to bregma cranial point. Sham-treated rats illustrated by blue dots, and CFA-treated rats by red dots. (**b**) Coronal slice example of the LHb transfected area (top panel), and magnification of the LHb area (bottom panel). Red fluorescent protein (mCherry) labeling indicates eNpHR3.0 LHb transfected neurons. Blue dots represent the DAPI, DNA-labeling as nuclear counterstain. (**c**) Strategy used for optogenetic inhibition of LHb glutamatergic neuron terminals into the VTA (left panel). Effects of optogenetic photoinhibition in the background activity of a LHb eNpHR3.0-expressing unit. Vertical orange rectangle indicates the period of light photoinhibition. The recorded unit exhibited significant inhibition of their discharge activity during the light delivery period. (**d**) Full spectrogram of raw LFP activity recorded in the LHb, and (**e**) the corresponding power spectral density (PSD) traces representing 6 s of ongoing LFP activity recorded during the photoinhibition period, delimited by vertical dotted lines. Power distribution traces were compared using a two-side Kolmogorov-Smirnov test (ks2-test, *p* < 0.05). LFP signals power traces indicate a light-dependent effect (LHb: ks2 = 0.38, *p* = 0.0009). Frequency bands: *δ*, 1–4 Hz; *θ*, 4–9 Hz; *α*, 9–15 Hz; *β*, 15–30 Hz; *γ*, 30–50 Hz. Physical parameters of photoinhibition: continuous orange pulse at 620 nm, and intensity of 5–6 mW. Values are presented as mean ± S. D.

To achieve the selective photoinhibition of LHb neurons projecting to VTA, we injected in the LHb an adeno-associated virus (AAV) encoding eNpHR3.0 infused in-frame to mCherry under control of the hSyn-promoter and implanted the optical fiber in the VTA. An example of the histological confirmation of optical fiber track in the VTA and a schematic illustration of the locations of virus infusion in the LHb are presented in Figure 2a. Stereotaxic delivery of AAV5-hSyn-eNpHR3.0-mCherry virus resulted in transfection in the LHb cells (Figure 2b). To test the efficiency of viral transfection and light delivery protocol in LHb glutamatergic neurons projecting into the VTA, we recorded the neural activity in the LHb. To illustrate the light-dependent effects on neural firing distributions, an example of significant inhibition of activity of a LHb recorded neuron is given in Figure 2c. In this case, we compared the neural activity during a 6-s light-on versus light-off period (baseline). The recorded unit exhibited a clear significant firing activity suppression during the stimulation period. These changes were also accompanied by important alterations on local LFP signals power oscillations during the time-window of stimulation (Figure 2d). In this regard, we found different activity distributions when comparing the LFP signals power traces in respect to baseline indicating a light-dependent effect on both recorded brain areas (LHb: *ks2* = 0.38, *p* = 0.0009; Figure 2e). This was particularly evident across theta frequency-band (*θ*, 4–9 Hz). 

**Figure 3:**
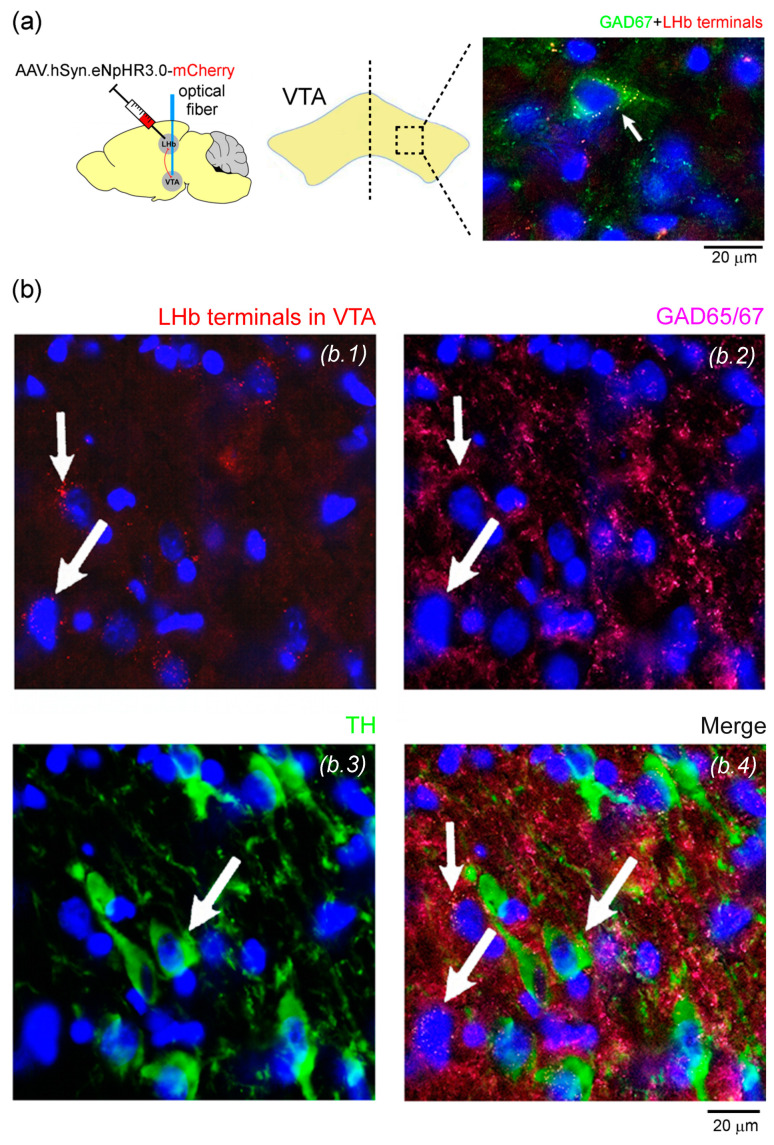
Tissue validation of LHb eNpHR3.0-expressing neurons targeting into the VTA. Immunofluorescent images showing eNpHR3.0-expressing axonal terminals of LHb neurons targeting into the VTA GABAergic cells. (**a**) Colocalization of LHb terminals with VTA GAD67-positive neurons; the arrow indicates a green inhibitory GABAergic neuron co-labeled with a mCherry LHb terminal in the VTA. DAPI labeling (blue) was used to nuclear counterstain. (**b**) From the left to the right, LHb terminals in VTA (**b.1**; mCherry, red), VTA GABAergic inhibitory (**b.2**; GAD65/67-marker)-positive neurons (pink), VTA tyrosine hydroxylase (TH)-positive neurons (**b.3**; green), and finally the respective merged image of all channels (**b.4**). The arrows show some examples of colocalization of LHb terminals with VTA inhibitory neurons. Scale bars, 20 µm. All sections are taken 5.40–6.00 mm posterior to the Bregma. Supplementary material are provided in Source Data S1.

Immunohistochemical analysis confirmed the expression of eNpHR3.0-mCherry at the projection terminals of the glutamatergic neurons from the LHb around the neuronal cell bodies in VTA GABAergic neurons (GAD65/67 positive neurons), and showed that only a residual number of TH-positive neurons co-localized with LHb terminals in VTA (Figure 3).

### 3.4. The Impact of Selective Photoinhibition of LHb Glutamatergic Terminals into the VTA in Inflammatory Pain-Related Working Memory Performance

**Figure 4:**
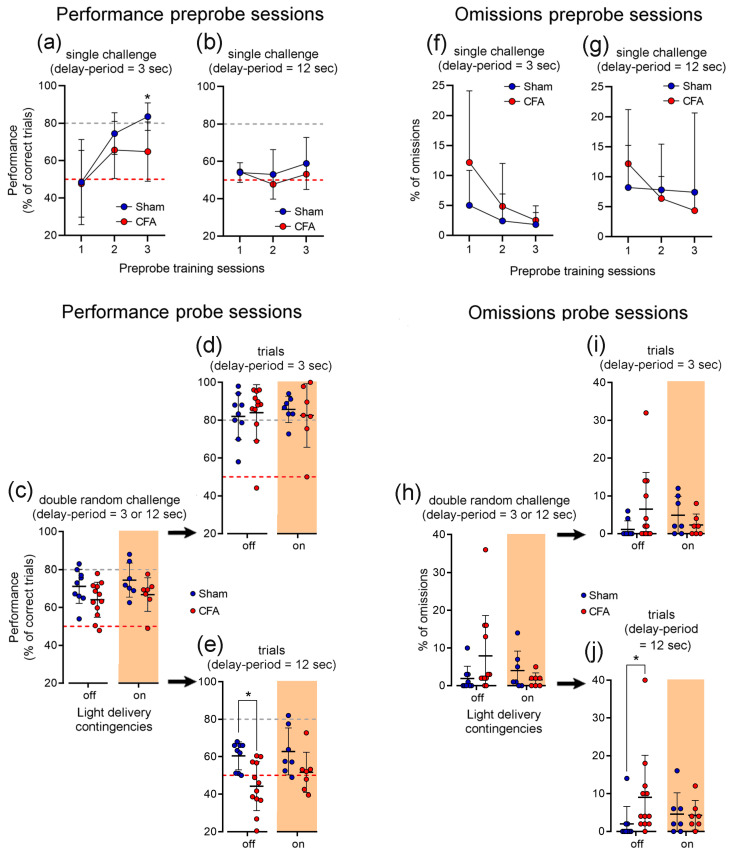
Effects of optogenetic photoinhibition of LHb neuron axon terminals into the VTA on working memory performance. Behavioral performance of DNMS task pre-probe training sessions using a single delay-period challenge per session: (**a**) 3, and (**b**) 12 s. (**c**) Behavioral performance of DNMS task probe sessions with optical neuromodulation of LHb glutamatergic neuron terminals into the VTA using a double random challenge per session. Averaged performance in trials with 3 s (**d**), and 12 s (**e**) of delay-period. Percentage of omissions performed in pre-probe training sessions using a single delay-period challenge per session: (**f**) 3, and (**g**) 12 s. (**h**) Percentage of omissions performed in probe sessions with optical neuromodulation of LHb glutamatergic neuron terminals into the VTA using a double random challenge per session. Averaged percentage of omissions across the trials with 3 s (**i**), and 12 s (**j**) of delay-period. Physical parameters of photoinhibition: continuous orange solid pulse at 620 nm, and intensity of 5–6 mW. Comparisons between experimental groups and light delivery protocols are based on non-parametric Kruskal-Wallis (KW) test followed by post hoc Dunn’s test. Values are presented as mean ± S. D. Significant results are indicated by * when *p* < 0.05.

After the postoperative recovery, each rat was subjected to the CFA or saline injection and tested in six daily random pre-probe sessions each composed of a single delay-period challenge (3 or 12 s) (Figure 4). In respect to pre-probe sessions, our data revealed a significant effect between experimental groups during the 3 s delay-period challenge sessions (KW = 27.00, *p* < 0.0001; Figure 4a); moreover, post hoc test revealed that sham-treated rats in the last session exhibited a higher performance when compared to CFA-treated rats (day/session number 3: *p* < 0.05; Dunn’s test). For 12 s delay-period challenge pre-probe sessions, no significant differences were observed between experimental groups (KW = 5.96, *p* = 0.3106; Figure 4b). Seven days after the CFA or saline injection, each rat was tested in two independent probe sessions, with and without optogenetic photoinhibition of LHb projecting neuron terminals into the VTA during the delay-period of the DNMS task (Figure 4c–e). Each probe session was composed of random trials with different delay-period complexities (3 or 12 s) in equal number (Figure 4c). Our data revealed no significant statistical effect between the experimental groups and optogenetic protocols (KW = 5.19, *p* = 0.1585). Next, from these sessions we repeated the analysis considering only the trials that correspond to each delay-period contingency (Figure 4d,e). For 3 s delay-period trials, our data showed no significant effect between groups and treatments (KW = 0.57, *p* = 0.9037; Figure 4d); however, for 12 s delay-period trials, a significant effect was observed between groups and treatments (KW = 11.66, *p* = 0.0087; Figure 4e). Moreover, sham-treated rats revealed a higher performance level without light photoinhibition when compared to CFA-treated rats (light-off: sham vs. CFA, *p* < 0.05). Interestingly, when both experimental groups were subjected to photoinhibition treatment, they exhibited a similar performance, indicating the effect of LHb inhibition in their partial recovery. 

To further analyze the impact of trial omissions on the WM performance of each experimental group, we calculated the percentage of omissions per behavioral contingency (Figure 4f,g). In respect to pre-probe sessions, we found that CFA-treated rats exhibited a higher percentage of omissions when compared to controls using a delay-period challenge of 3 s (KW = 14.41, *p* = 0.0132; Figure 4f), but this effect was not extended to 12 s sessions (KW = 7.00, *p* = 0.2210; Figure 4g). In the case of the probe sessions composed of double random challenges (Figure 4h), no significant differences were observed between groups and optogenetic treatments (KW = 4.72, *p* = 0.1933). Next, from these sessions we took into consideration only the trials that corresponded to each contingency (Figure 4i,j). For 3 s challenge, no significant effect between treatments was observed (KW = 4.26, *p* = 0.2346; Figure 4i), in contrast to the 12 s challenge (KW = 7.81, *p* = 0.050; Figure 4j). Moreover, for 12 s without light inhibition the post hoc test revealed that CFA-treated rats performed more omissions when compared to controls (light-off: sham vs. CFA, *p* < 0.05; post hoc Dunn’s test).

### 3.5. Effect of Inflammatory Pain on Lever Press Response Latency

**Figure 5:**
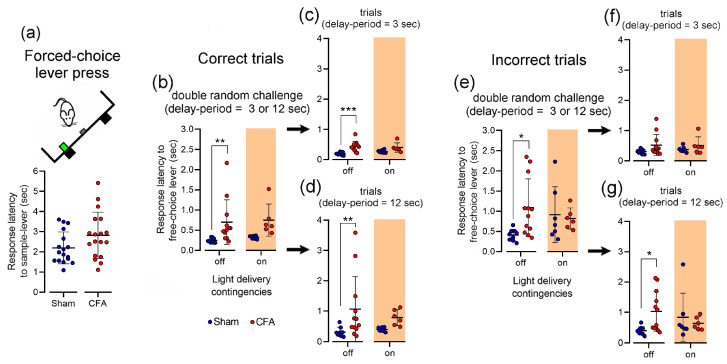
Effects of optogenetic photoinhibition of LHb neuron axon terminals into the VTA on working memory task response latency. (**a**) Response latency to forced-choice phase lever press. (**b**) Response latency to free-choice phase lever press during correct trials in probe sessions using a double random challenge. Averaged latency response during 3 s (**c**), and 12 s (**d**) delay-period complexity trials. (**e**) Response latency to free-choice phase lever press during incorrect trials in probe sessions using a double random challenge. Averaged latency response during 3 s (**f**), and 12 s (**g**) trials. Physical parameters of photoinhibition: continuous solid orange pulse at 620 nm, and intensity of 5–6 mW. Comparisons between experimental groups and light delivery protocols are based on unpaired t-test or Kruskal-Wallis (KW) test followed by post hoc Dunn’s test. Values are presented as mean ± S. D. Significant results are indicated by * when *p* < 0.05, ** when *p* < 0.01, and *** when *p* < 0.001.

To further examine the relationship between response latency and WM behavioral performance, we calculated the average lever press response latency to forced-choice or free-choice phases in both correct and incorrect trials (Figure 5). In respect to forced-choice phase lever, our data revealed no significant differences between experimental groups (unpaired *t*-test; *p* = 0.0715, *t* = 1.862, *df* = 33; Figure 5a). In the correct trials of free-choice phase, statistical analyses revealed a significant effect between the experimental groups and light delivery protocols (KW = 19.65, *p* = 0.0002; Figure 5b); post hoc testing further revealed that CFA-treated rats exhibit a higher response latency when compared to controls in the absence of photoinhibition during the delay-period (post hoc Dunn’s test, *p* < 0.01). Next, from these sessions we repeated the analysis considering only the trials that correspond to each delay-period contingency. For both delay-period challenges, statistical analysis revealed a significant effect between groups and treatments (3 s: KW = 18.32, *p* = 0.0004; and 12 s: KW = 15.12, *p* = 0.0017; Figure 5c,d, respectively); post hoc testing revealed also that CFA-treated rats exhibit a higher response latency when compared to controls (light-off: 3 s, *p* < 0.001; and 12 s, *p* < 0.01). Interestingly, after photoinhibition, these response latencies in CFA animals were again similar to controls. In the case of incorrect trials, statistical analysis revealed a significant effect between groups and light delivery protocols (KW = 10.46, *p* = 0.0151; Figure 5e); moreover, post hoc testing revealed that CFA-treated rats exhibit a higher response latency when compared to controls (light-off: sham vs. CFA, *p* < 0.05). It is important to note that when only the response latency pattern associated to 3 s delay-period of the incorrect trials was analyzed, no significant differences were observed (KW = 4.67, *p* = 0.1976; Figure 5f). In contrast, during 12 s delay-period of the incorrect trials, we found a significant effect between experimental groups and light delivery protocols (KW = 8.50, *p* = 0.0368; Figure 5g). In fact, post hoc test also revealed a higher response latency in CFA-treated rats (light-off: sham vs. CFA, *p* < 0.05). Further, experimental groups subjected to photoinhibition again exhibited a similar latency, indicating the effect of LHb inhibition in their partial recovery in the performance.

### 3.6. Pain-Related VTA Structural Phenotype Alterations

**Figure 6:**
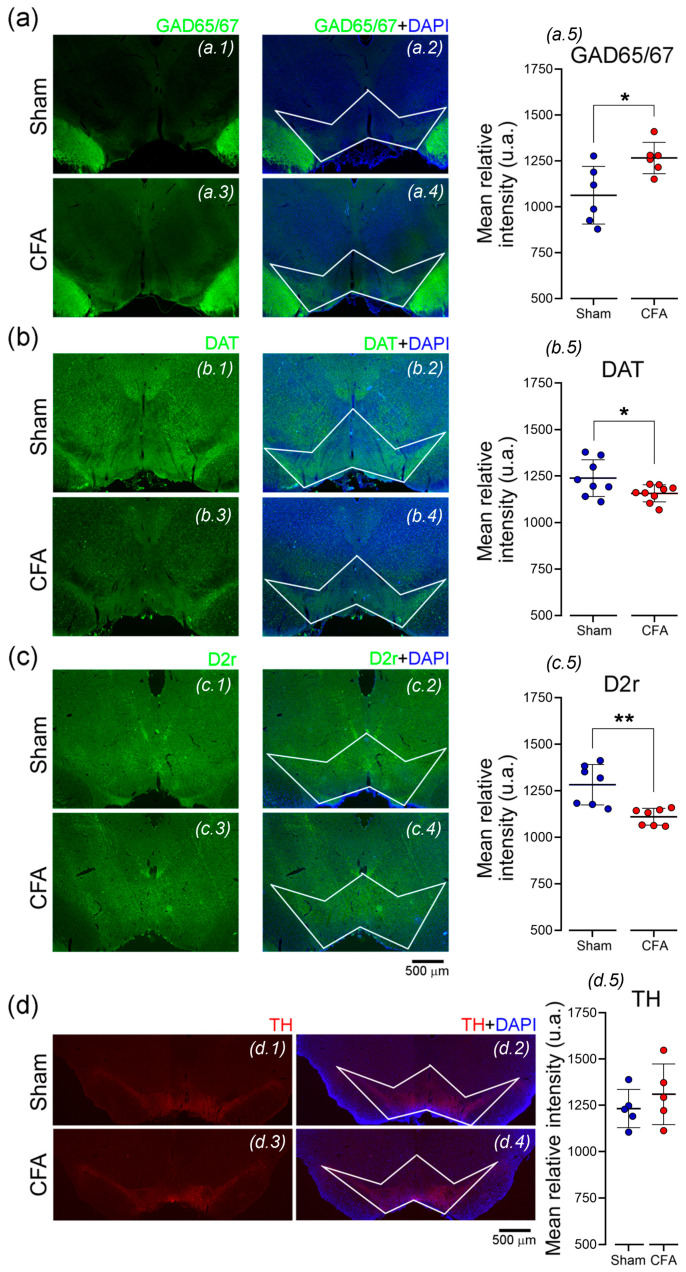
Pain induced local VTA cell-type phenotype alterations. Representative 2.5× images showing an upregulation in (**a**) GAD65/67 marker of GABAergic inhibitory neurons, and a downregulation in (**b**) dopamine transporter (DAT) and (**c**) dopamine D2 receptors (D2r) in the VTA of Sham- and CFA-treated rats. While for (**d**) tyrosine hydroxylase (TH, red), no significant change was observed between experimental groups. Illustrative immunofluorescent images of Sham- (subpanels: **a.1**, **b.1**, **c.1**, and **d.1**; respectively) and CFA-treated rats (subpanels: **a.3**, **b.3**, **c.3**, and **d.3**; respectively). DAPI labeling (blue) was used to nuclear counterstain (Sham group subpanels: **a.2**, **b.2**, **c.2**, and **d.2**; and CFA group subpanels: **a.4**, **b.4**, **c.4**, and **d.4**; respectively). Scale bars, 500 µm. All sections are taken 5.80–6.00 mm posterior to the bregma. White polygon indicates the coronal slice area used to measure the mean relative fluorescence intensity. Comparisons between experimental groups are based on parametric unpaired t-test or non-parametric Mann-Whitney (MW) test. n = 5–9 per group (right panels). Values are presented as mean ± S. D. Significant results are indicated by * when *p* < 0.05 and by ** when *p* < 0.01. Supplementary material are provided in Source Data S2.

To determine if inflammatory pain caused alterations in synaptic transmission, we quantified the expression of GABA or DA cell markers in the VTA of sham or CFA rats. We calculated the mean relative fluorescence intensity in the VTA delimited area. Our data showed that CFA-treated rats reveal an upregulation in GAD65/67 marker when compared with controls, suggesting an overactivation in VTA inhibitory GABAergic neurons (*MW*, *U* = 4, *p* = 0.0260; Figure 6a). In contrast, a downregulation in DAT (unpaired *t*-test, *p* = 0.0403 (*t* = 2.245, *df* = 15); Figure 6b) and D2r (*MW*, *U* = 1, *p* = 0.0012; Figure 6c) was observed. Interestingly, no significant differences were observed in TH expression (unpaired *t*-test, *p* = 0.4025 (*t* = 0.89, *df* = 6.75); Figure 6d).

## 4. Discussion

There is still limited understanding on how WM cortical information is relayed subcortically. By its connectivity, the LHb acts as a subcortical relay for cortical information, sharing direct and indirect inputs to several brain areas [36,37]. However, little is known about the role of LHb excitatory drive to the VTA during pain-related cognitive demand. Here, we examined the impact of selective inhibition of the LHb glutamatergic neurons projecting to the VTA during execution of a WM task, in the presence or absence of an inflammatory pain condition. The major findings of this study include the following: (1) while pain-affected animals show decreased performance in the WM task, (2) selective photoinhibition of the LHb terminals in the VTA during the higher complexity delay-periods elicited a partial recovery of the WM deficits in pain animals (3) but had no significant impact on peripheral pain thresholds. Finally, (4) we demonstrated that inflammatory pain underlies alterations in the VTA local structural phenotype.

### 4.1. Role of LHb Glutamatergic Neurons Projecting into the VTA during Cognitive Demand

It is widely explored by preclinical and clinical reports that chronic pain can lead to a significant reorganization of central areas strictly linked to complex cognitive functions [38,39,40]. The LHb is well-known to play a prominent role in the coding of negative motivational responses [41], particularly in response to stressful conditions such as chronic pain [3]. The LHb participates in behavioral and physiological responses to pain-related depression [42], and chemogenetic inhibition of LHb neurons [43] or LHb lesion reduces peripheral pain threshold [19]. The LHb also responds to stressful [44] and aversive stimuli [45], and is thought to influence mPFC-related WM encoding processes. In fact, the deficits resulting from LHb lesion or transient inactivation confirm its indirect involvement in WM encoding [46,47]. Using a DNMS spatial WM task, we found evidence that CFA-treated rats exhibited a lower WM performance when compared to controls during the pre-probe sessions with lower complexity (Figure 4a), whereas during higher complexity challenges both experimental groups exhibited a chance level performance. It is important to note that this effect was later abolished during probe sessions (day seven post-CFA injection), suggesting that probably this effect may be linked to an impairment of motor capability which reduces the animal behavior accuracy during early pain state. In line with this observation, we have also reported previously that CFA-treated rats revealed a clear reduction of their motor activity during early pain state, interfering with their ability to perform of a classical food-reinforced spatial alternation task [39]. Under the optogenetic protocol, we found no evidence of differences in the performance level between the experimental groups in the probe sessions composed of a random sequence of trials with lower/high complexity (Figure 4c). However, if we consider only the higher complexity trials (12 s), CFA-treated rats exhibited a lower WM performance and higher percentage of omissions when compared to controls, which was reversed during the trials performed with photoinhibition (Figure 4d,e). It is important to mention that, in this case, the optical inhibition was also able to reverse the differences obtained between the experimental groups suggesting that the modulation of LHb glutamatergic projections to the VTA may have a greater impact on more complex challenges. This response was also accompanied by a significant reduction in trial omissions suggesting that the optical inhibition may also have a positive impact on motivation control of pain rats in the operant task. Another important behavioral parameter is the latency of response to lever selection, which is strictly linked to the level of impulsivity during the decision-making process. As expected, we found evidence that CFA-treated rats showed higher latency responses (Figure 5b,e), but the differences were attenuated by the photoinhibition protocol. Chronic pain has been repeatedly associated to a reduction of motivation and motor activity [48,49]. This occurs through their impact in dopaminergic centers, causing a lack of motivation to perform goal-direct tasks [50,51,52]. Here, we thus hypothesized that the selective inhibition of LHb glutamatergic neurons would reduce local VTA GABAergic inhibitory drive over DA neurons, and therefore control hedonic behavior and potentiate WM performance in pain animals. In line with this hypothesis, Markovic and colleagues (2021) have elegantly shown that activation of VTA DA and/or VTA to nucleus accumbens shell area pathway is sufficient to restore baseline motivation and hedonic responses to natural rewards in CFA-treated rodents, suggesting that pain-related adaptations within VTA DA neurons may drive anhedonia-like behaviors. Additionally, they found also that decreased VTA DA neurons activity is in part due to an increased inhibitory tone from the rostromedial tegmental area (RMTg). In the opposite direction, it has been shown that decreased dopaminergic activity in the VTA reduces motivated behavioral responses in challenging tasks [53]. Collectively, these data suggest that the motivational and reward encoding components may have an important impact on behavior accuracy, particularly in food-reinforced WM-dependent tasks during chronic pain conditions.

### 4.2. Role of LHb Glutamatergic Neurons Projecting into the VTA in Pain-Related Responses

A special note should be given to the LHb role in pain perception and processing. It is widely supported by previous reports showing that chronic pain conditions can lead to a reorganization and an overactivation of the local LHb networks [3,19]. In rodents, electrophysiological studies have reported that the majority of LHb neurons increase their activity in response to peripheral noxious stimuli [19,54,55]. Similarly, clinical imaging reports have shown a clear activation of the LHb during acute and chronic pain [3,56]. On the other hand, several studies have found that manipulation of the LHb activity results in anti-nociception [18,43,57], indicating the involvement of the LHb in pain modulation. For example, local morphine injection or electrical stimulation in the LHb relieves acute inflammatory pain caused by formalin injection [57]. It has also been described that selective inhibition of the LHb glutamatergic neurons has been linked to a reduction in primary hypersensitivity, widespread allodynia [58], and hyperalgesia that usually accompanies the withdrawal phase of alcohol consumption [43]. Recently, an increased activity of the LHb glutamatergic neurons has also been reported after infraorbital nerve injury and chemogenetic inhibition of these neurons alleviated anxiety, but not allodynia [59]. In addition, the LHb pain-activation has also been revealed to receive diverse related signals originating from dopaminergic structures participating in the regulation of positive and negative feedback mechanisms [27]. In this regard, the VTA electrical stimulation produces analgesia and reduces thermal and mechanical responses on carrageenan-induced hyperalgesia model [18]. Collectively with our results, we found no evidence that inhibition of LHb glutamatergic neurons contributes to altering the peripheral inflammatory pain threshold. The lack of a direct impact on nociceptive responses may be explained by the indirect impact exerted by other parallel pathways over the VTA, which were not selectively affected by optogenetic manipulation. It has been shown that from LHb to VTA the main indirect pathway is through the RMTg. LHb glutamatergic projections form synapses on local RMTg GABAergic neurons exerting an inhibitory tone into the VTA DA neurons [15]. In line with this observation, it has been shown that enhancing VTA DA activity is not enough to relieve CFA-induced hyperalgesia, suggesting that modulation of afferent nociceptive information through VTA DA neurotransmission may have a more preponderant role in emotional components of inflammatory pain [60].

### 4.3. The Impact of Inflammatory Pain on Local VTA Cell-Type Phenotype 

Given the involvement of VTA in analgesia, it is not surprising that abnormalities of VTA structural phenotype or DAergic transmission can contribute to pain conditions [61]. In fact, here we found an upregulation in the protein marker GAD65/67 of VTA GABAergic neurons, and a downregulation of DAT and D2r expression in the CFA-treated rats (Figure 6). These findings support the hypothesis that inflammatory pain could have triggered a network suppression effect generated by an abnormal VTA GABAergic activity, which in part can lead to a decreased DAergic activity typically associated with pathological chronic pain conditions [62,63]. Since the synthesis of DA is limited by the TH enzyme, it would be expected also a theoretical downregulation of their signal. However, these changes were not accompanied by differences in the TH enzyme immunocytochemical signal. Recently, it has been reported that not all VTA DA cells activity is affected by chronic pain, the lateral and medial VTA DA neurons seems to be differentially affected during pain conditions [52,61]. This highlights the necessity of future studies that could explore the importance of VTA subpopulations specificity when targeting the effects of inflammatory pain.

## 5. Conclusions

In summary, the present study provides new insights on how the LHb glutamatergic neurons projecting to the VTA play an important role in the complex neural activity balance necessary to sustain the habenular network inhibitory/excitatory stability and the input selectivity for the WM information processing during the cognitive demand. Together, our findings suggest that restoring the balance of LHb local network activity may be a strategy to reverse cognitive deficits observed in inflammatory pain conditions.

However, our study had several limitations. We found that inhibition of LHb terminals into the VTA did not induce any significant impact on pain responses, but this could be related to the low intensity of optogenetic protocols applied. This is a complex limitation because of the intrinsic difficulty in achieving pain inhibitory protocols that do not interfere in the cognitive dimension. In this regard, further studies remain necessary to clarify the role of indirect pathways that may compete to exert a direct inhibitory effect over the VTA DA cells. Finally, another limitation of the present study can be associated with a differential gender effect of inflammatory pain. It is important to note that pain also exhibits important sex dimorphism, including in LHb-dependent functions. Recent data showed that sex-specific differences are apparent on local LHb and VTA areas [56,64,65]. Whether these sex-specific differences in LHb-VTA microcircuit function are relevant for inflammatory pain phenotype or not, this will constitute an intriguing field of research in the future. 

## Figures and Tables

**Table 1:** Key resources table.

Reagent or Resource	Source	Identifier
Anti-GAD67	Merck Millipore	Cat. No. MAB5406
Anti-GAD65/67	Thermofisher Invitrogen	Cat. No. PA1-84572
Anti-TH	Merck Millipore	Cat. No. MAB318
Anti-D2r	Merck Millipore	Cat. No. MABN53
Anti-DAT	Thermofisher Invitrogen	Cat. No. MA5-24796
Alexa-488 goat anti-mouse	Thermofisher Invitrogen	Cat. No. A-32723
Alexa-568 goat anti-mouse	Thermofisher Invitrogen	Cat. No. A-11004
Alexa-488 goat anti-rabbit	Thermofisher Invitrogen	Cat. No. A-11034
Alexa-647 goat anti-rabbit	Thermofisher Invitrogen	Cat. No. A-32733
AAV5-hSyn-eNpHR3.0-mCherry	NC Univ., USA.	n/a
AAV5-CaMKIIα-mCherry	NC Univ., USA.	n/a
Complete Freud’s adjuvant	Sigma Aldrich	Cat. No. F5881
45 mg sucrose pellets	Bioserv, USA.	Cat. No. F0023

## Data Availability

The datasets used or analyzed during the current study are available from the corresponding author on reasonable request.

## Supplementary Materials

The following supporting information can be downloaded at: https://www.mdpi.com/article/10.3390/biomedicines11030820/s1, Figure S1: Impact of light modulation of contralateral LHb excitatory neurons projecting into the VTA transfected with AAV5-CaMKIIα-mCherry (control virus) on behavioral responses. Source Data S1: Support raw data included in Figure 3, Source Data S2: Support raw data included in Figure 6.
