# Supplementary material for: Role of Glutamatergic Projections from Lateral Habenula to Ventral Tegmental Area in Inflammatory Pain-Related Spatial Working Memory Deficits
Source: Biomedicines. 2023 Mar 8;11(3):820. doi: 10.3390/biomedicines11030820 (PMC10045719; doi:10.3390/biomedicines11030820)
Supplement: Supplementary file 1 [file biomedicines-11-00820-s001.zip › FIGURE_S1_Supplementary_data.pdf]

Supplementary data

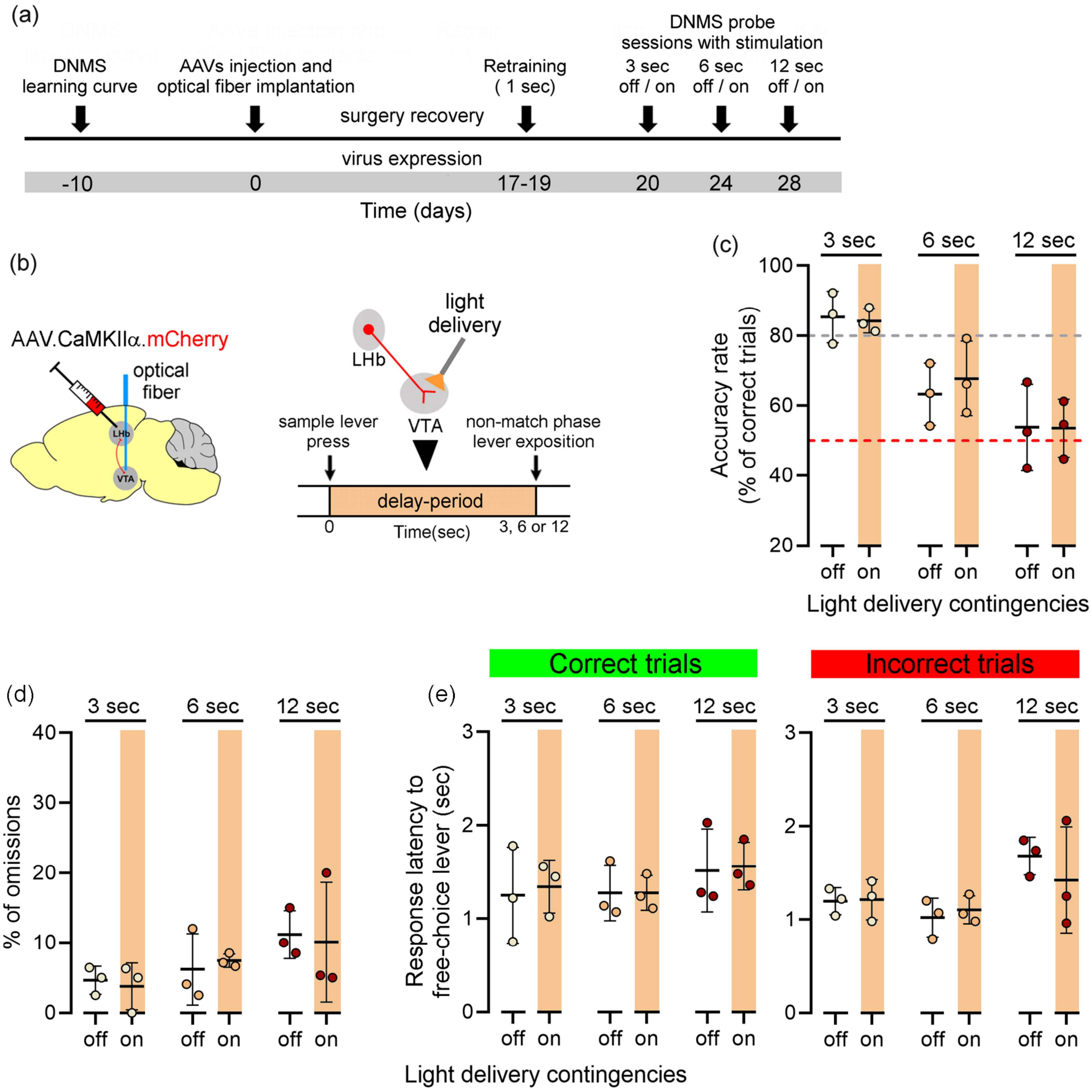

<u>FIGURE S1</u> – *Impact of light modulation of contralateral LHb excitatory neurons projecting into the VTA transfected with AAV5-CaMKIIα-mCherry (control virus) on behavioral responses.* (a) Timeline of experimental setup. **(b)** Strategy used for transfection of LHb glutamatergic neurons projecting into the VTA (naïve rats, *n*=3). Physical parameters of light modulation: continuous orange pulse at 620 nm, and intensity of 5-6 mW. Experimental data comparisons were based on two-way repeated measures ANOVA (F1: delay-period complexity x F2: light treatment). As expected, VTA light delivery has no significant effect on behavior performance for all tested DNMS delay-period

challenges. **(c)** Percentage of correct trials performed (F1: $F_{(1, 4)}$=0.04, *p*=0.8486; F2: $F_{(1.10, 4.41)}$=24.27, *p*=0.0058, and F1xF2: $F_{(2, 8)}$=0.23, *p*=0.8015), **(d)** percentage of omissions performed (F1: $F_{(1, 4)}$=0.01, *p*=0.9355; F2: $F_{(2, 8)}$=3.964, *p*=0.0636; and F1xF2: $F_{(2, 8)}$=0.15, *p*=0.8602), and **(e)** response latency to DNMS task free-choice lever in correct (left panel; F1: $F_{(1, 4)}$=0.04, *p*=0.8579; F2: $F_{(1.35, 5.39)}$=2.61, *p*=0.1639; and F1xF2: $F_{(2, 8)}$=0.07, *p*=0.9366) and incorrect trials (right panel; F1: $F_{(1, 4)}$=0.23, *p*=0.6563; F2: $F_{(1.68, 6.71)}$=3.94, *p*=0.0781; and F1xF2: $F_{(2, 8)}$=0.51, *p*=0.6183). Values are presented as mean ± S. D.
