# Supplementary figures and images for: Role of Glutamatergic Projections from Lateral Habenula to Ventral Tegmental Area in Inflammatory Pain-Related Spatial Working Memory Deficits
Source: Biomedicines. 2023 Mar 8;11(3):820. doi: 10.3390/biomedicines11030820 (PMC10045719; doi:10.3390/biomedicines11030820)

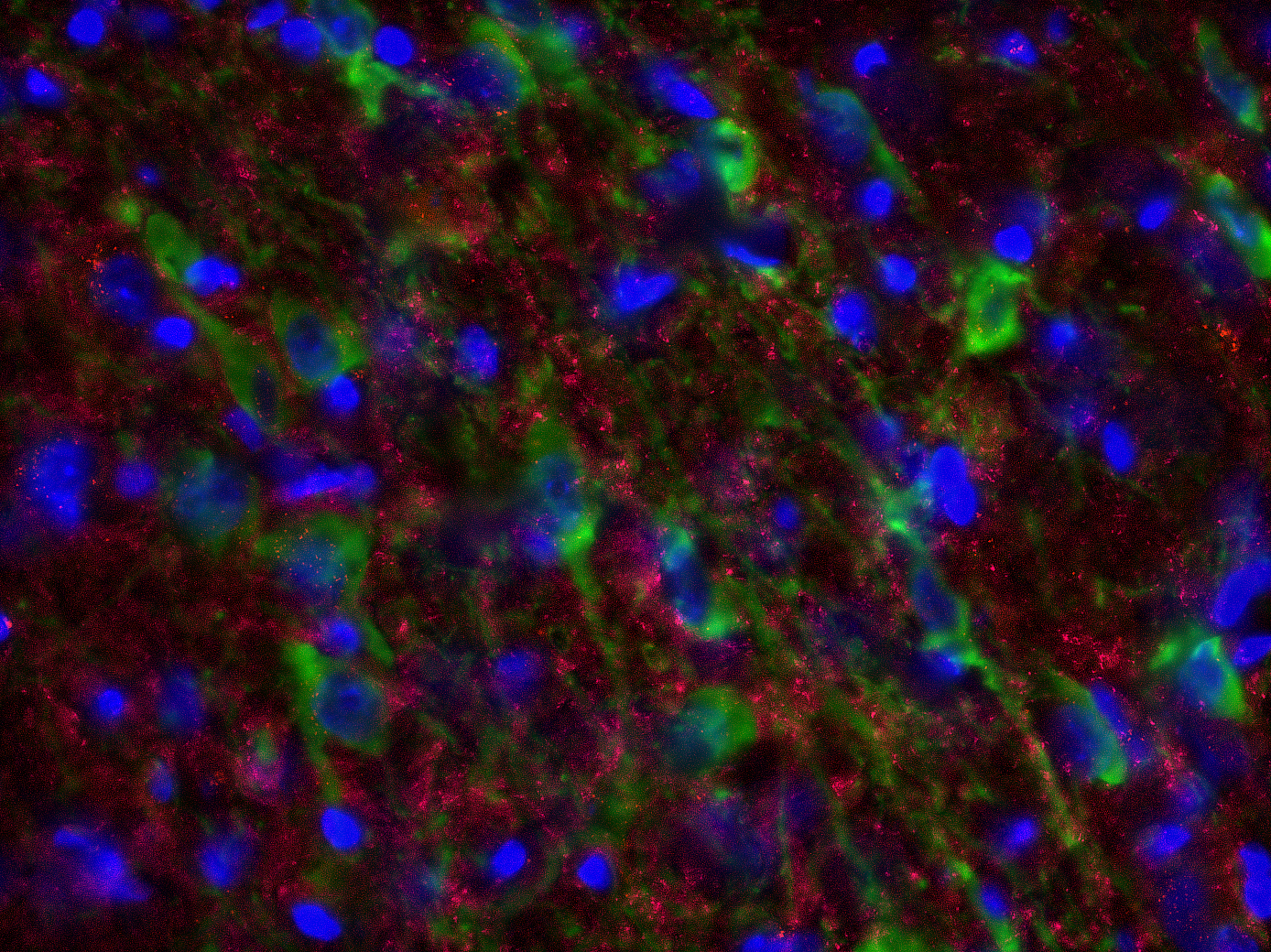

Supplement: Supplementary file 1 [file biomedicines-11-00820-s001.zip › Supplementary_Data_file_S1/Composite100-0014_Merge.png]

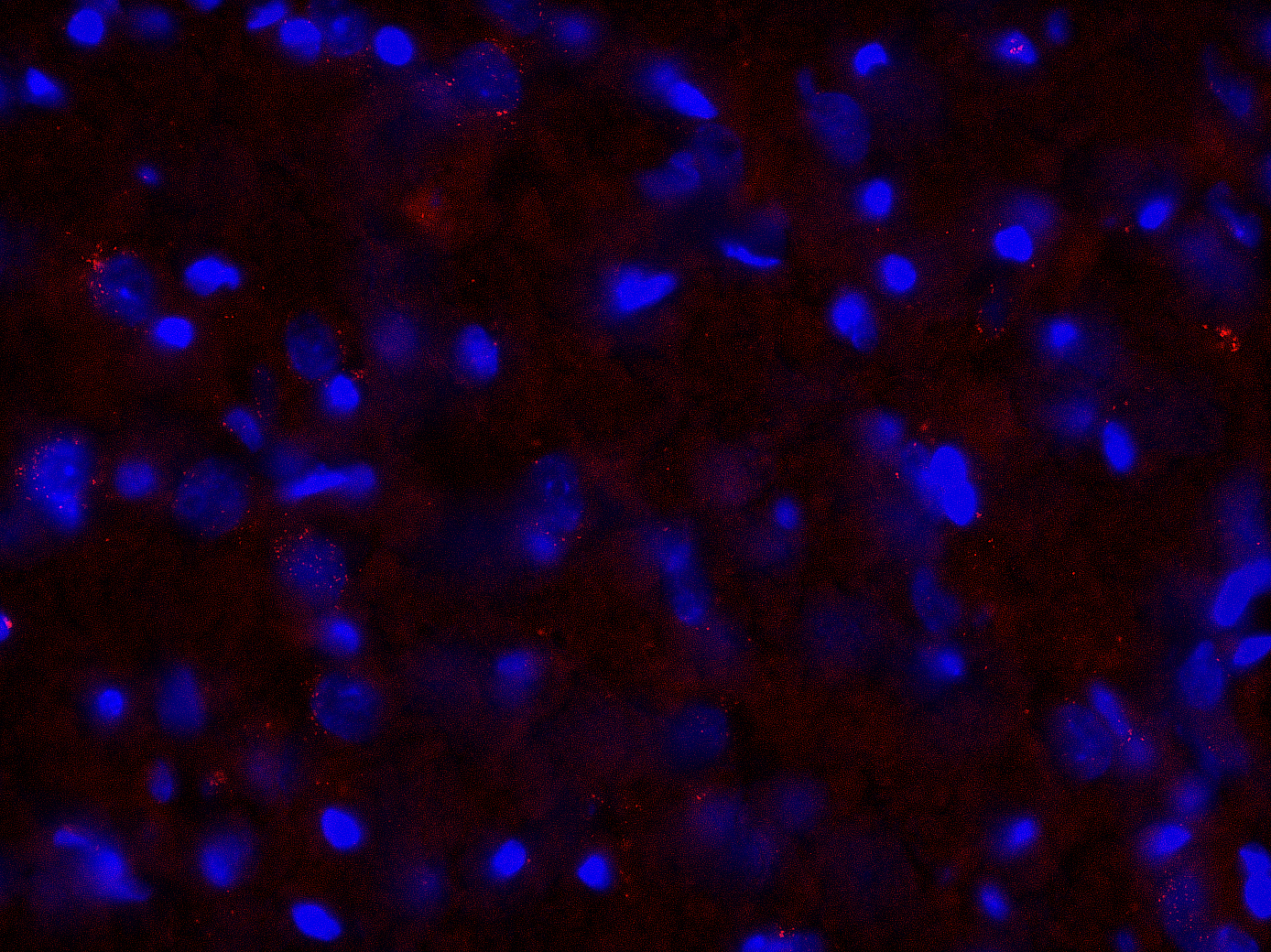

Supplement: Supplementary file 1 [file biomedicines-11-00820-s001.zip › Supplementary_Data_file_S1/Composite100-1-0014_mCherry.png]

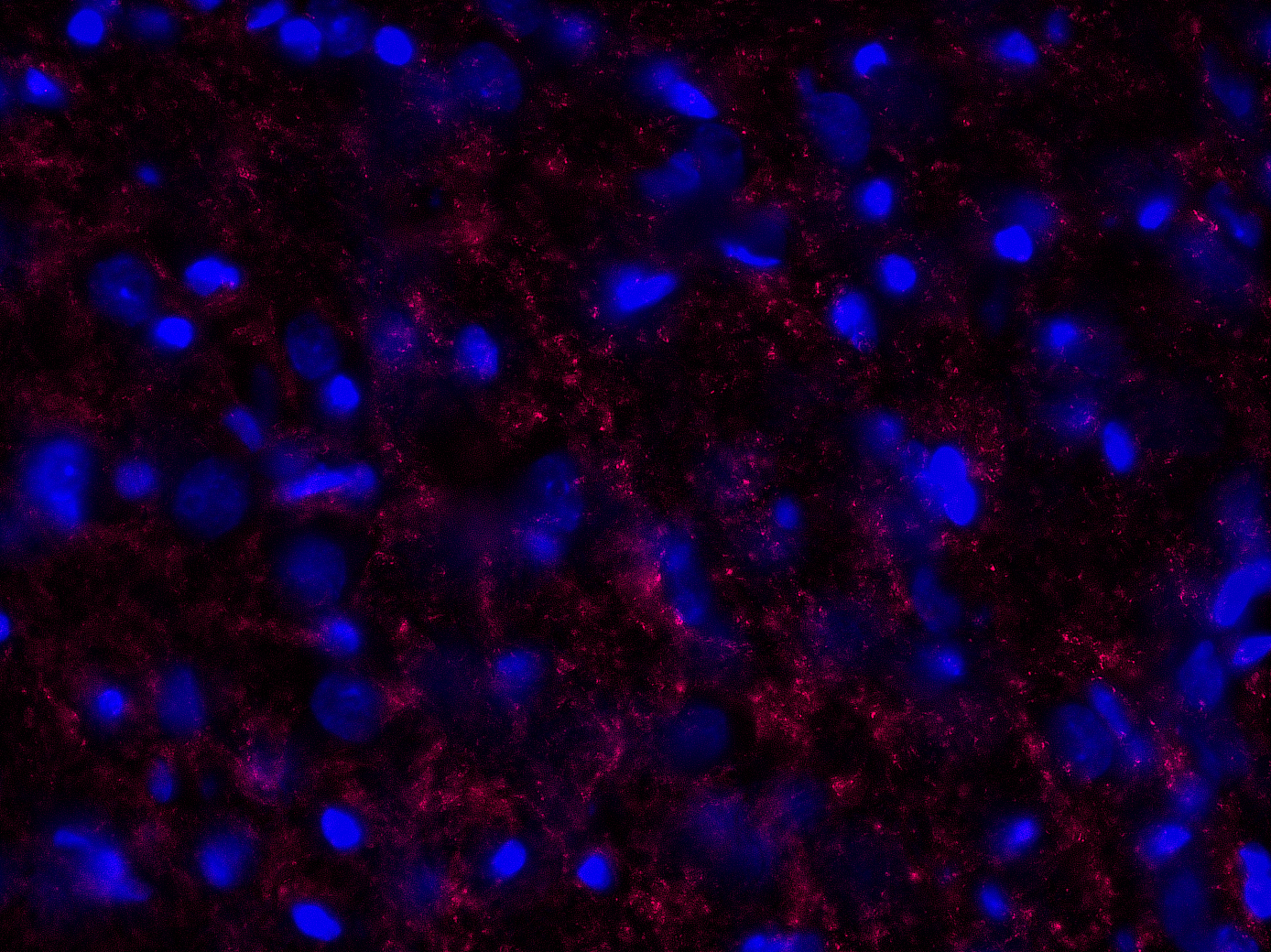

Supplement: Supplementary file 1 [file biomedicines-11-00820-s001.zip › Supplementary_Data_file_S1/Composite100-2-0014_GAD65_67.png]

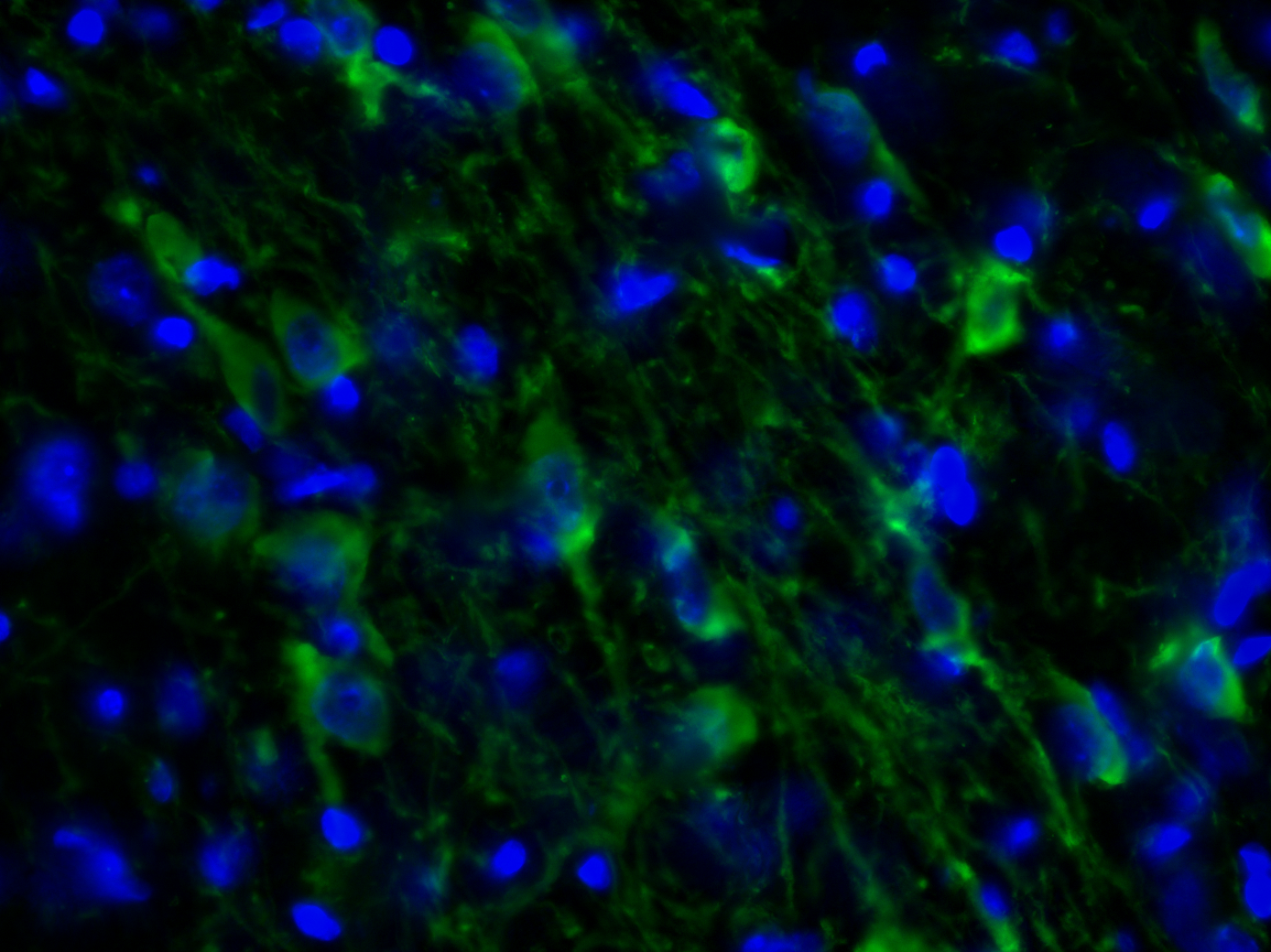

Supplement: Supplementary file 1 [file biomedicines-11-00820-s001.zip › Supplementary_Data_file_S1/Composite100-3-0014_TH.png]

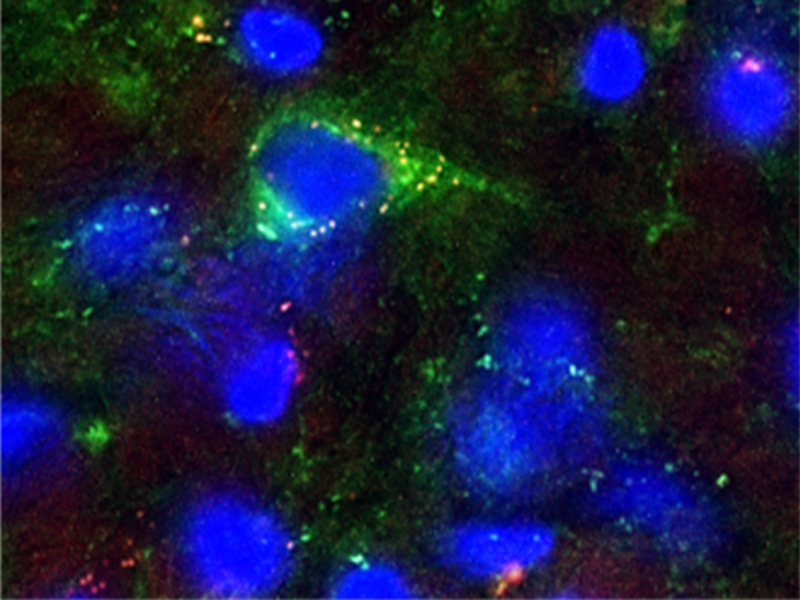

Supplement: Supplementary file 1 [file biomedicines-11-00820-s001.zip › Supplementary_Data_file_S1/Panel_Fig3a_GAD_LHb_terminals_mCherry.png]

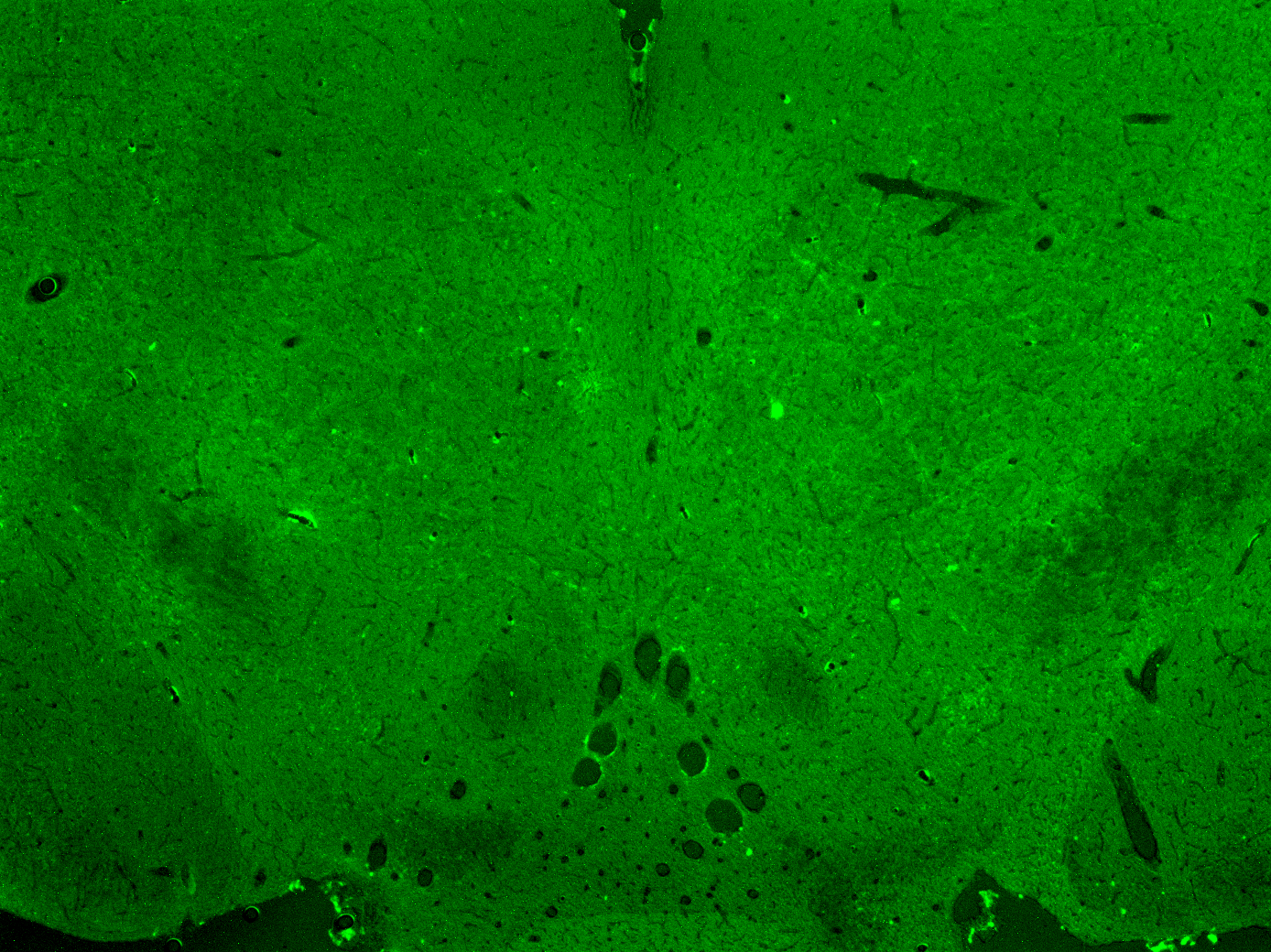

Supplement: Supplementary file 1 [file biomedicines-11-00820-s001.zip › Supplementary_Data_file_S2/D2r/PNG - D2R channel/CFA-LHb104-D2-488-VTA-2021-0005.zvi - C=1.png]

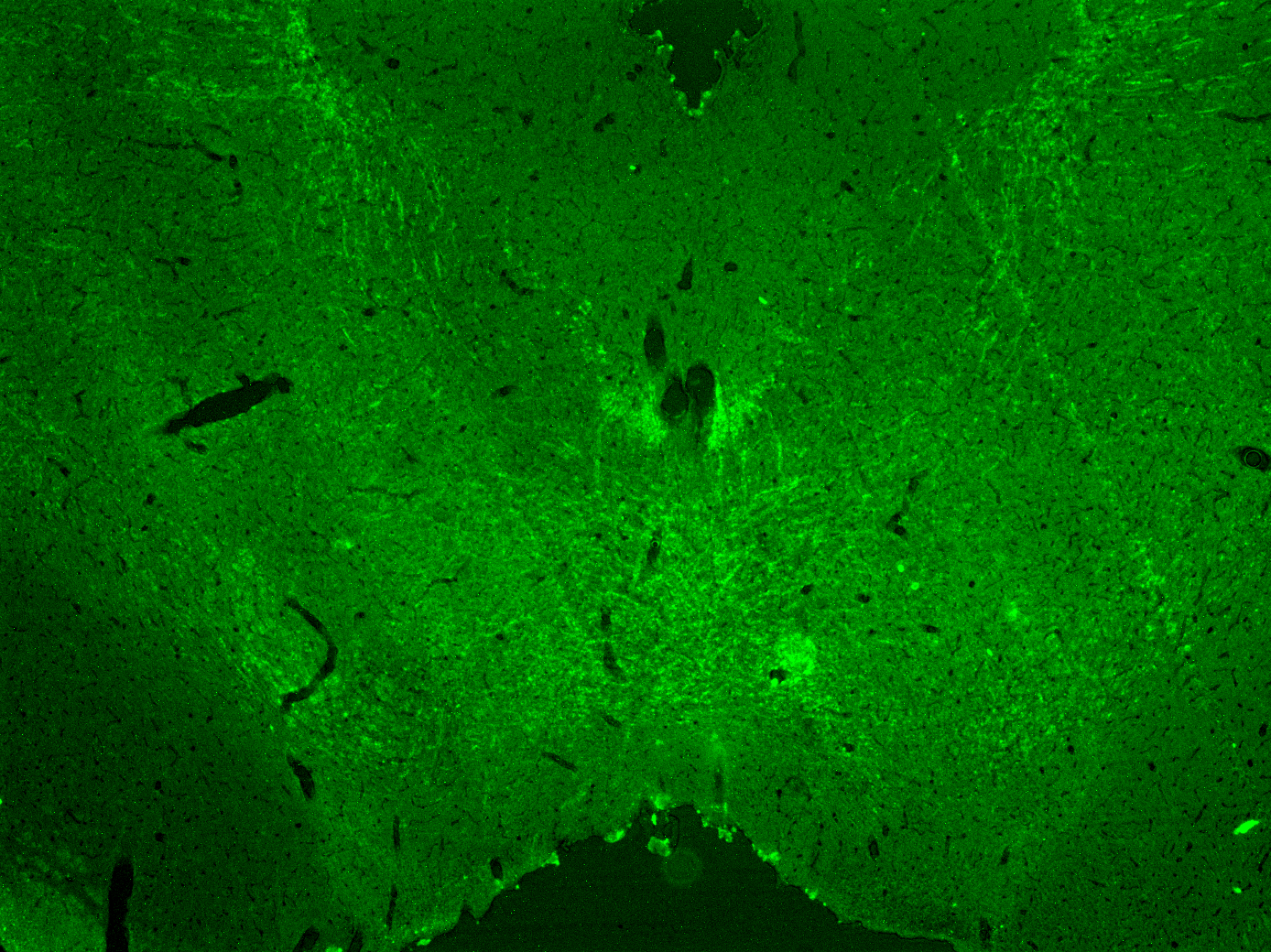

Supplement: Supplementary file 1 [file biomedicines-11-00820-s001.zip › Supplementary_Data_file_S2/D2r/PNG - D2R channel/CFA-LHb105-D2-488-VTA-2021-0006.zvi - C=1.png]

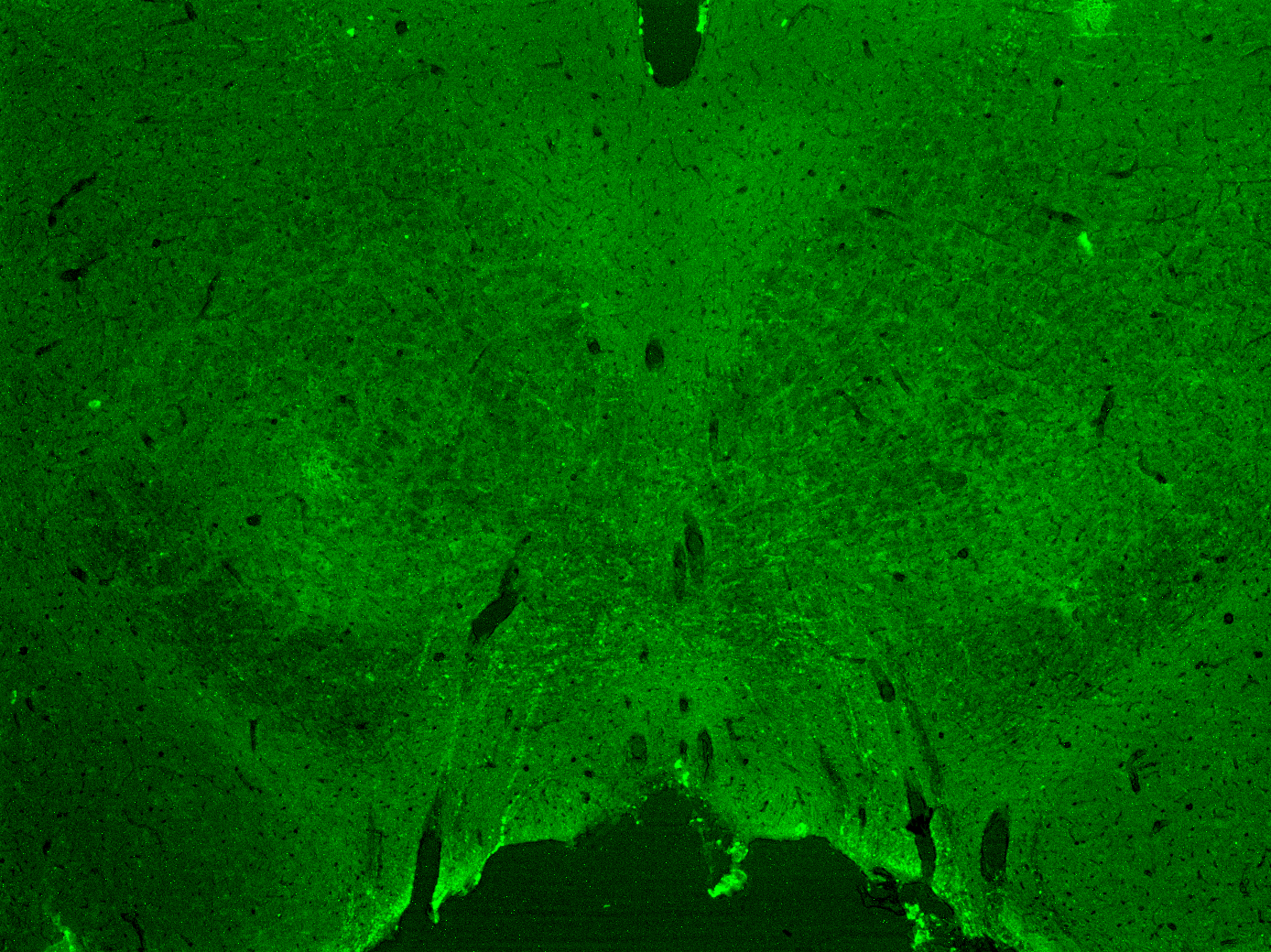

Supplement: Supplementary file 1 [file biomedicines-11-00820-s001.zip › Supplementary_Data_file_S2/D2r/PNG - D2R channel/CFA-LHb106-D2-488-VTA-2021-0007.zvi - C=1.png]

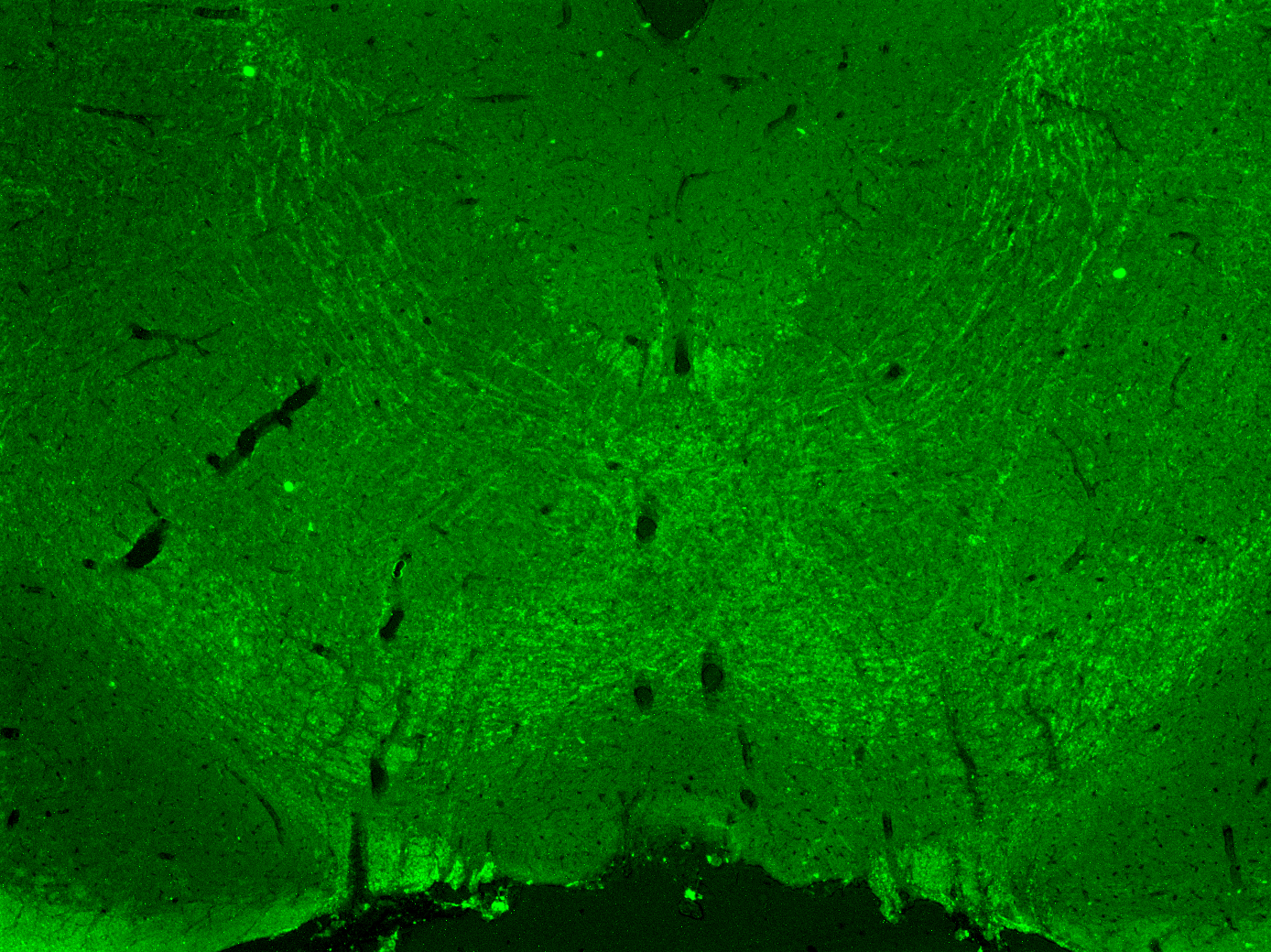

Supplement: Supplementary file 1 [file biomedicines-11-00820-s001.zip › Supplementary_Data_file_S2/D2r/PNG - D2R channel/CFA-LHb107-D2-488-VTA-2021-0008.zvi - C=1.png]

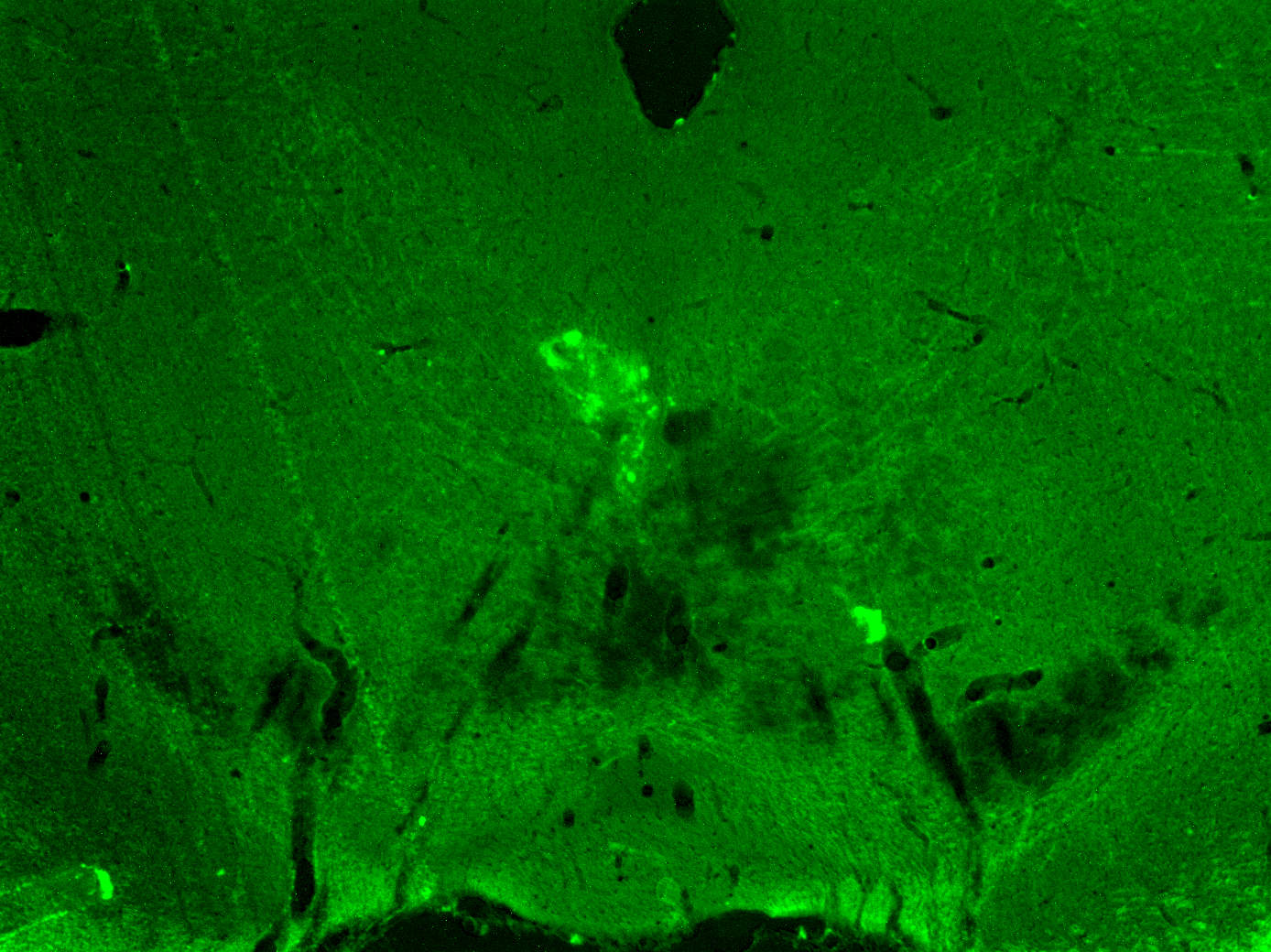

Supplement: Supplementary file 1 [file biomedicines-11-00820-s001.zip › Supplementary_Data_file_S2/D2r/PNG - D2R channel/CFA-LHb108-D2-488-VTA-2021-0010.zvi - C=1.png]

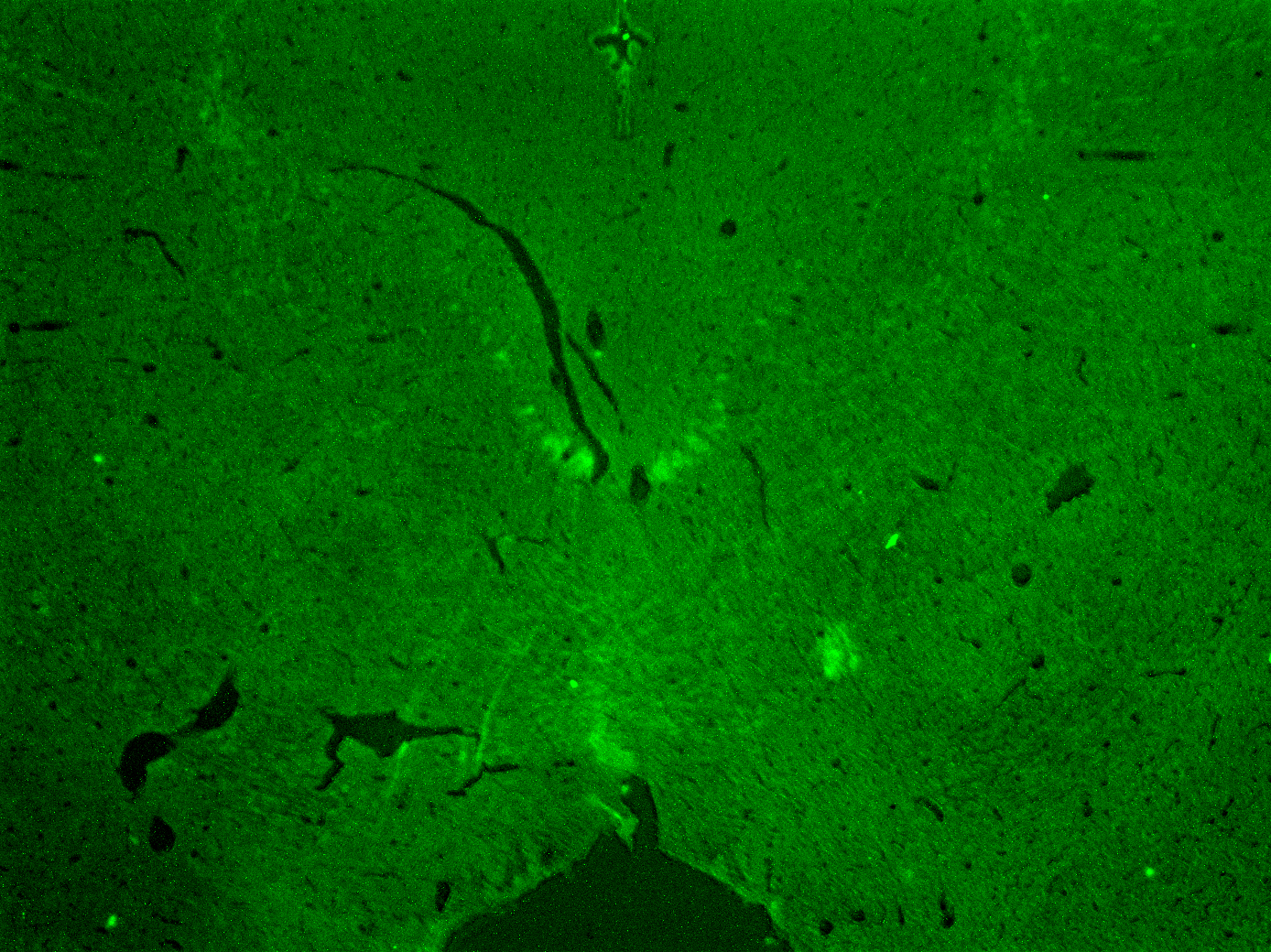

Supplement: Supplementary file 1 [file biomedicines-11-00820-s001.zip › Supplementary_Data_file_S2/D2r/PNG - D2R channel/CFA-LHb113-D2-488-VTA-2021-0015.zvi - C=1.png]

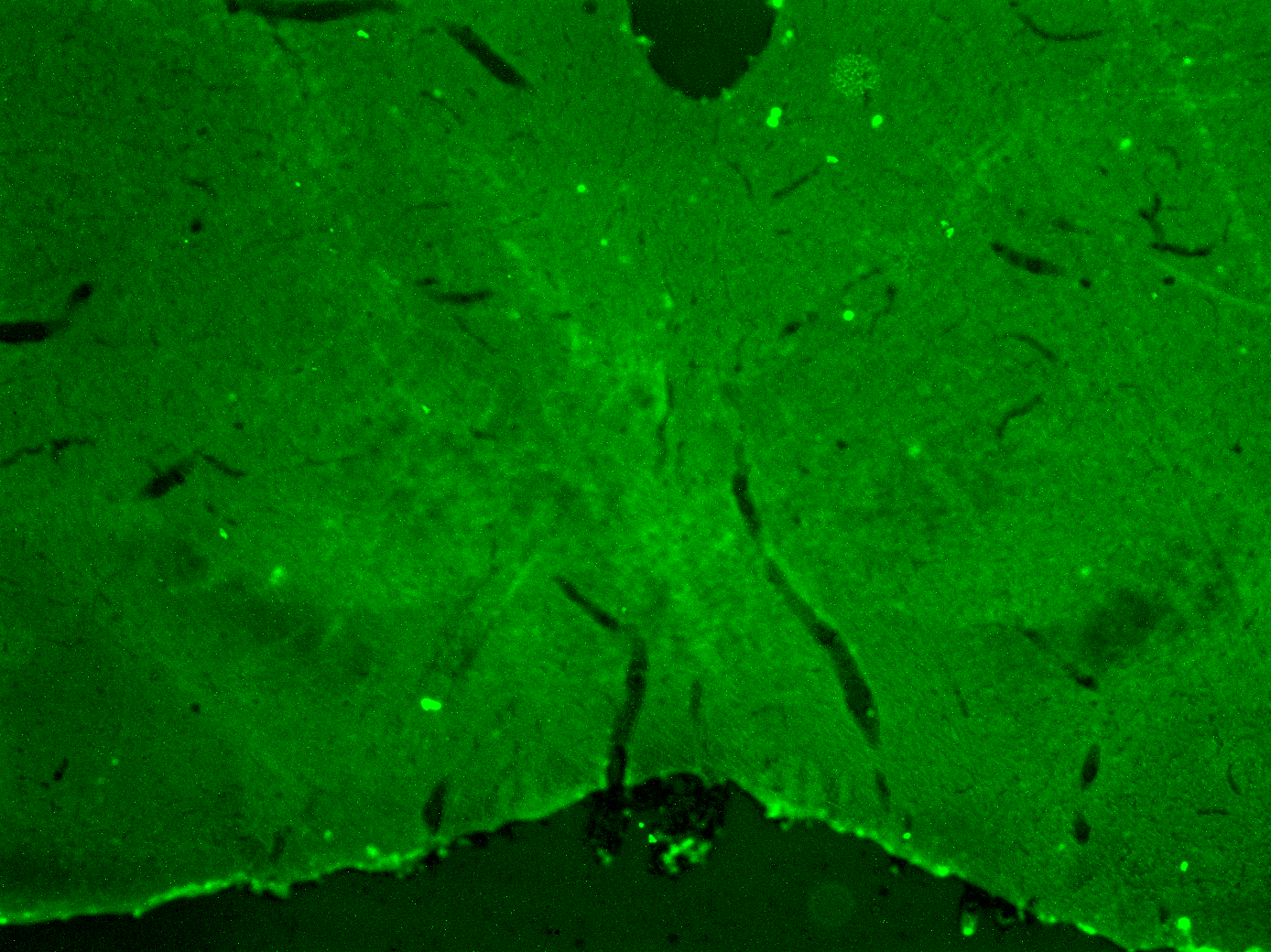

Supplement: Supplementary file 1 [file biomedicines-11-00820-s001.zip › Supplementary_Data_file_S2/D2r/PNG - D2R channel/CFA-LHb116-D2-488-VTA-2021-0017.zvi - C=1.png]

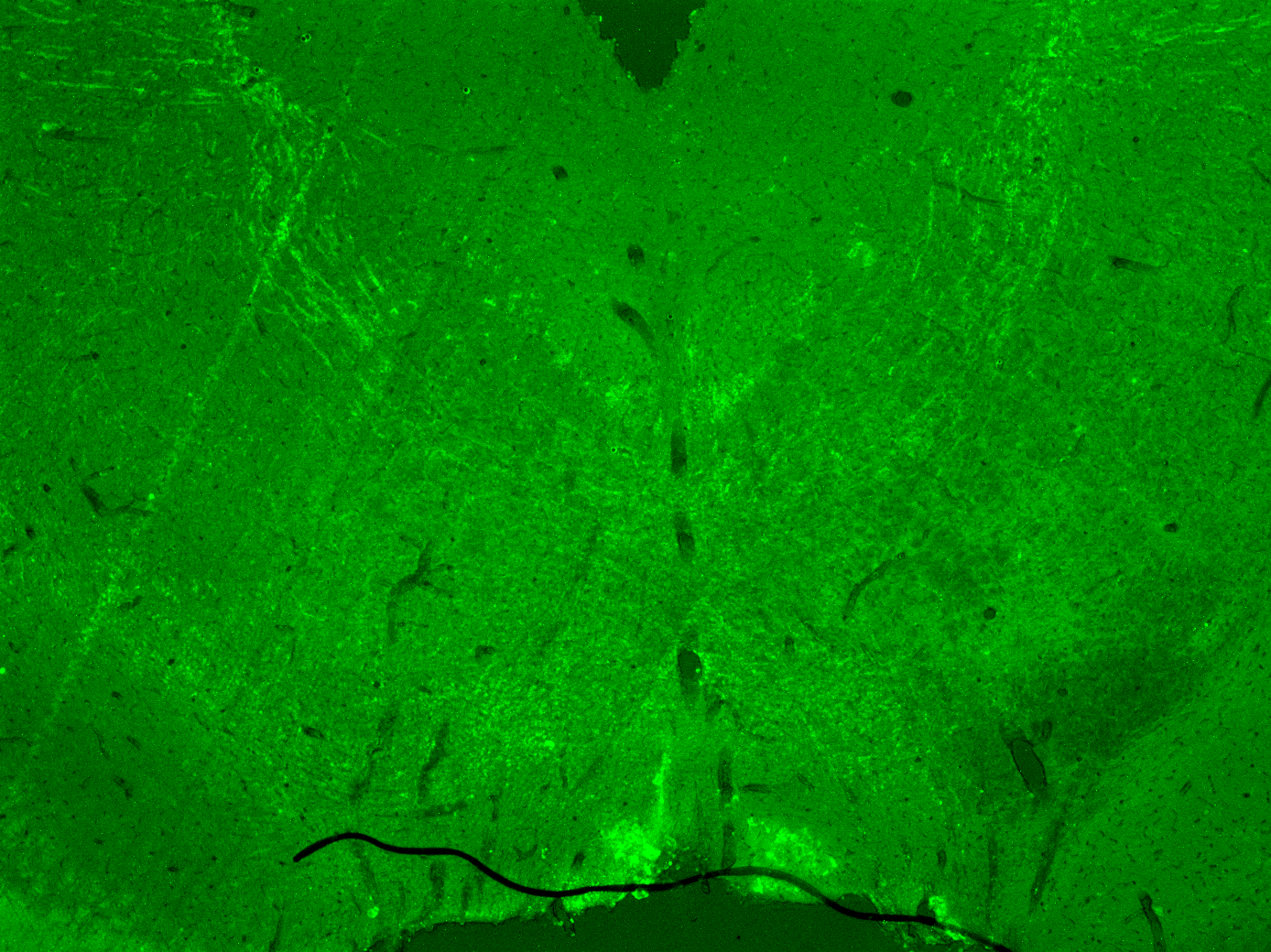

Supplement: Supplementary file 1 [file biomedicines-11-00820-s001.zip › Supplementary_Data_file_S2/D2r/PNG - D2R channel/SHAM-LHb100-D2-488-VTA-2021-0001.zvi - C=1.png]

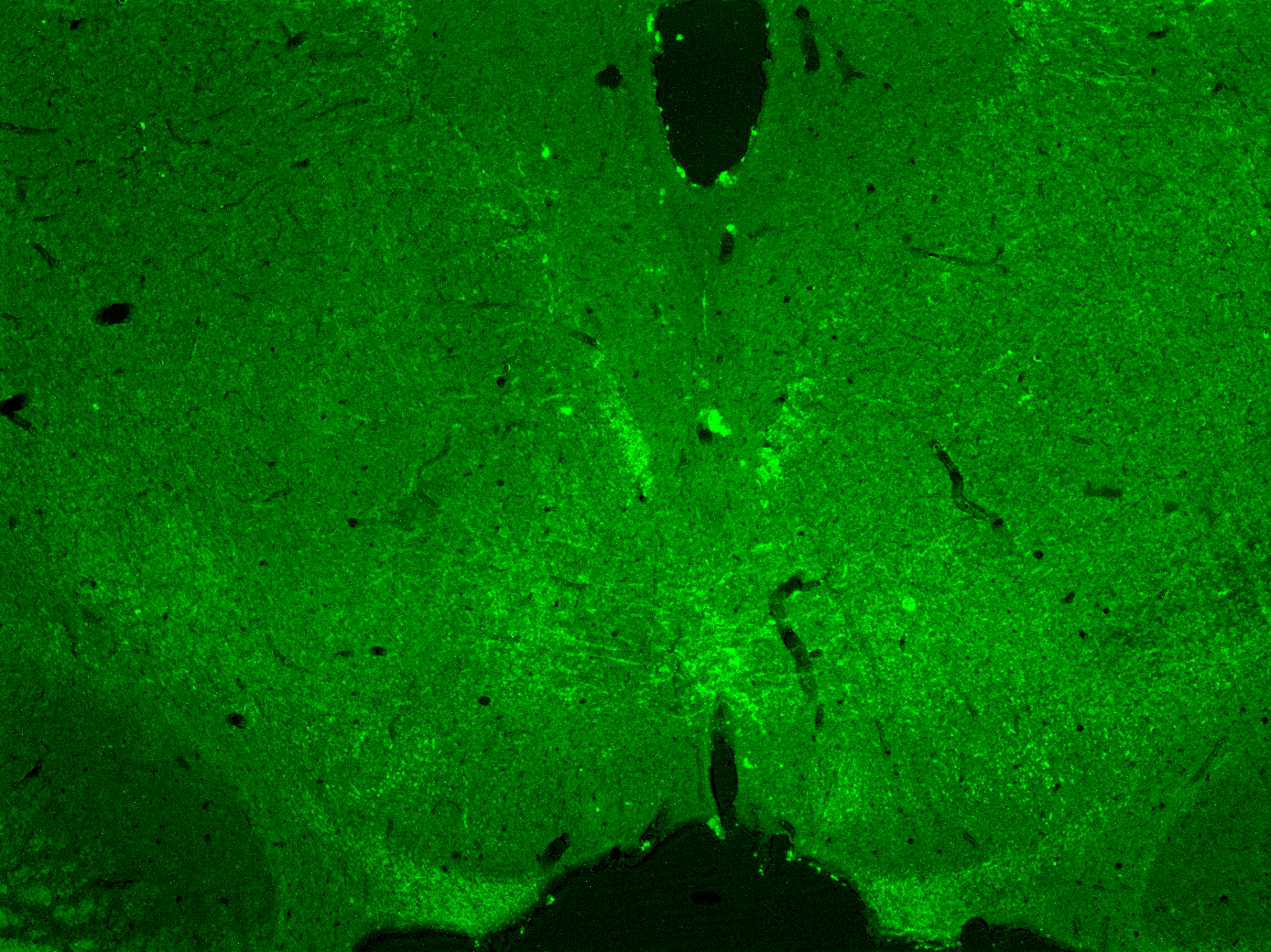

Supplement: Supplementary file 1 [file biomedicines-11-00820-s001.zip › Supplementary_Data_file_S2/D2r/PNG - D2R channel/SHAM-LHb101-D2-488-VTA-2021-0002.zvi - C=1.png]

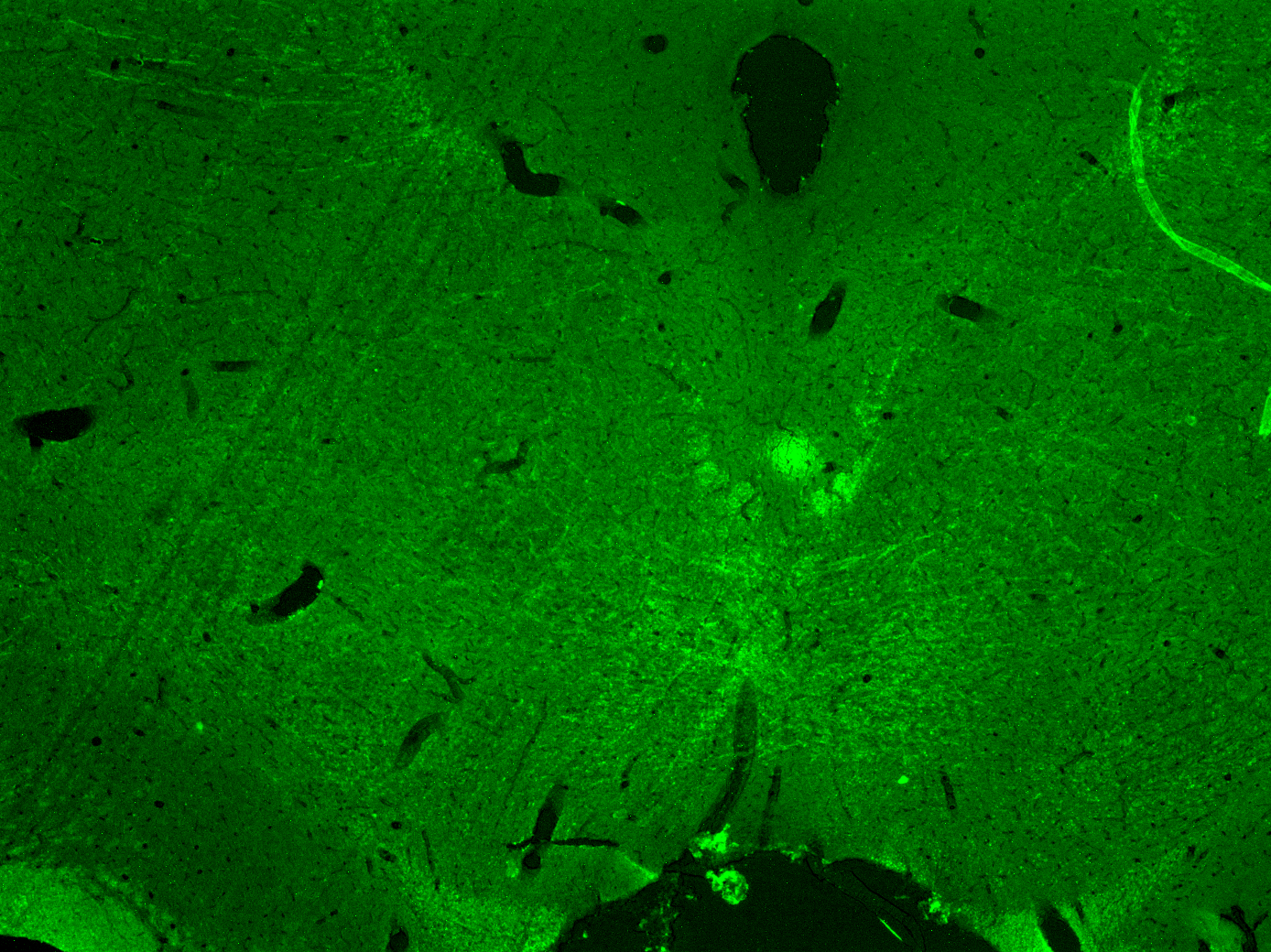

Supplement: Supplementary file 1 [file biomedicines-11-00820-s001.zip › Supplementary_Data_file_S2/D2r/PNG - D2R channel/SHAM-LHb103-D2-488-VTA-2021-0004.zvi - C=1.png]

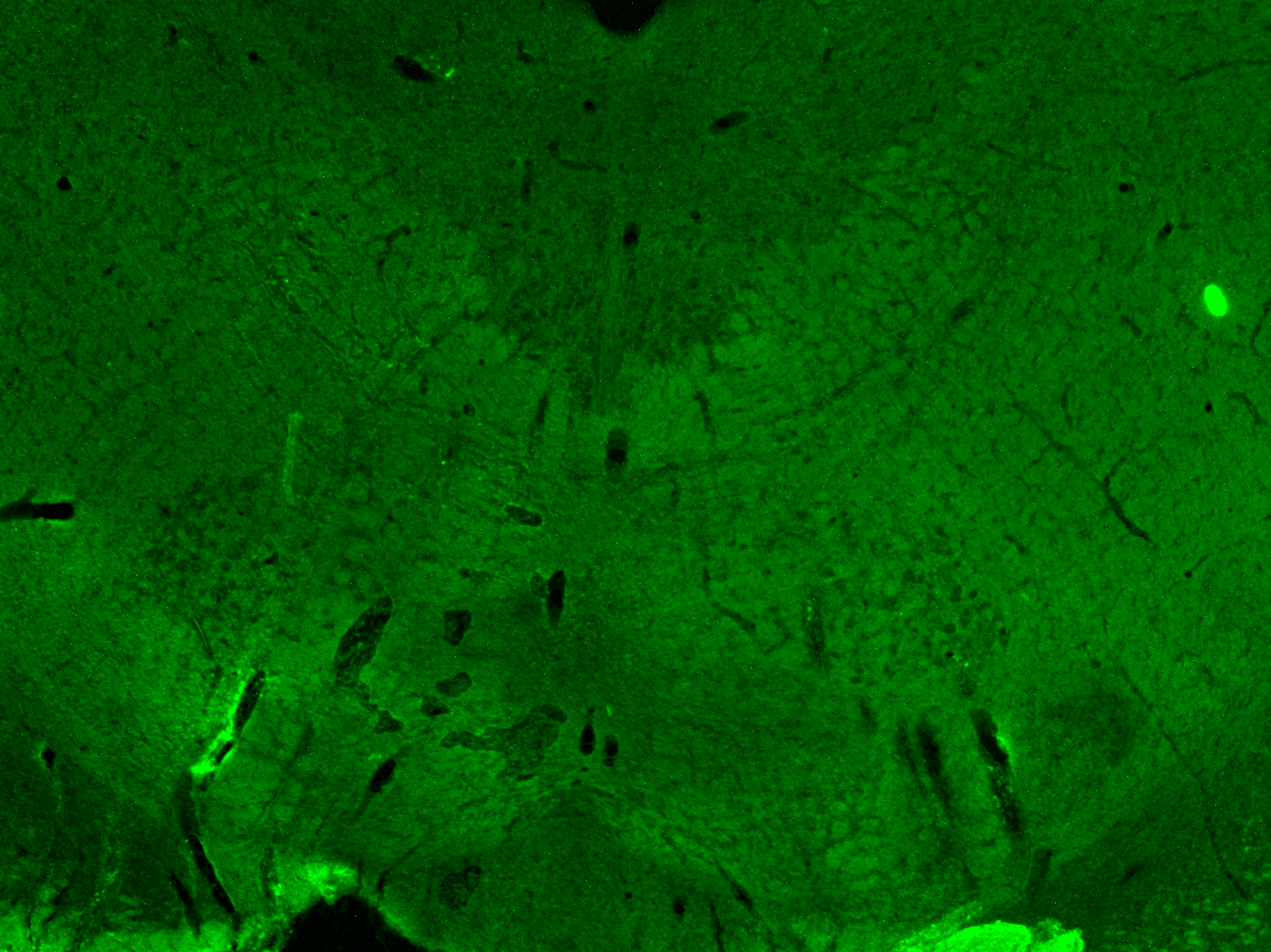

Supplement: Supplementary file 1 [file biomedicines-11-00820-s001.zip › Supplementary_Data_file_S2/D2r/PNG - D2R channel/SHAM-LHb120-D2-488-VTA-2021-0021.zvi - C=1.png]

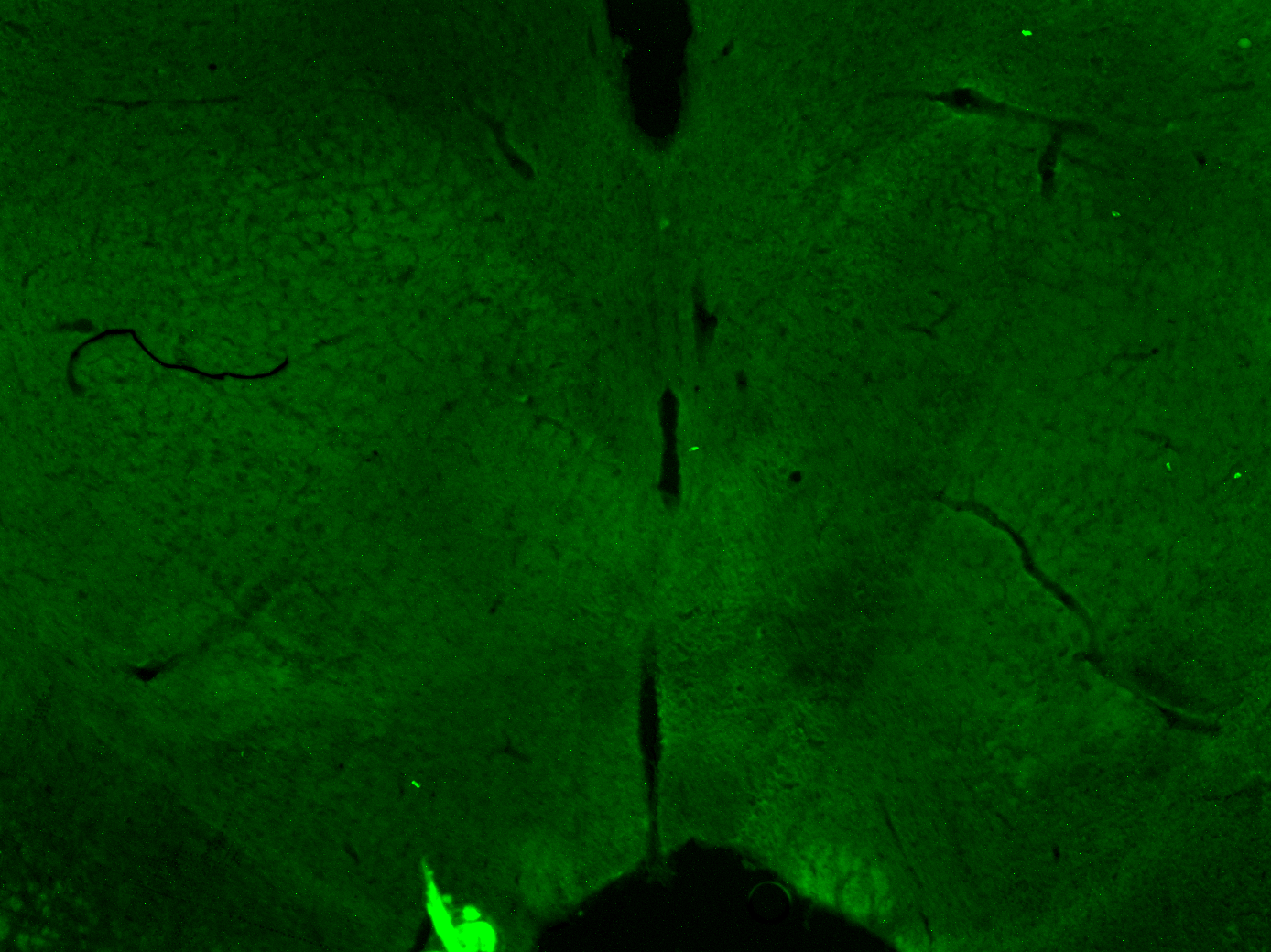

Supplement: Supplementary file 1 [file biomedicines-11-00820-s001.zip › Supplementary_Data_file_S2/D2r/PNG - D2R channel/SHAM-LHb121-D2-488-VTA-2021-0022.zvi - C=1.png]

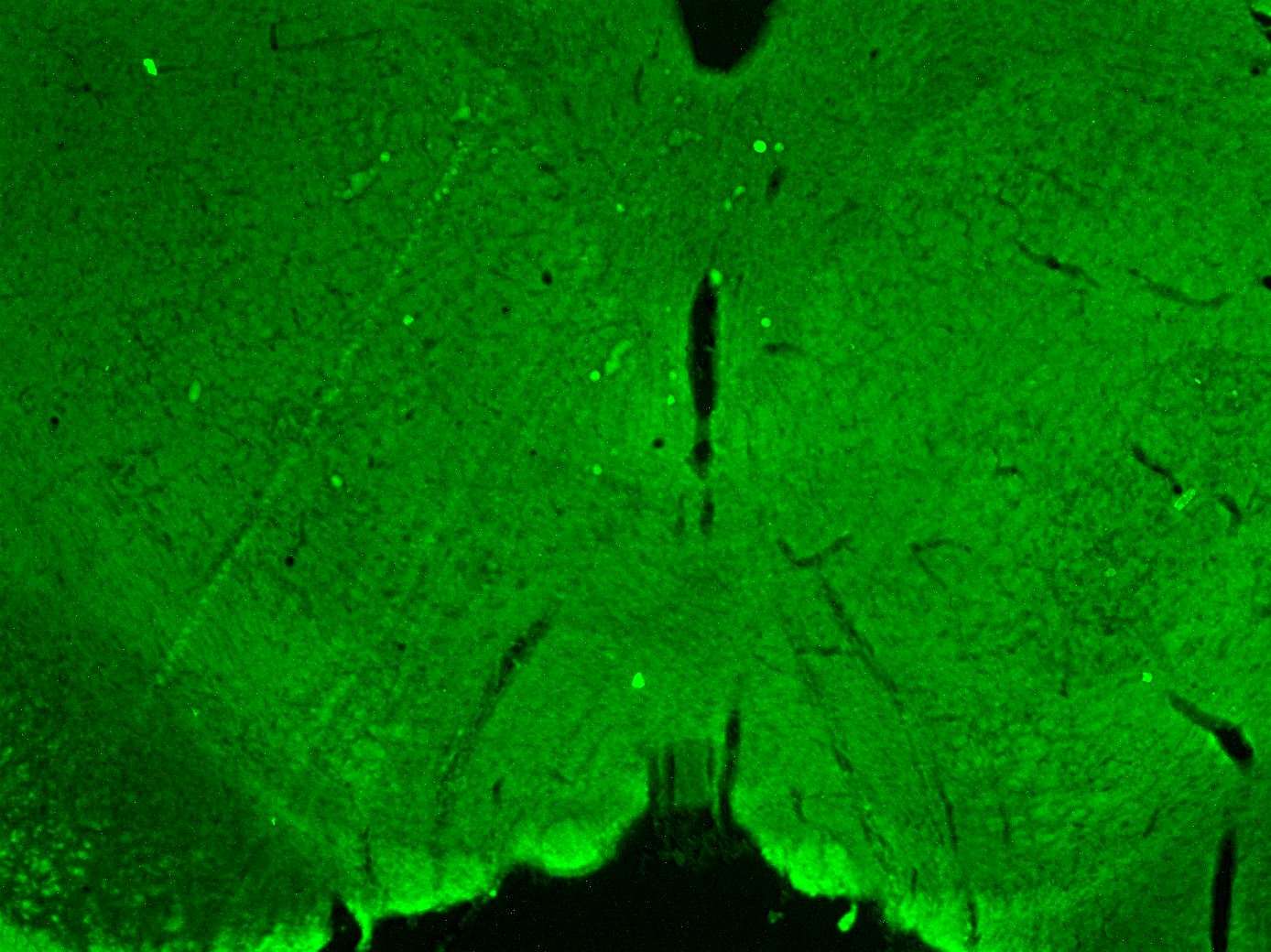

Supplement: Supplementary file 1 [file biomedicines-11-00820-s001.zip › Supplementary_Data_file_S2/D2r/PNG - D2R channel/SHAM-LHb122-D2-488-VTA-2021-0023.zvi - C=1.png]

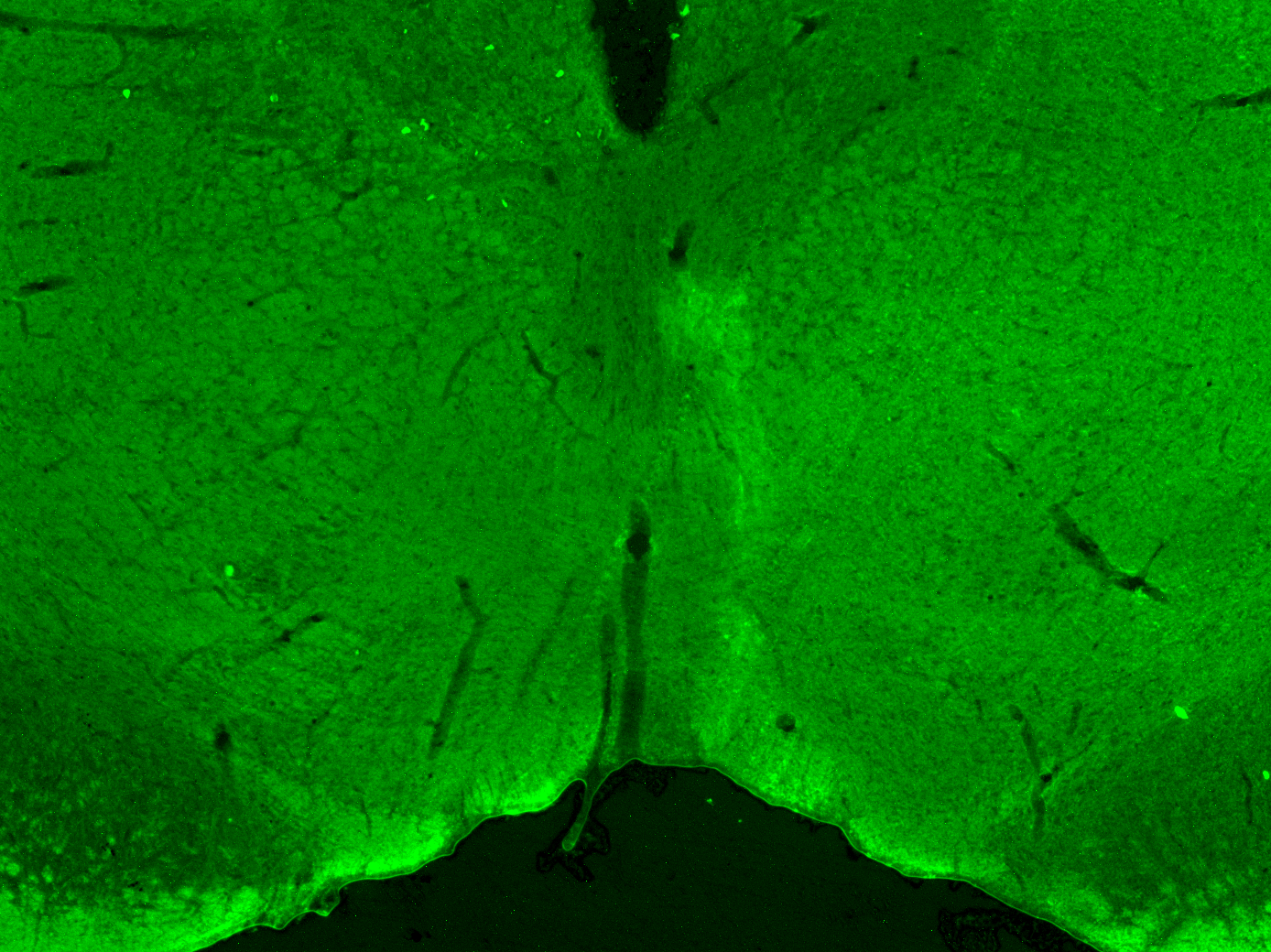

Supplement: Supplementary file 1 [file biomedicines-11-00820-s001.zip › Supplementary_Data_file_S2/D2r/PNG - D2R channel/SHAM-LHb123-D2-488-VTA-2021-0024.zvi - C=1.png]

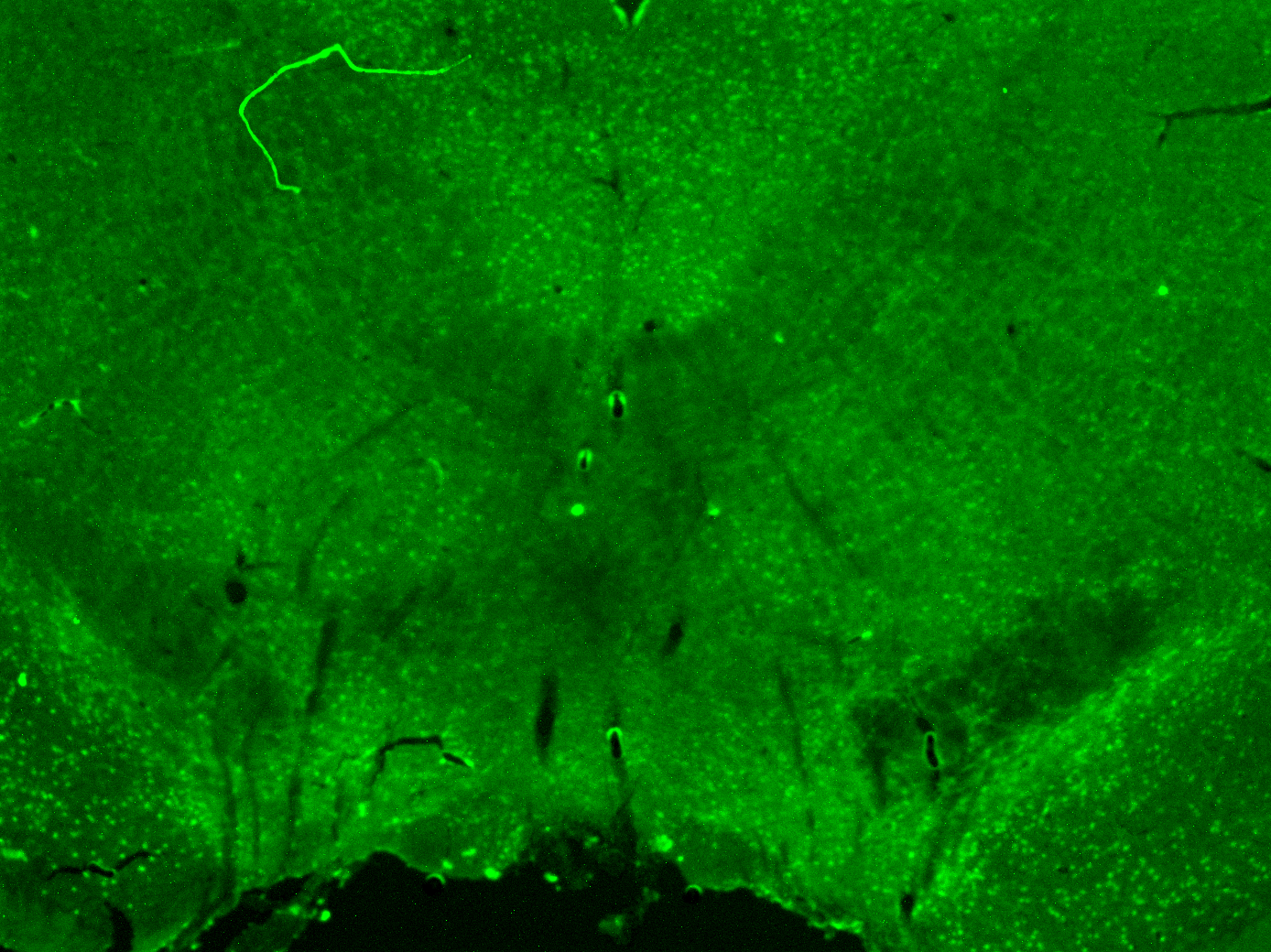

Supplement: Supplementary file 1 [file biomedicines-11-00820-s001.zip › Supplementary_Data_file_S2/DAT/PNG - DAT channel/CFA-LHb104-DAT-488-VTA-2021-0006.zvi - C=1.png]

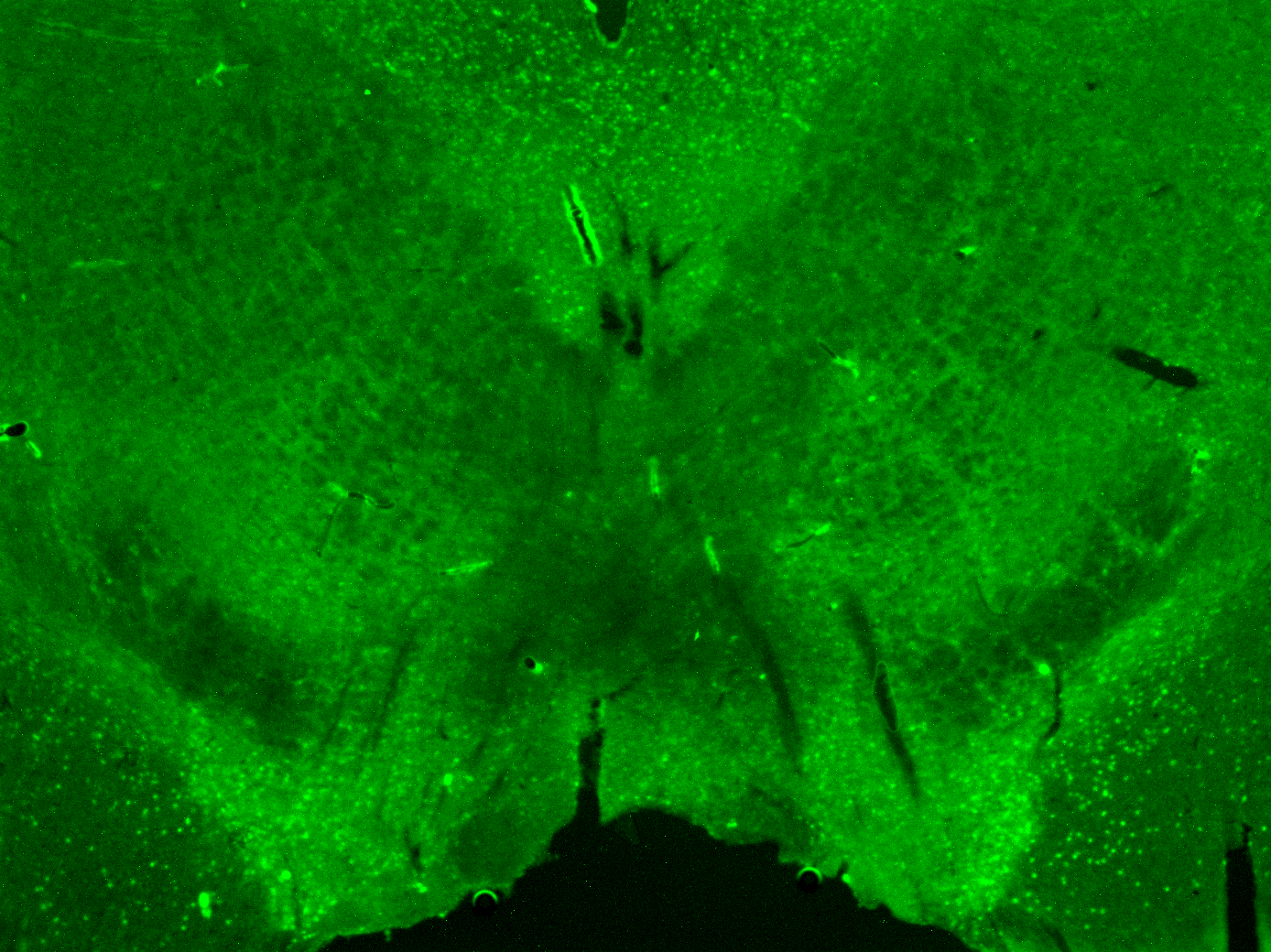

Supplement: Supplementary file 1 [file biomedicines-11-00820-s001.zip › Supplementary_Data_file_S2/DAT/PNG - DAT channel/CFA-LHb105-DAT-488-VTA-2021-0007.zvi - C=1.png]

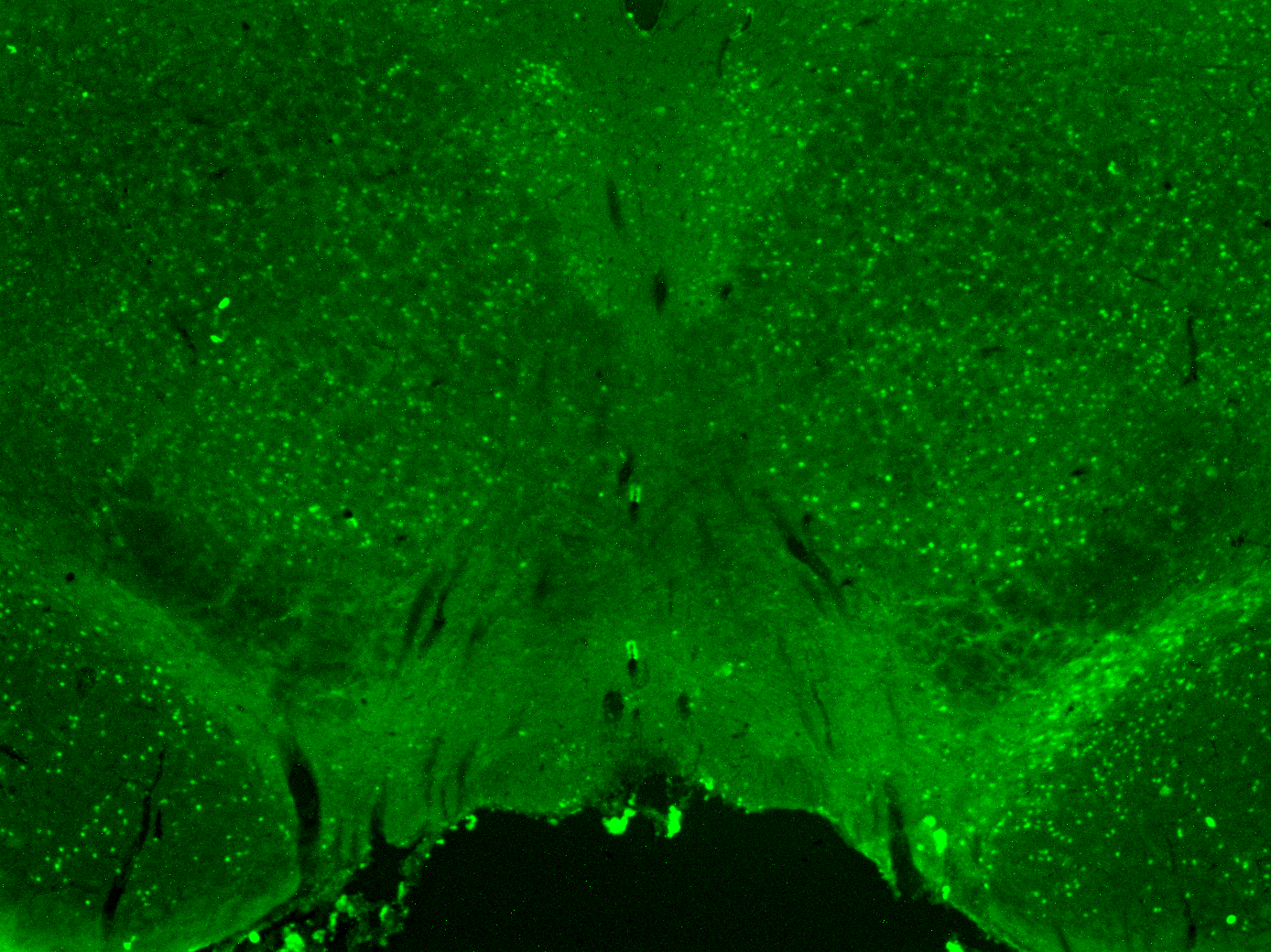

Supplement: Supplementary file 1 [file biomedicines-11-00820-s001.zip › Supplementary_Data_file_S2/DAT/PNG - DAT channel/CFA-LHb106-DAT-488-VTA-2021-0008.zvi - C=1.png]

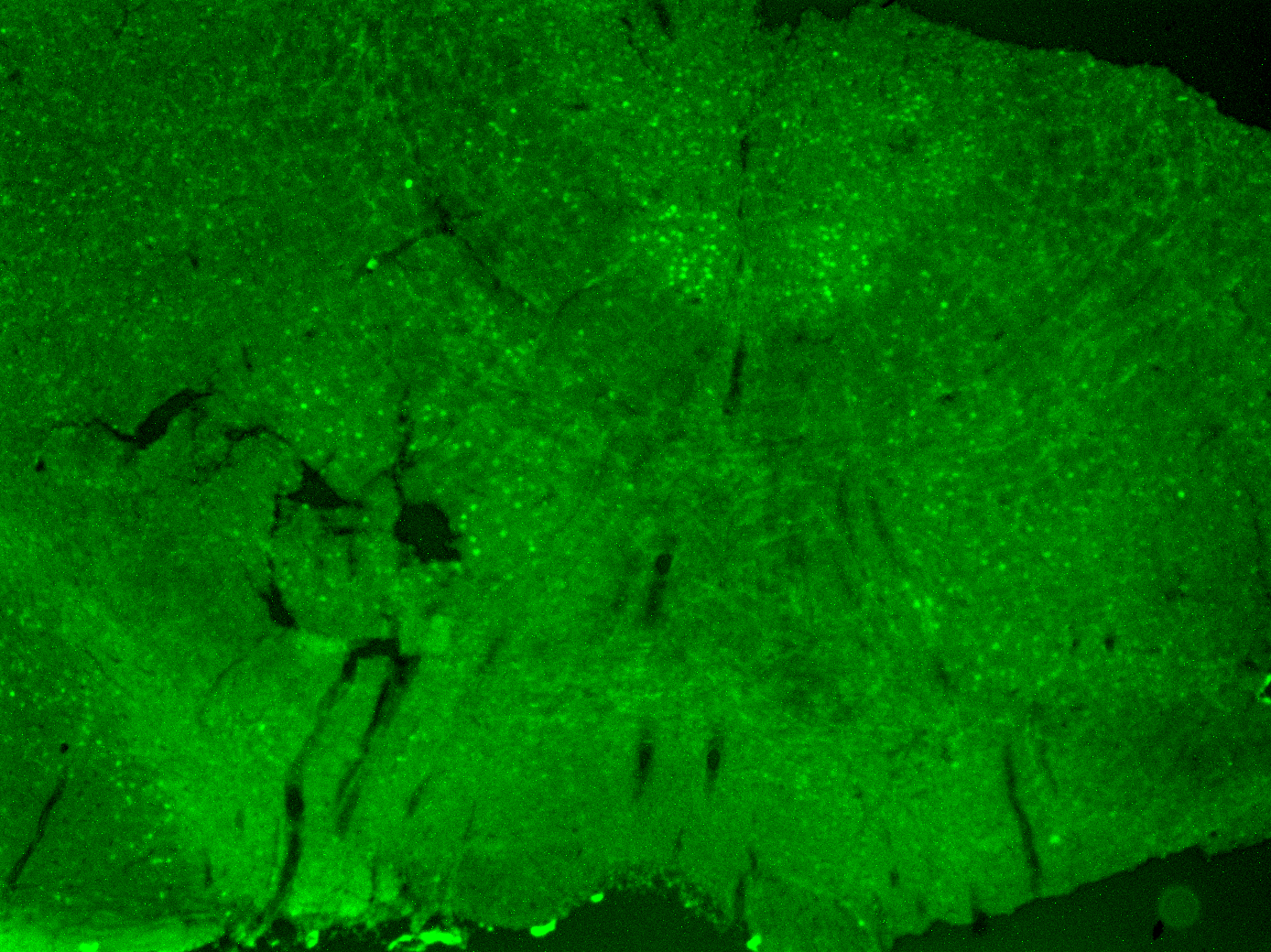

Supplement: Supplementary file 1 [file biomedicines-11-00820-s001.zip › Supplementary_Data_file_S2/DAT/PNG - DAT channel/CFA-LHb107-DAT-488-VTA-2021-0009.zvi - C=1.png]

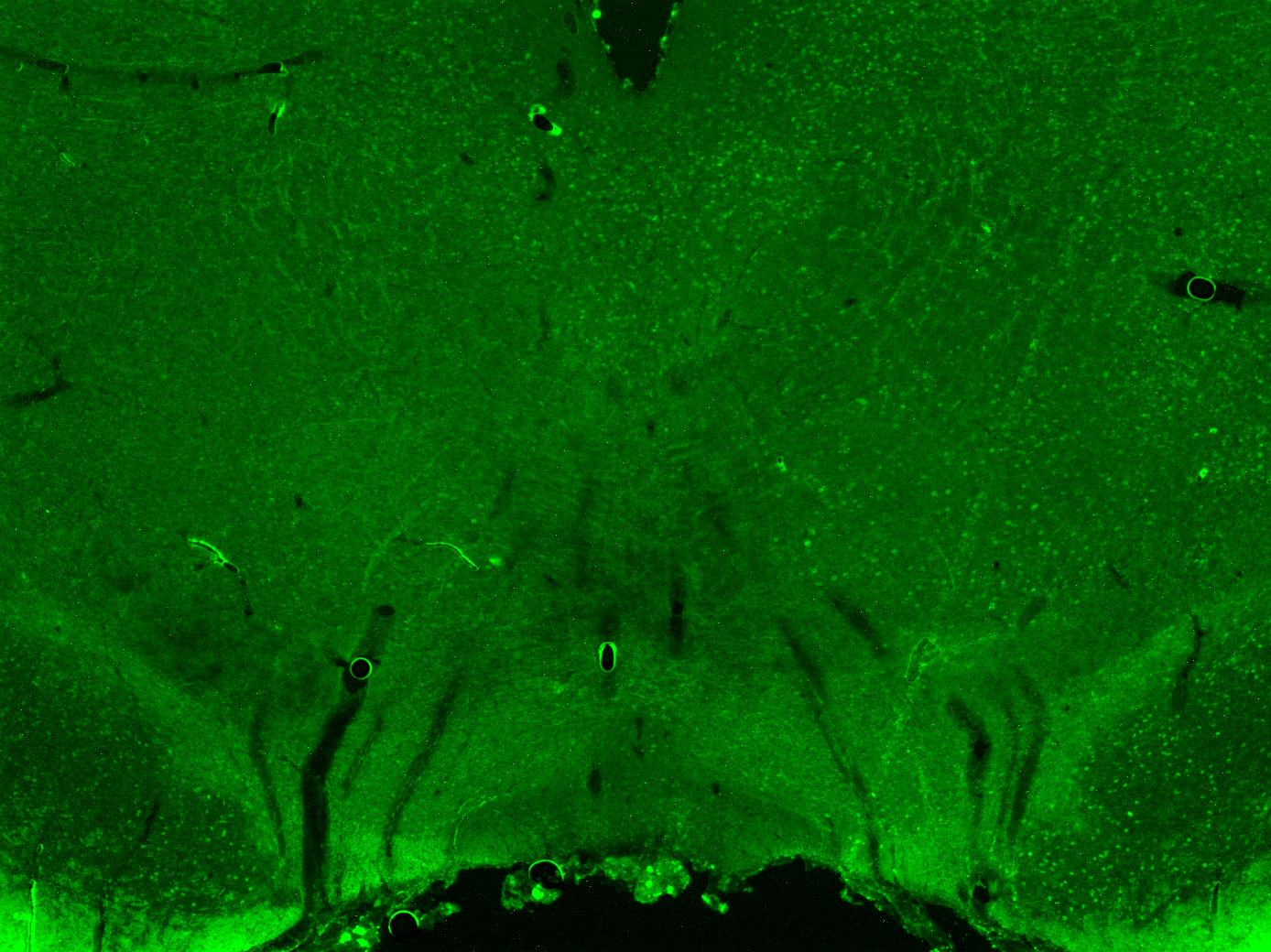

Supplement: Supplementary file 1 [file biomedicines-11-00820-s001.zip › Supplementary_Data_file_S2/DAT/PNG - DAT channel/CFA-LHb108-DAT-488-VTA-2021-0010.zvi - C=1.png]

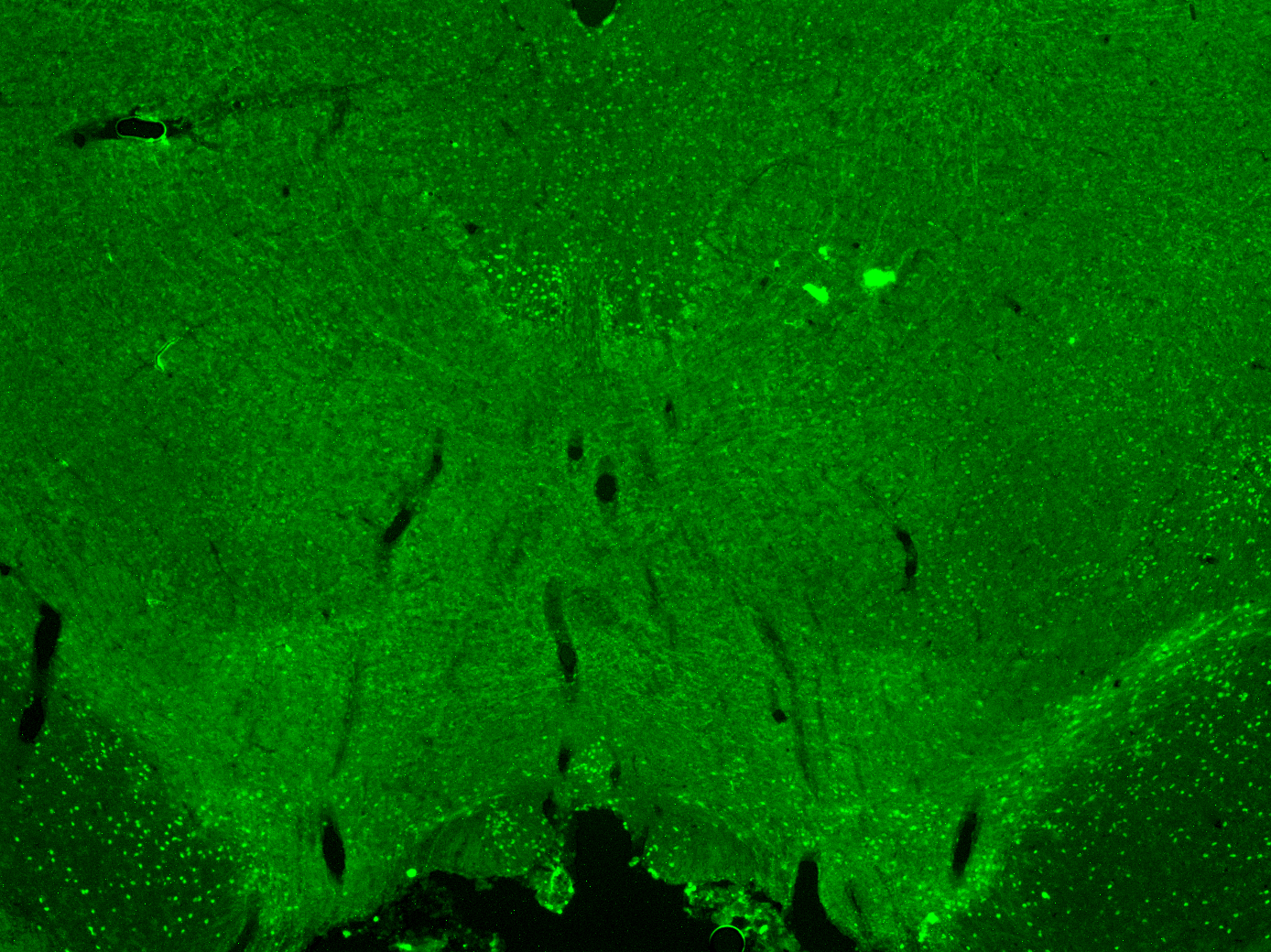

Supplement: Supplementary file 1 [file biomedicines-11-00820-s001.zip › Supplementary_Data_file_S2/DAT/PNG - DAT channel/CFA-LHb112-DAT-488-VTA-2021-0014.zvi - C=1.png]

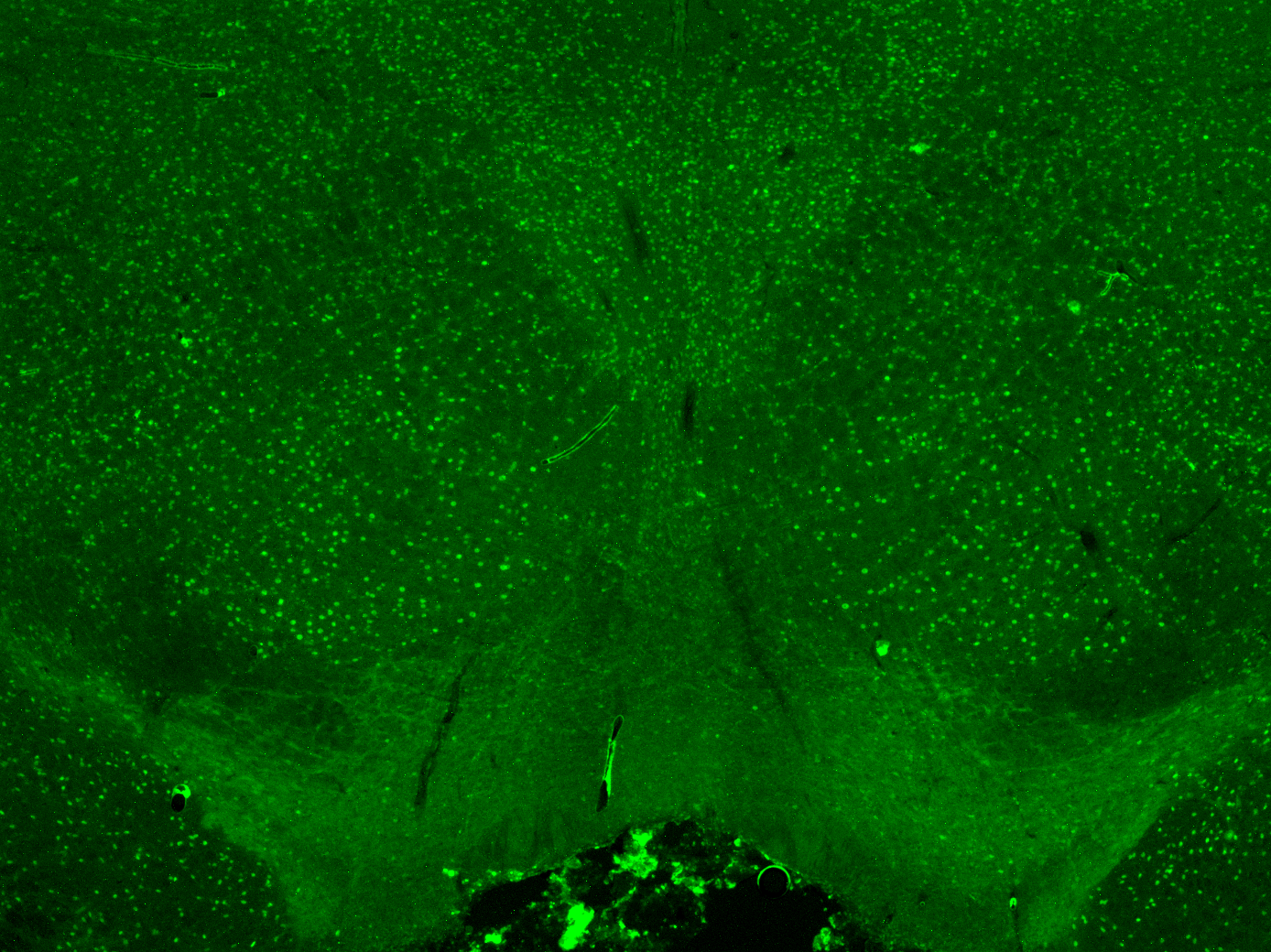

Supplement: Supplementary file 1 [file biomedicines-11-00820-s001.zip › Supplementary_Data_file_S2/DAT/PNG - DAT channel/CFA-LHb113-DAT-488-VTA-2021-0015.zvi - C=1.png]

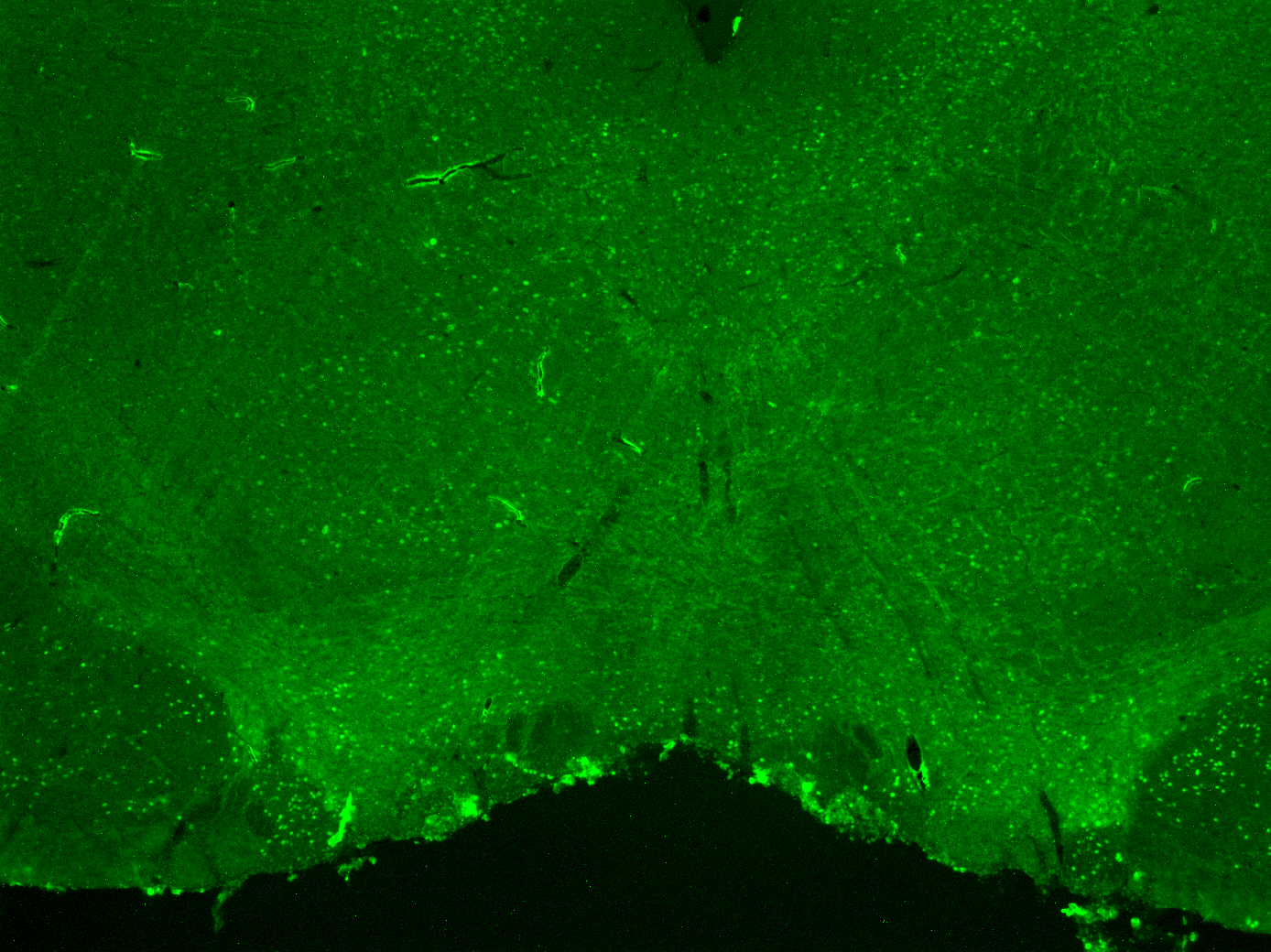

Supplement: Supplementary file 1 [file biomedicines-11-00820-s001.zip › Supplementary_Data_file_S2/DAT/PNG - DAT channel/CFA-LHb116-DAT-488-VTA-2021-0017.zvi - C=1.png]

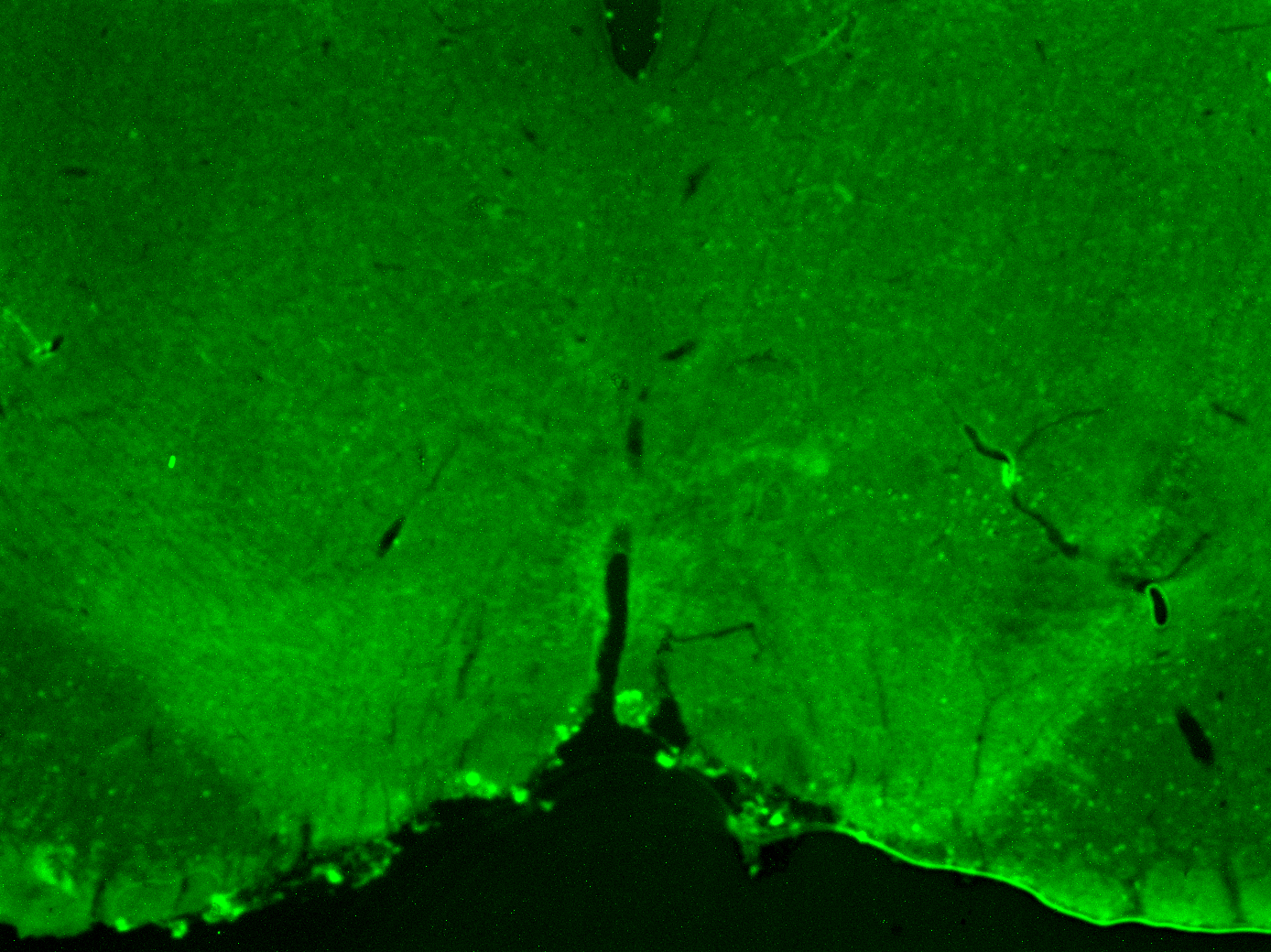

Supplement: Supplementary file 1 [file biomedicines-11-00820-s001.zip › Supplementary_Data_file_S2/DAT/PNG - DAT channel/CFA-LHb118-DAT-488-VTA-2021-0019.zvi - C=1.png]

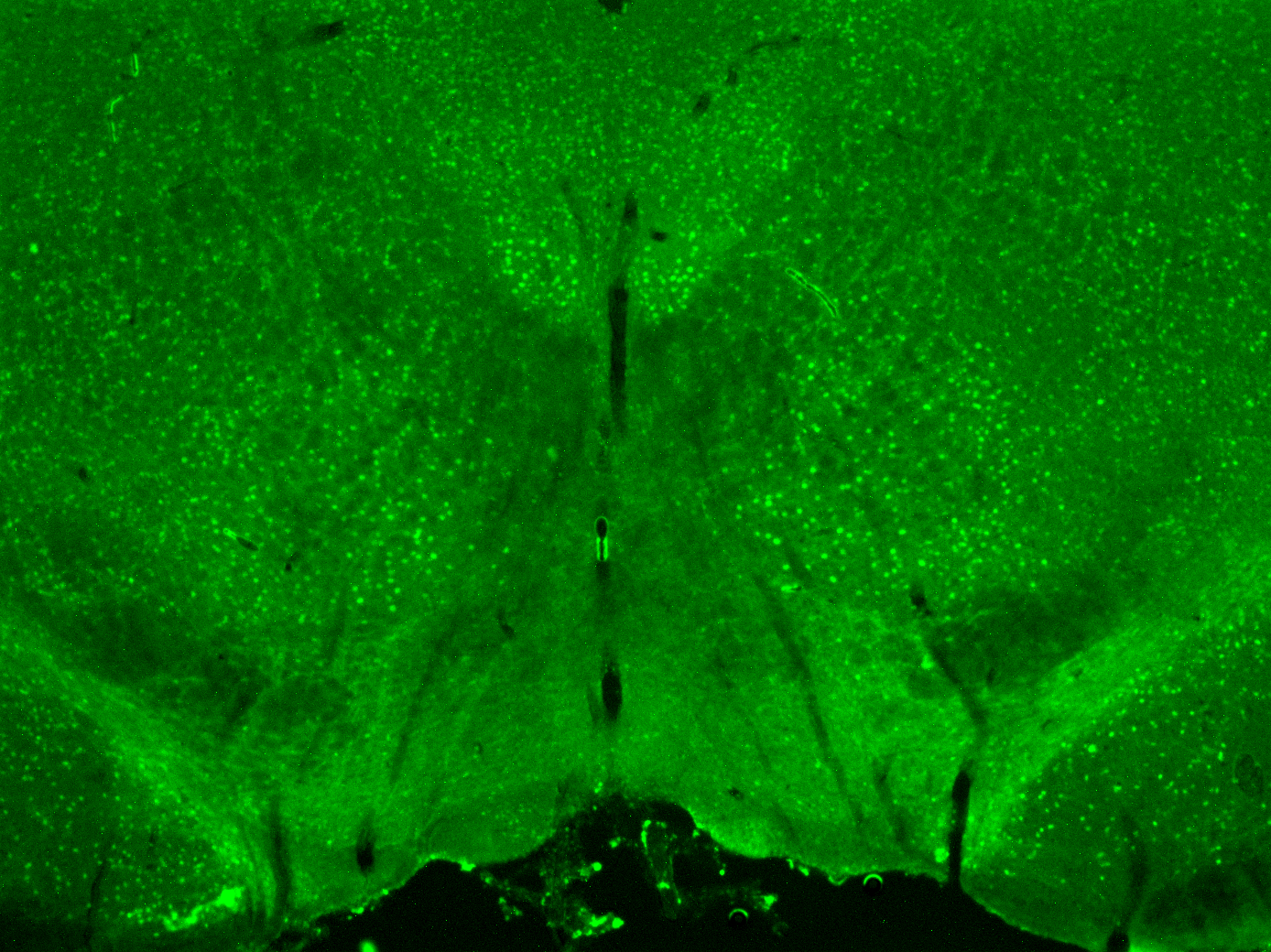

Supplement: Supplementary file 1 [file biomedicines-11-00820-s001.zip › Supplementary_Data_file_S2/DAT/PNG - DAT channel/SHAM-LHb100-DAT-488-VTA-2021-0001.zvi - C=1.png]

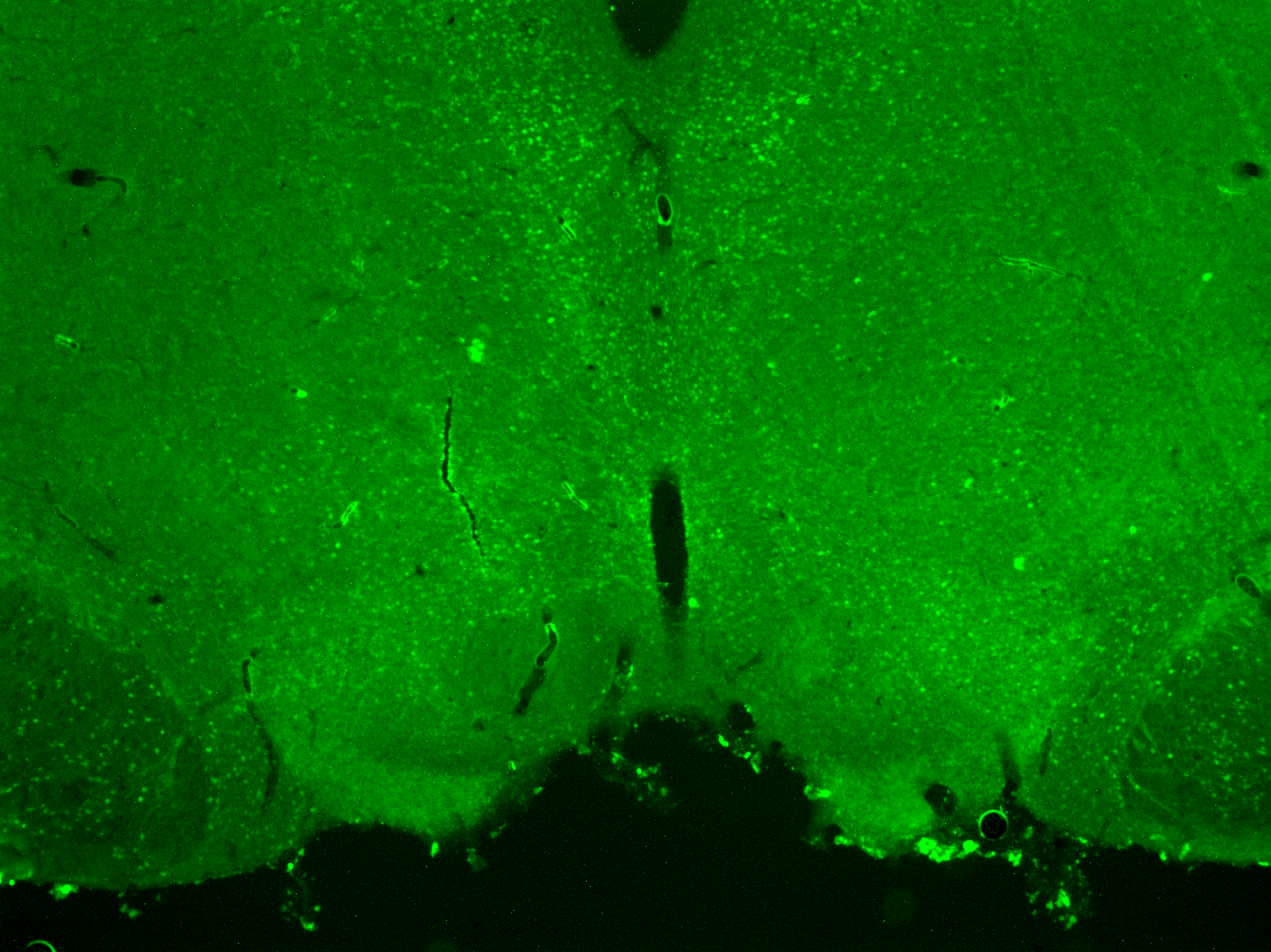

Supplement: Supplementary file 1 [file biomedicines-11-00820-s001.zip › Supplementary_Data_file_S2/DAT/PNG - DAT channel/SHAM-LHb101-DAT-488-VTA-2021-0003.zvi - C=1.png]

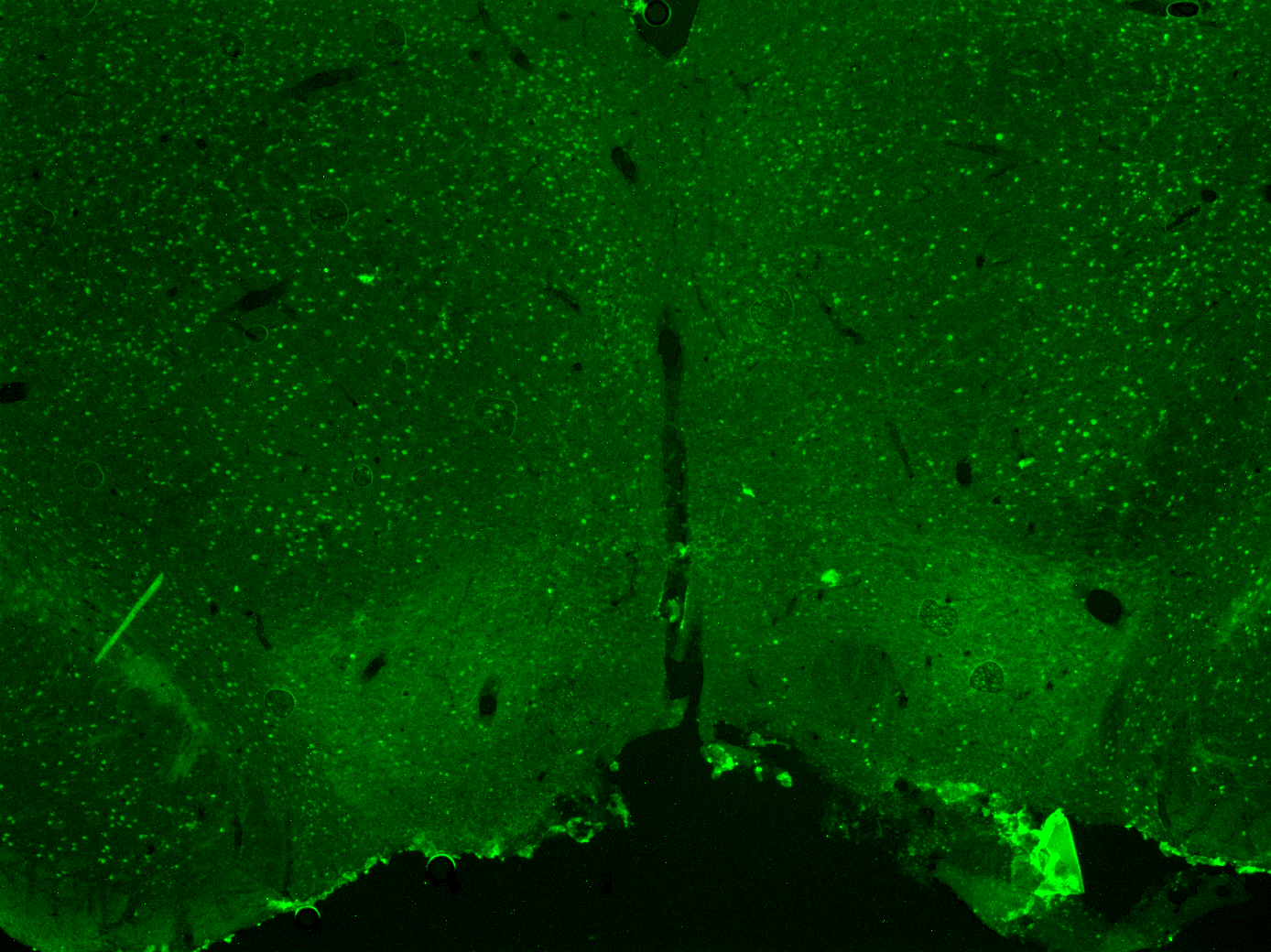

Supplement: Supplementary file 1 [file biomedicines-11-00820-s001.zip › Supplementary_Data_file_S2/DAT/PNG - DAT channel/SHAM-LHb102-DAT-488-VTA-2021-0004.zvi - C=1.png]

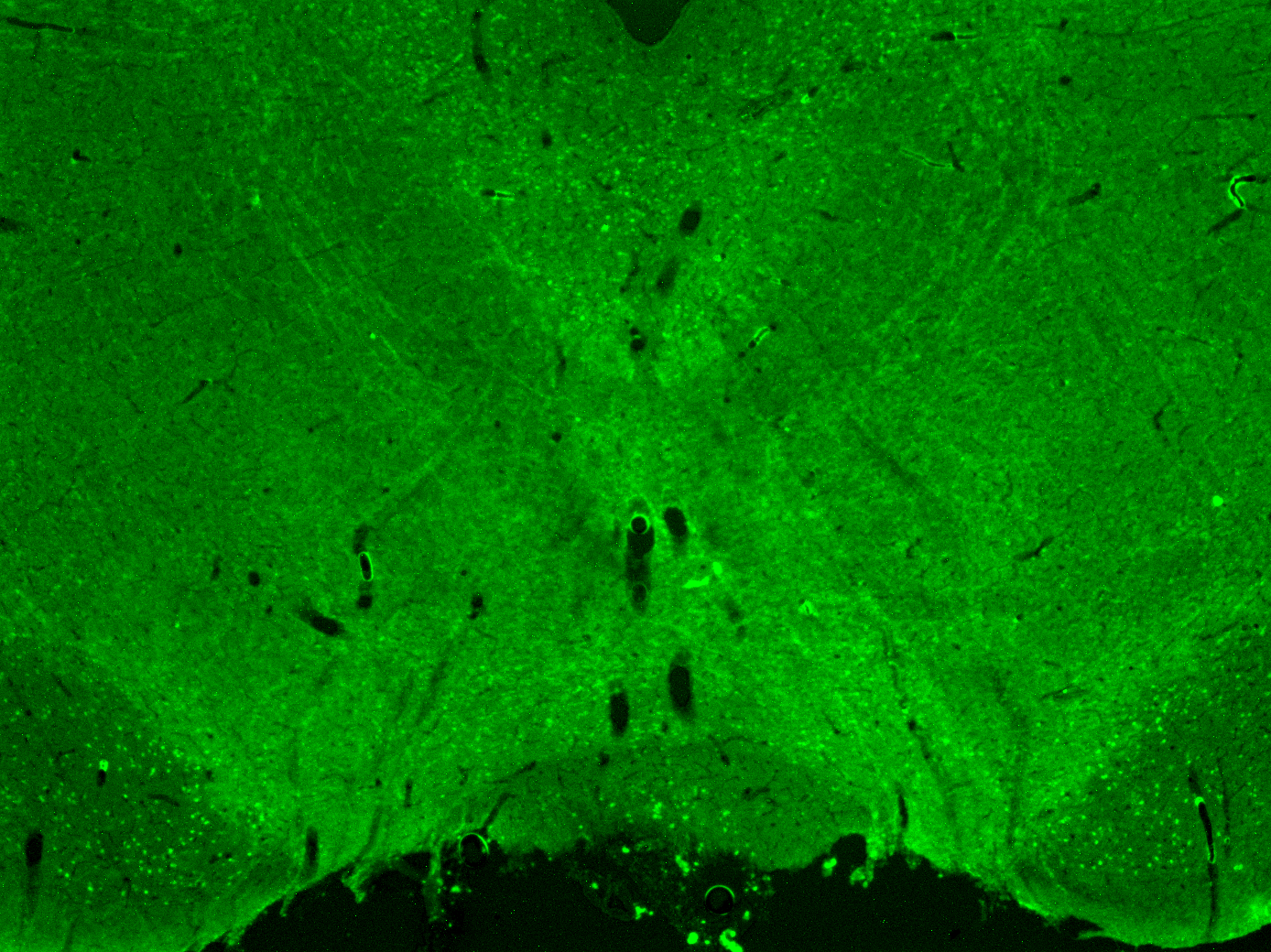

Supplement: Supplementary file 1 [file biomedicines-11-00820-s001.zip › Supplementary_Data_file_S2/DAT/PNG - DAT channel/SHAM-LHb103-DAT-488-VTA-2021-0005.zvi - C=1.png]

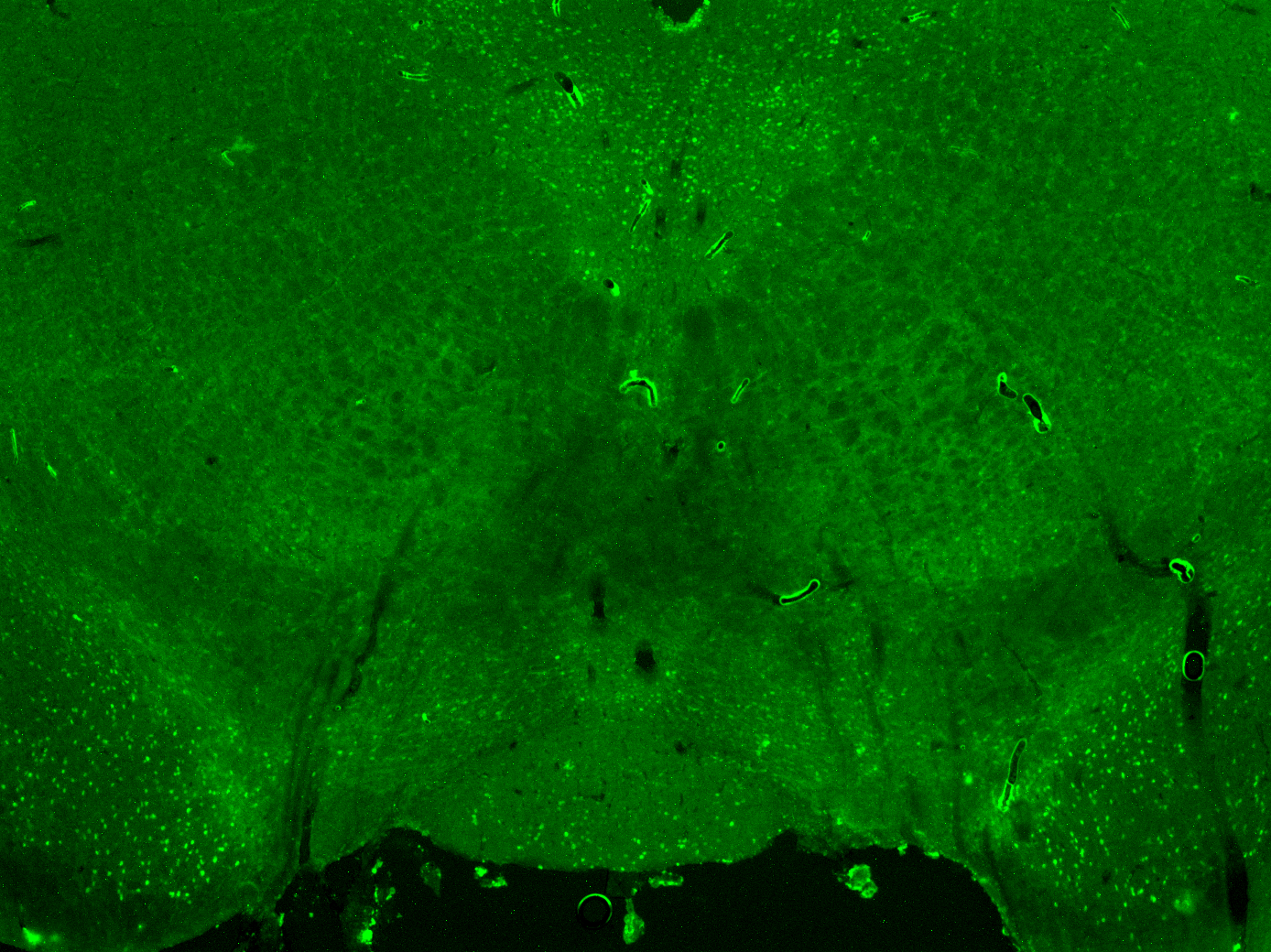

Supplement: Supplementary file 1 [file biomedicines-11-00820-s001.zip › Supplementary_Data_file_S2/DAT/PNG - DAT channel/SHAM-LHb111-DAT-488-VTA-2021-0013.zvi - C=1.png]

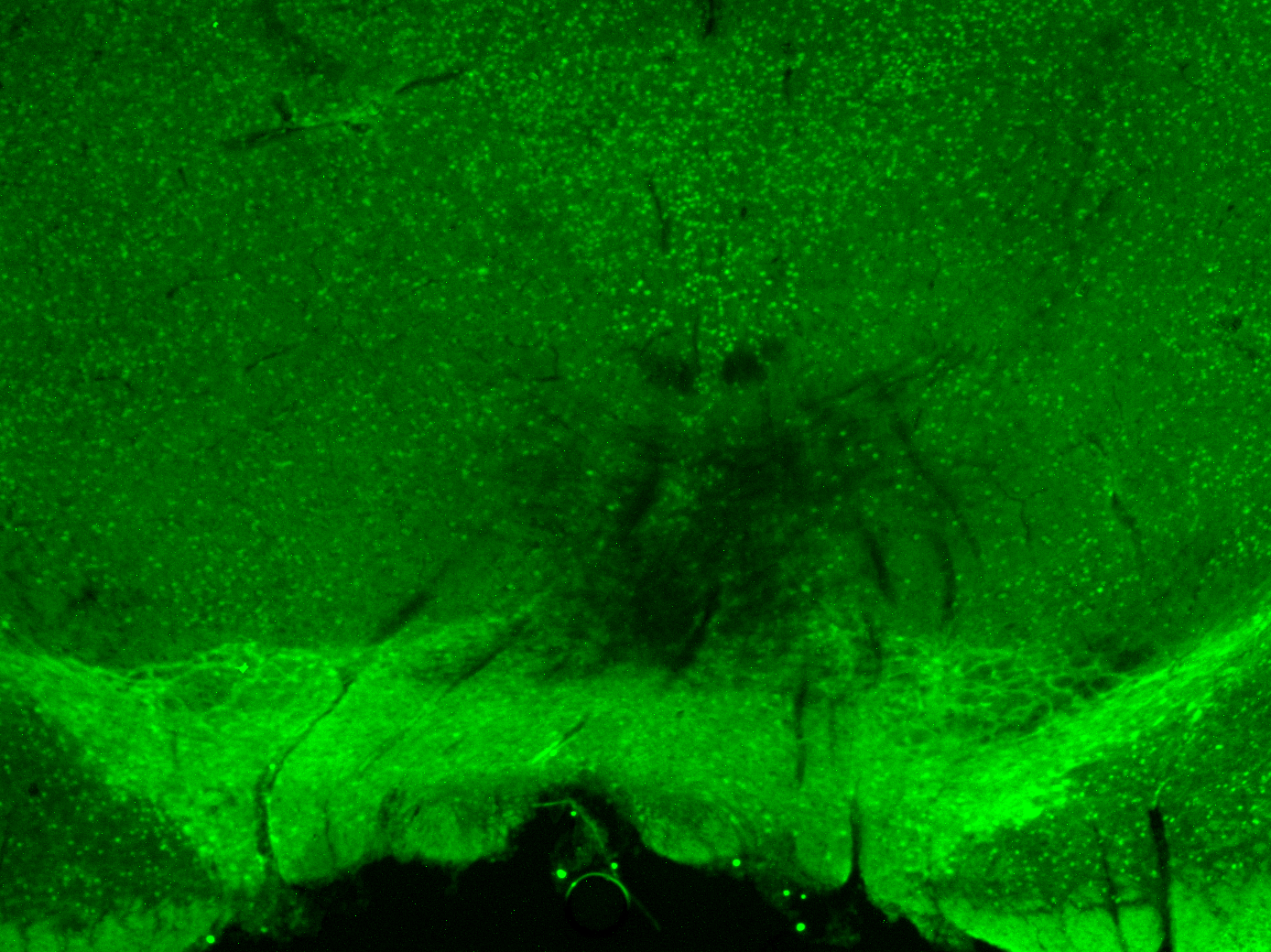

Supplement: Supplementary file 1 [file biomedicines-11-00820-s001.zip › Supplementary_Data_file_S2/DAT/PNG - DAT channel/SHAM-LHb120-DAT-488-VTA-2021-0022.zvi - C=1.png]

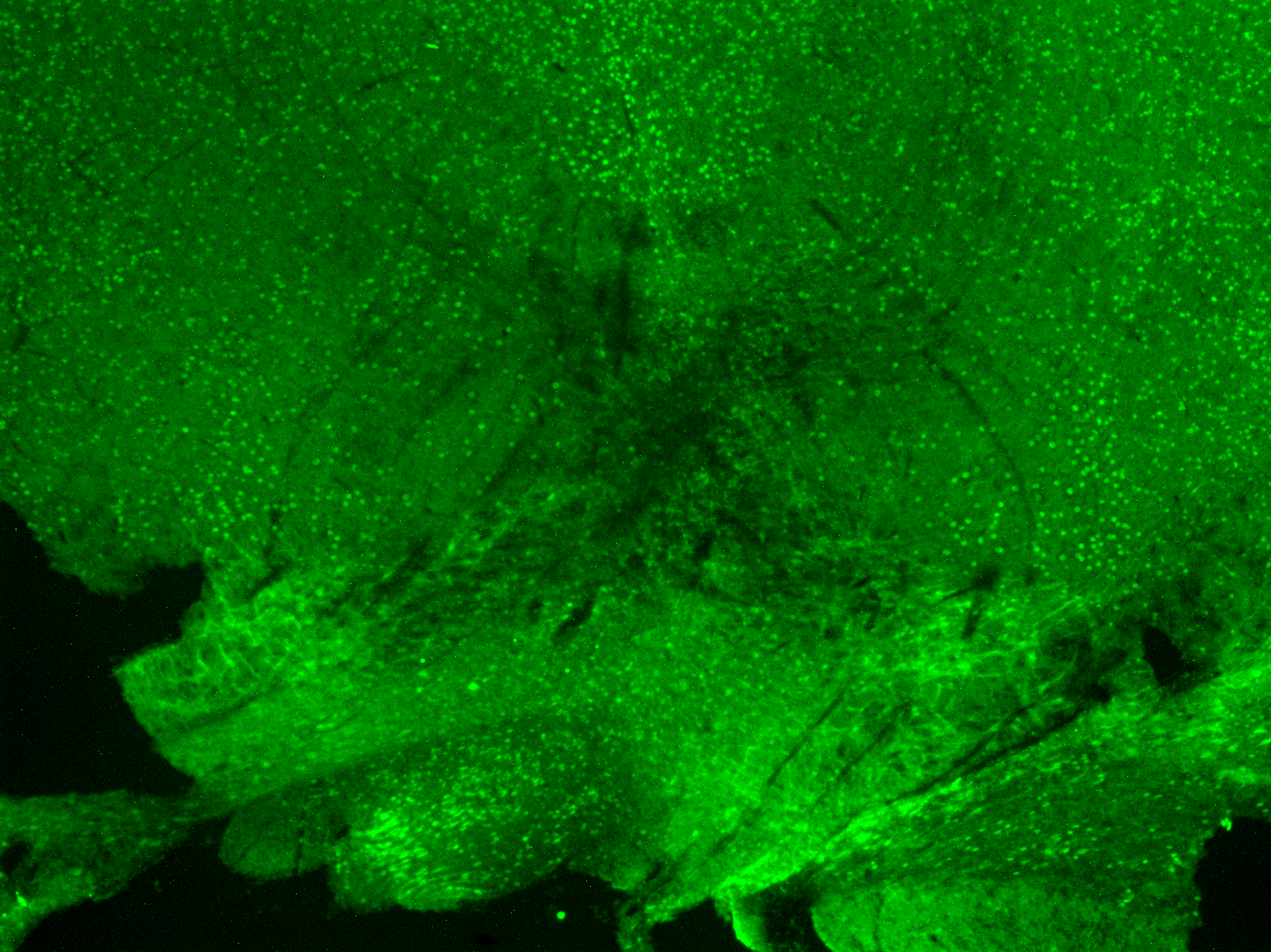

Supplement: Supplementary file 1 [file biomedicines-11-00820-s001.zip › Supplementary_Data_file_S2/DAT/PNG - DAT channel/SHAM-LHb121-DAT-488-VTA-2021-0024.zvi - C=1.png]

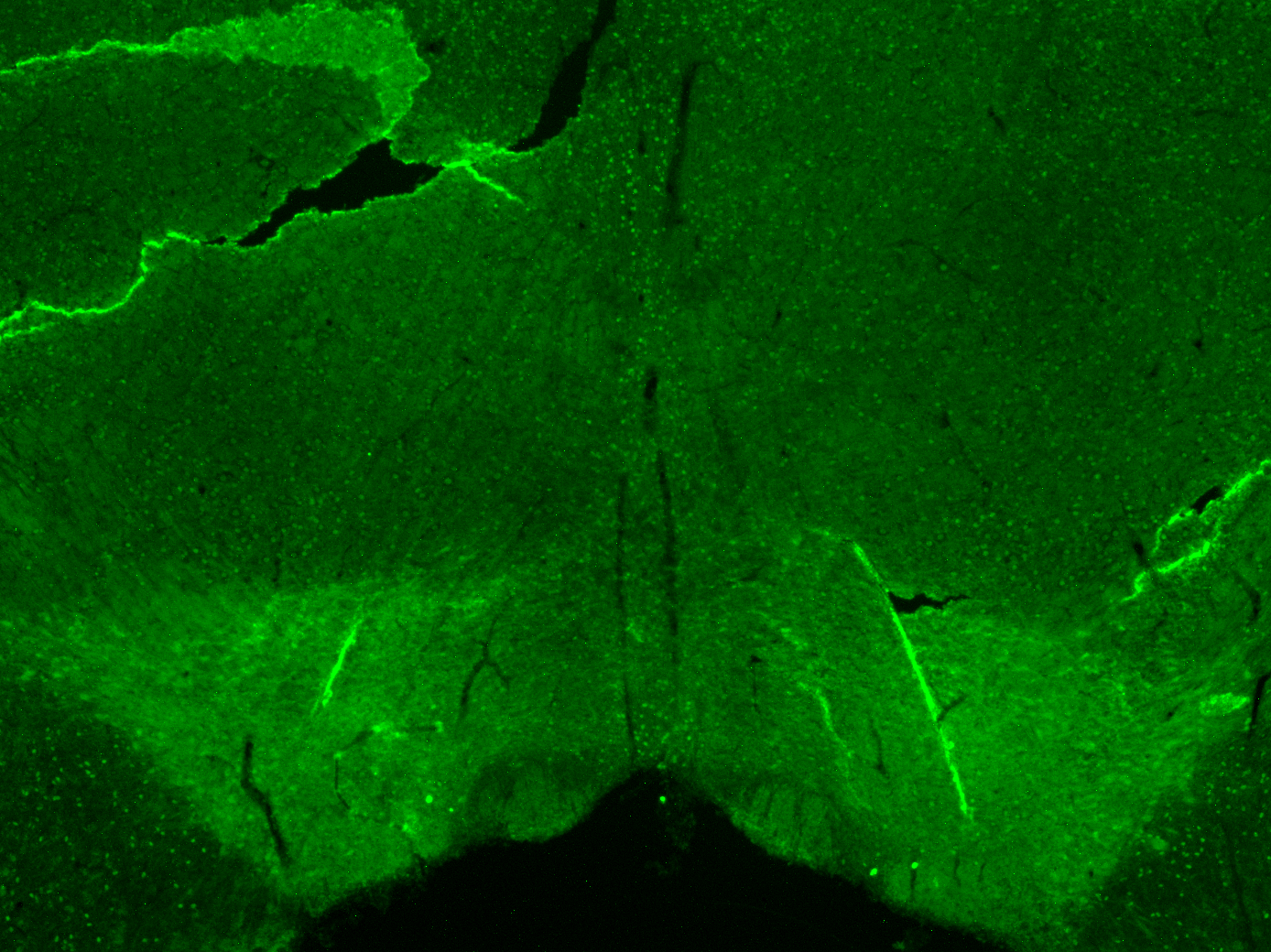

Supplement: Supplementary file 1 [file biomedicines-11-00820-s001.zip › Supplementary_Data_file_S2/DAT/PNG - DAT channel/SHAM-LHb122-DAT-488-VTA-2021-0025.zvi - C=1.png]

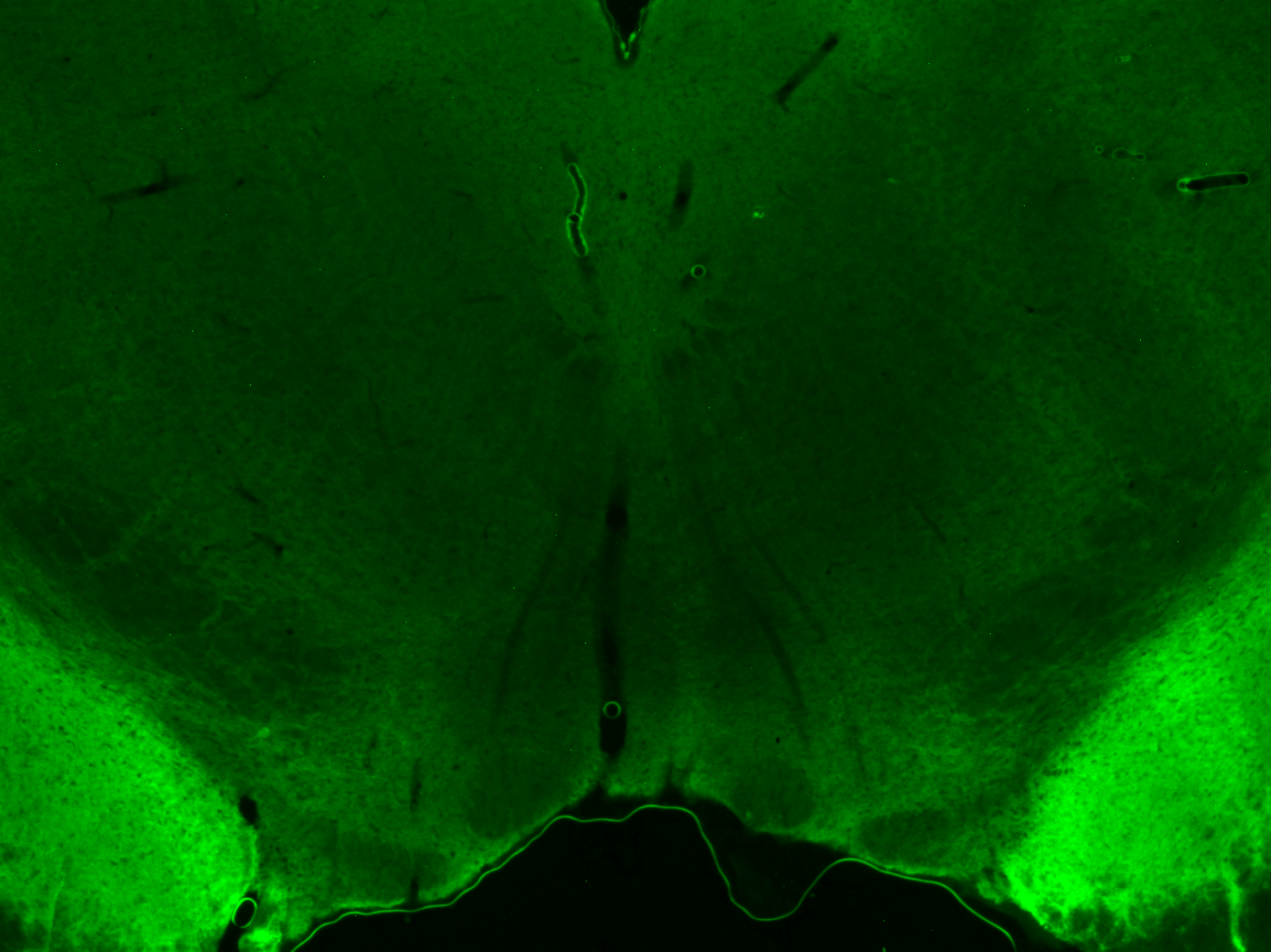

Supplement: Supplementary file 1 [file biomedicines-11-00820-s001.zip › Supplementary_Data_file_S2/GAD65_67/PNG - GAD channel/CFA-LHb104-GAD65-VTA-2021-0041.zvi - C=1.png]

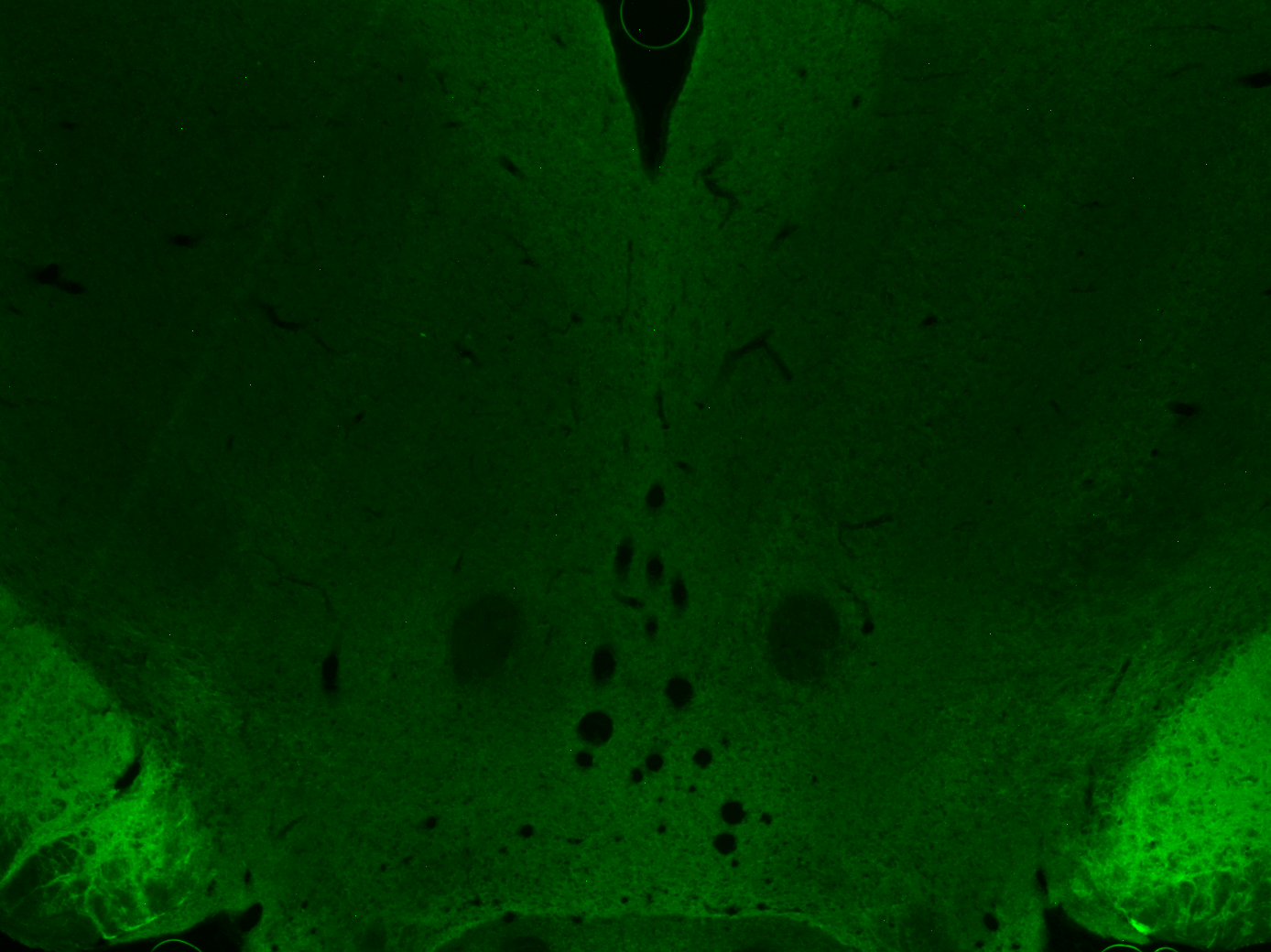

Supplement: Supplementary file 1 [file biomedicines-11-00820-s001.zip › Supplementary_Data_file_S2/GAD65_67/PNG - GAD channel/CFA-LHb105-GAD65-VTA-2021-0042.zvi - C=1.png]

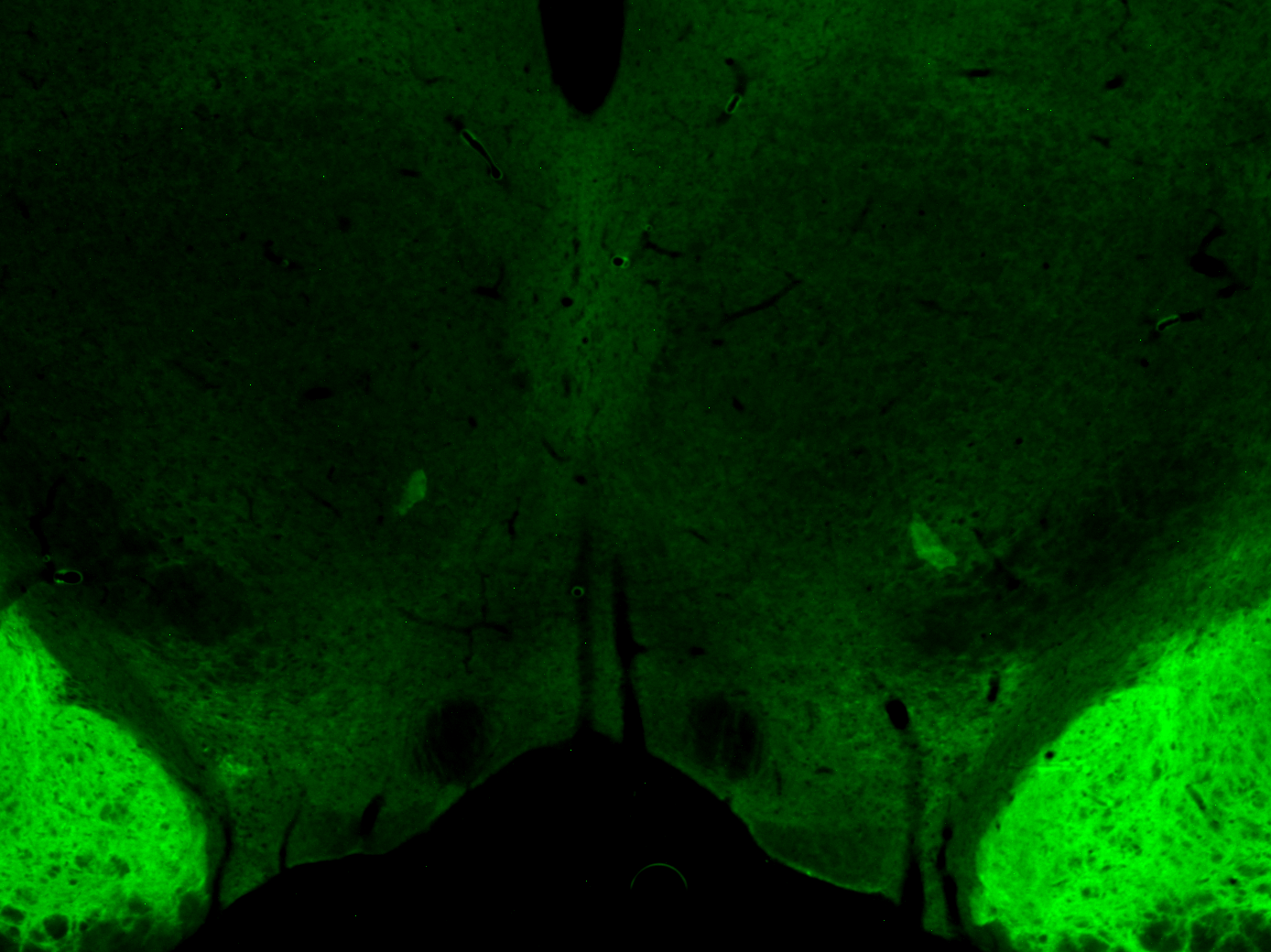

Supplement: Supplementary file 1 [file biomedicines-11-00820-s001.zip › Supplementary_Data_file_S2/GAD65_67/PNG - GAD channel/CFA-LHb106-GAD65-VTA-2021-0043.zvi - C=1.png]

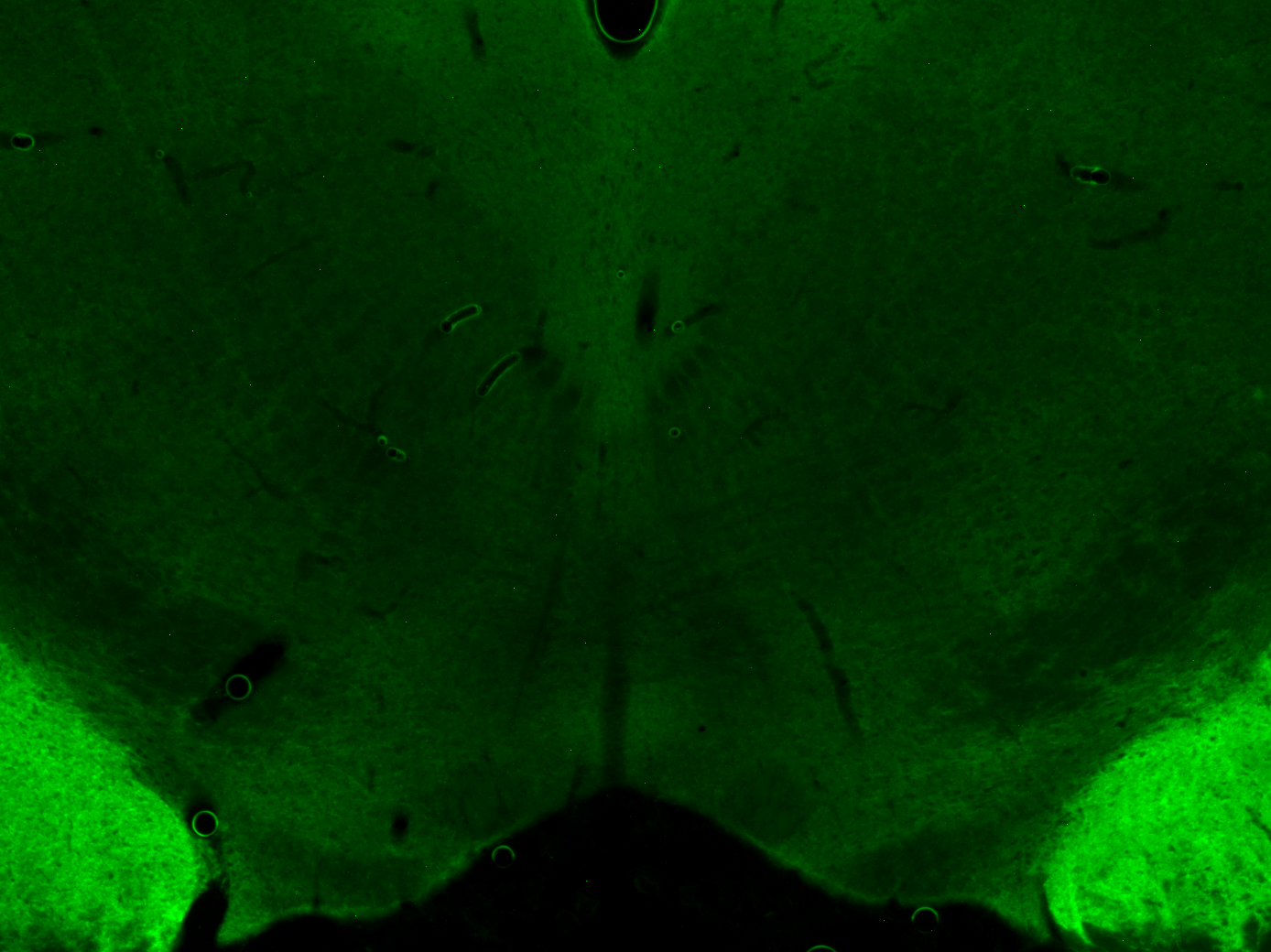

Supplement: Supplementary file 1 [file biomedicines-11-00820-s001.zip › Supplementary_Data_file_S2/GAD65_67/PNG - GAD channel/CFA-LHb107-GAD65-VTA-2021-0044.zvi - C=1.png]

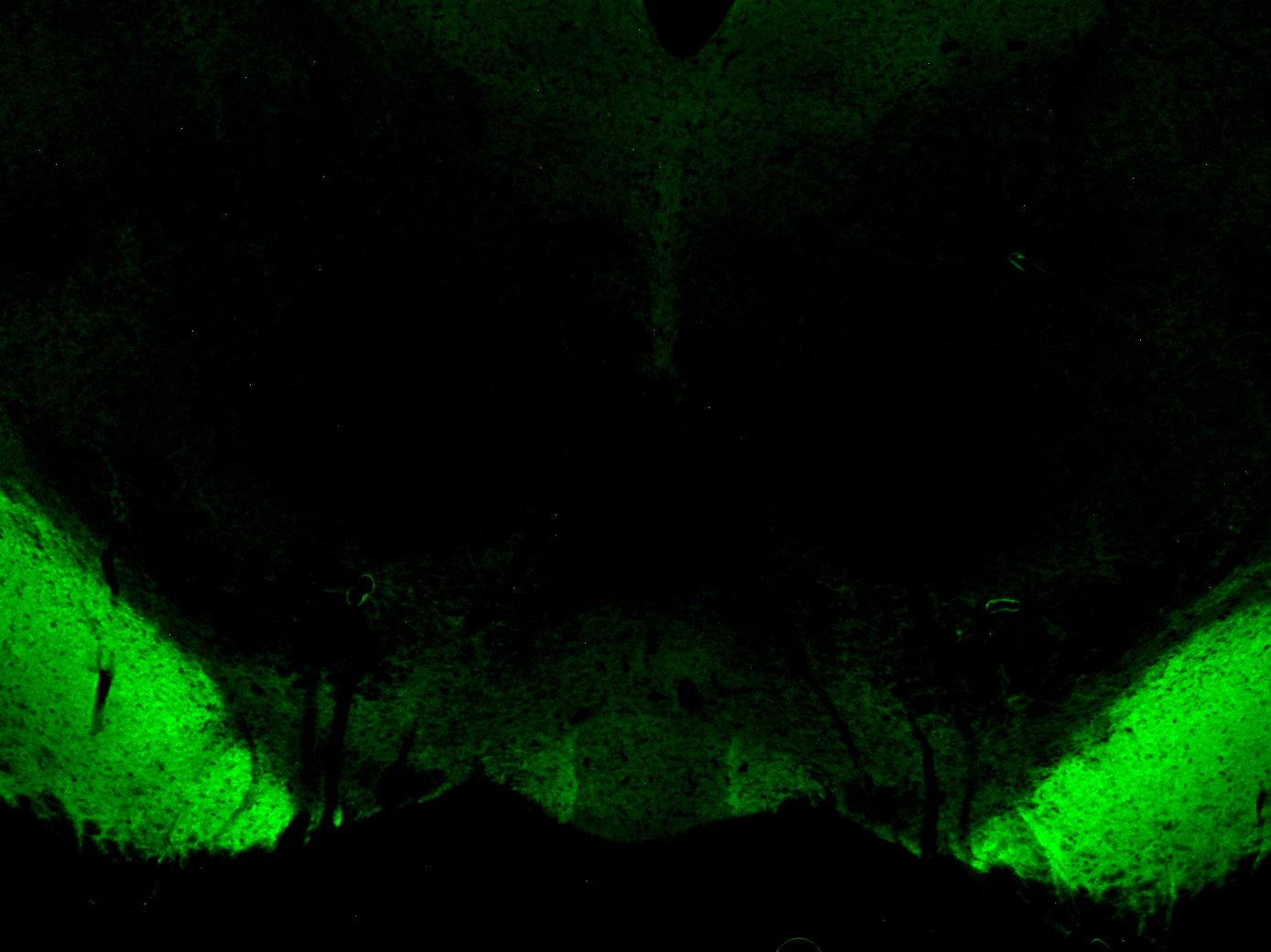

Supplement: Supplementary file 1 [file biomedicines-11-00820-s001.zip › Supplementary_Data_file_S2/GAD65_67/PNG - GAD channel/CFA-LHb108-GAD65-VTA-2021-0045.zvi - C=1.png]

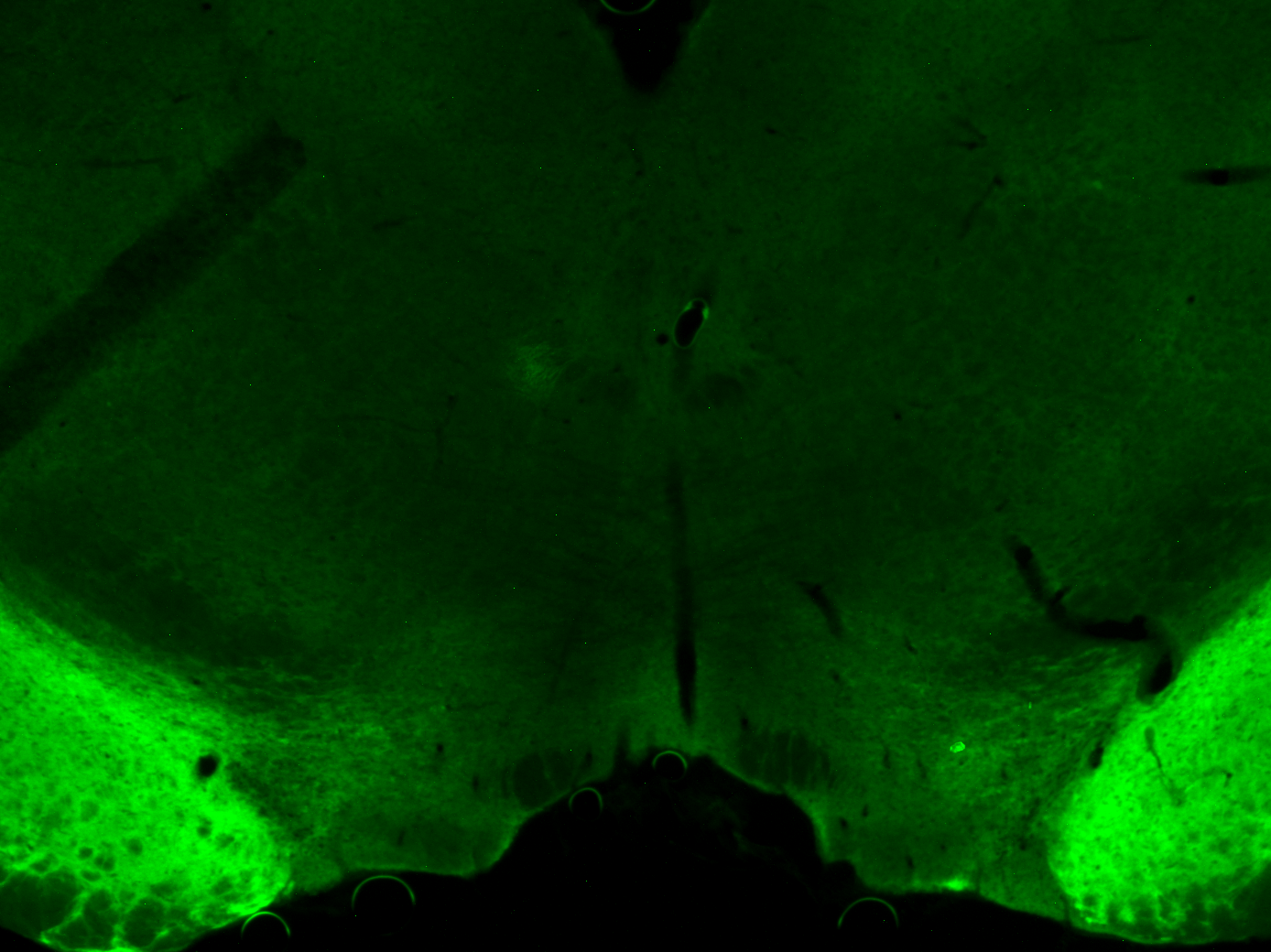

Supplement: Supplementary file 1 [file biomedicines-11-00820-s001.zip › Supplementary_Data_file_S2/GAD65_67/PNG - GAD channel/CFA-LHb109-GAD65-VTA-2021-0046.zvi - C=1.png]

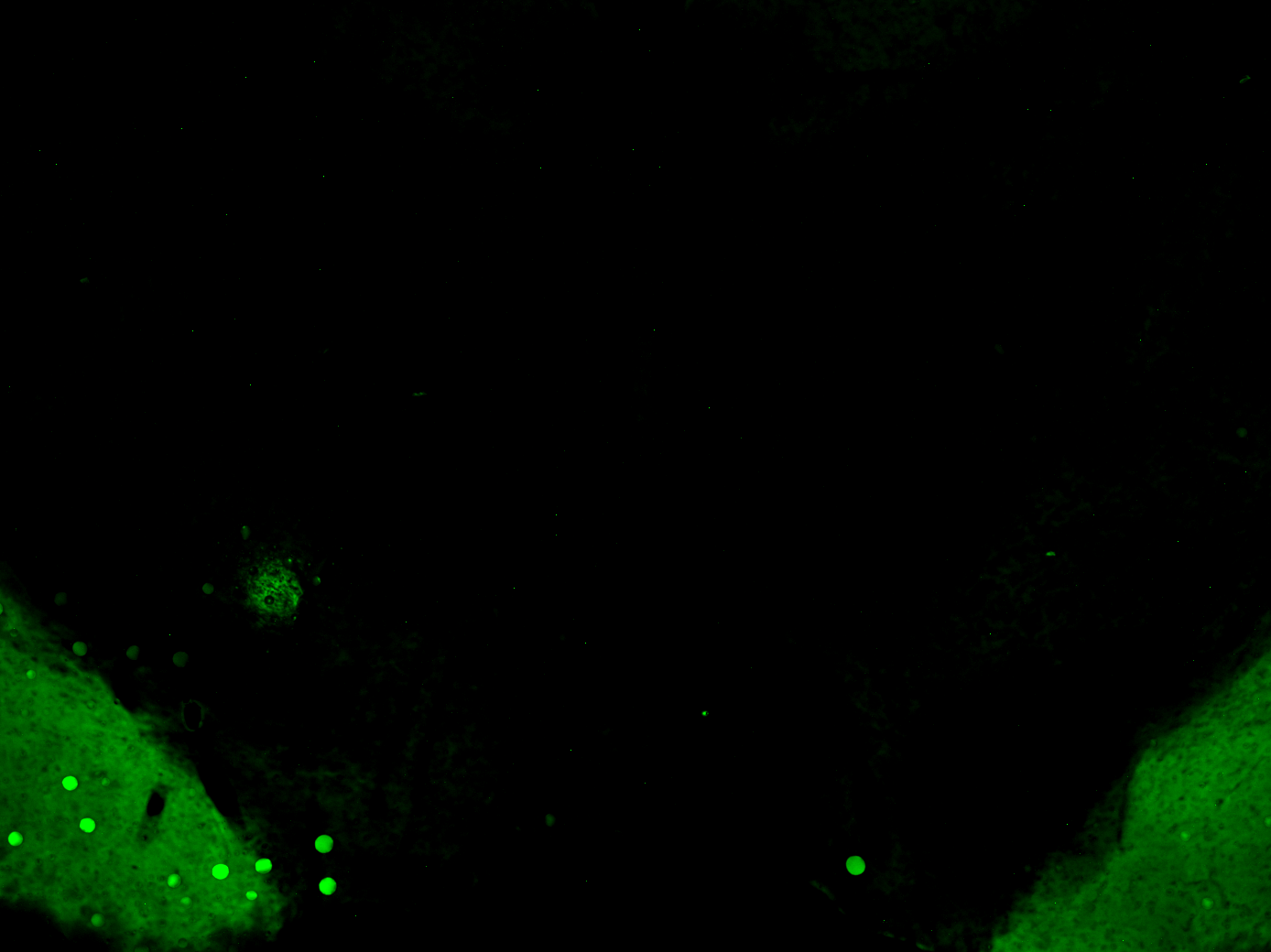

Supplement: Supplementary file 1 [file biomedicines-11-00820-s001.zip › Supplementary_Data_file_S2/GAD65_67/PNG - GAD channel/SHAM-LHb100-GAD65-VTA-2021-0037.zvi - C=1.png]

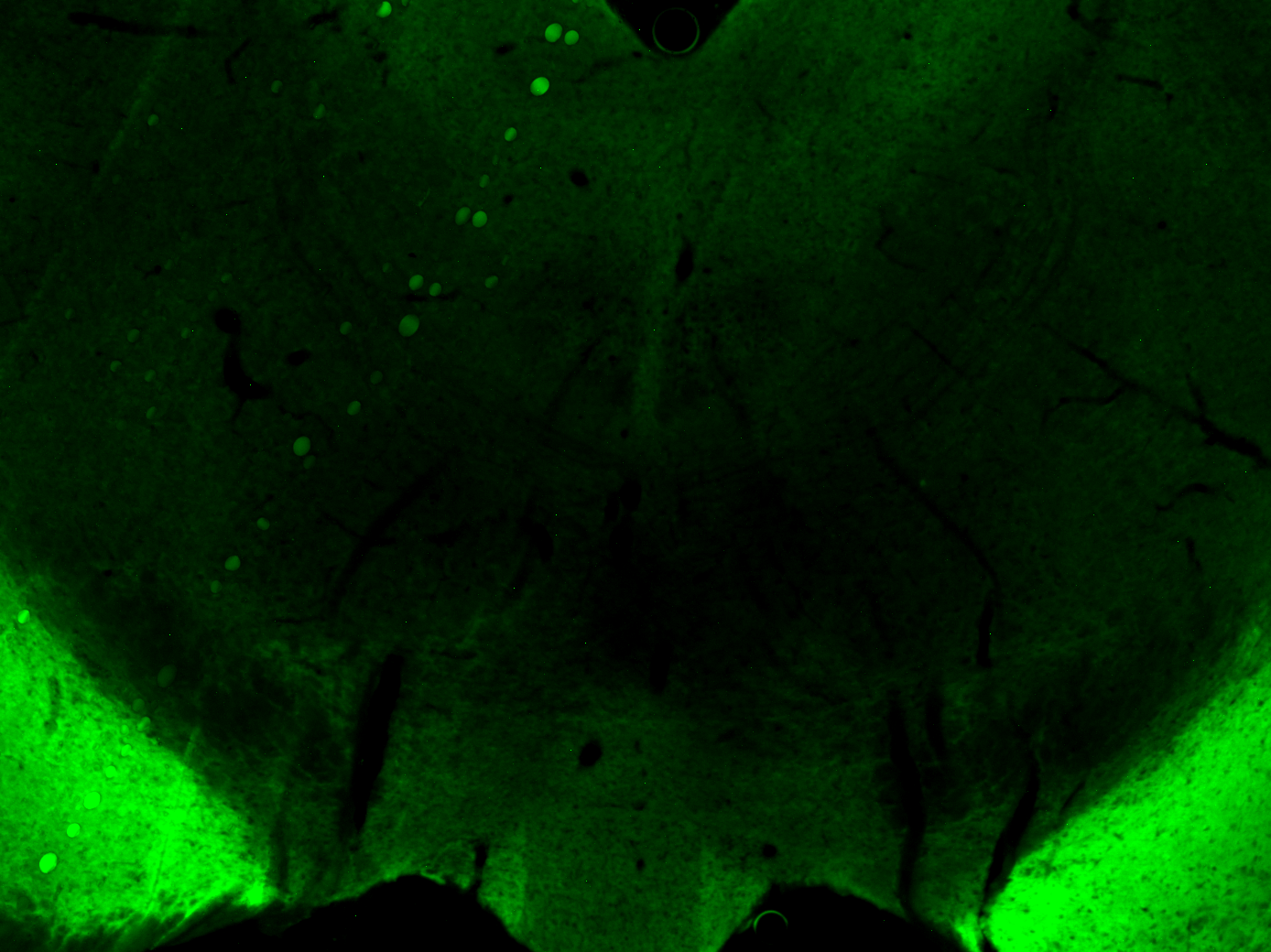

Supplement: Supplementary file 1 [file biomedicines-11-00820-s001.zip › Supplementary_Data_file_S2/GAD65_67/PNG - GAD channel/SHAM-LHb101-GAD65-VTA-2021-0038.zvi - C=1.png]

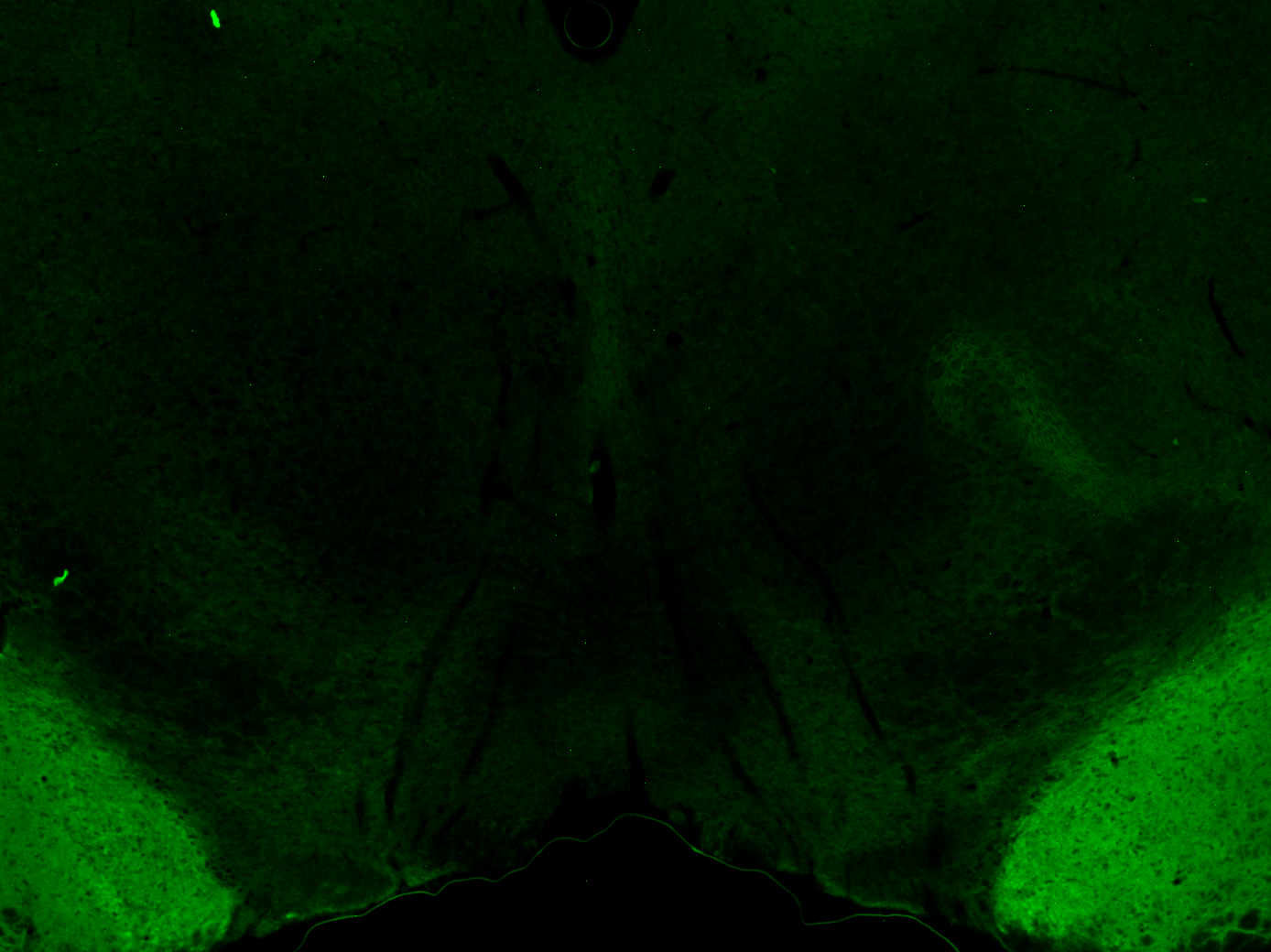

Supplement: Supplementary file 1 [file biomedicines-11-00820-s001.zip › Supplementary_Data_file_S2/GAD65_67/PNG - GAD channel/SHAM-LHb102-GAD65-VTA-2021-0039.zvi - C=1.png]

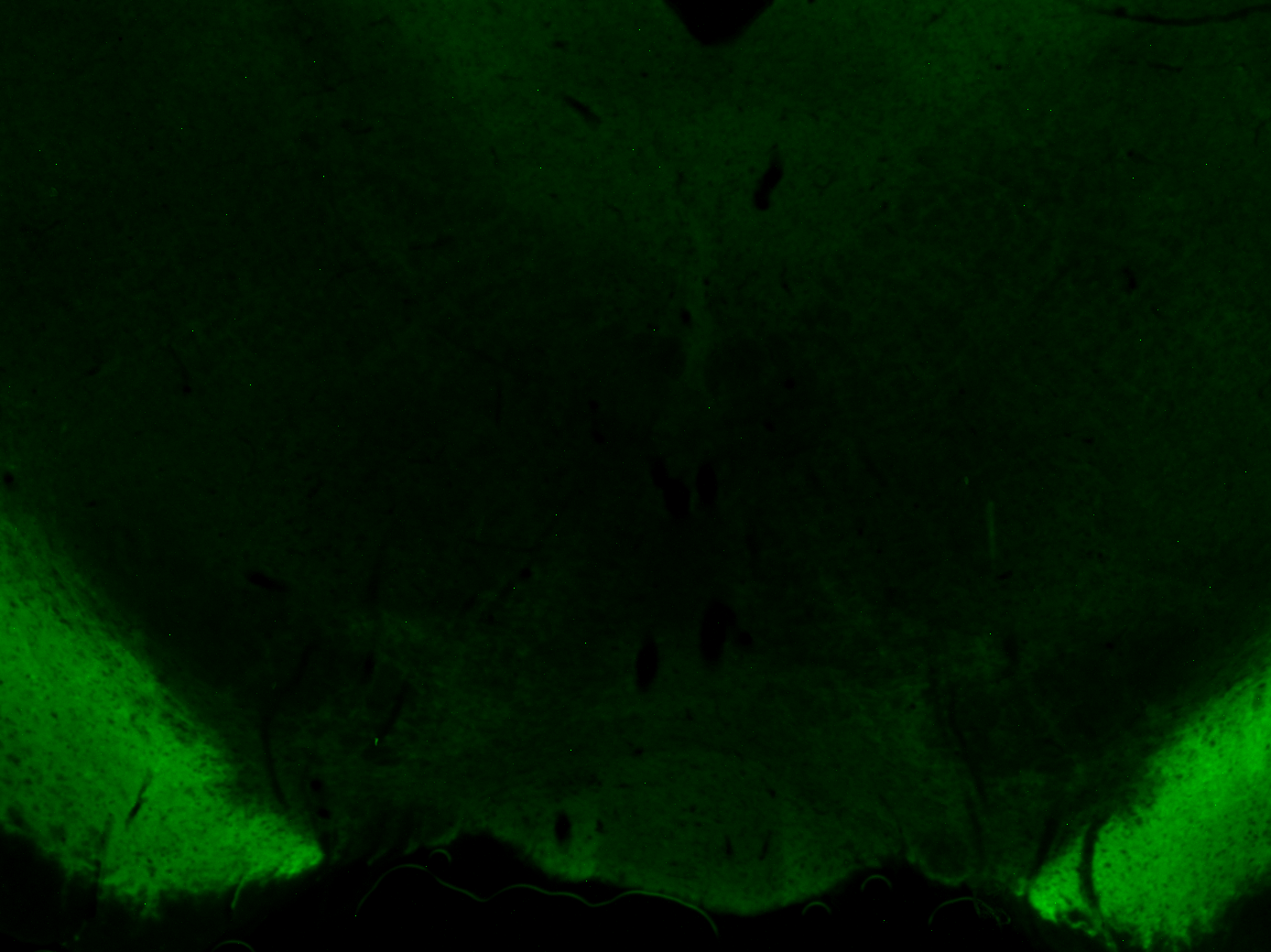

Supplement: Supplementary file 1 [file biomedicines-11-00820-s001.zip › Supplementary_Data_file_S2/GAD65_67/PNG - GAD channel/SHAM-LHb103-GAD65-VTA-2021-0040.zvi - C=1.png]

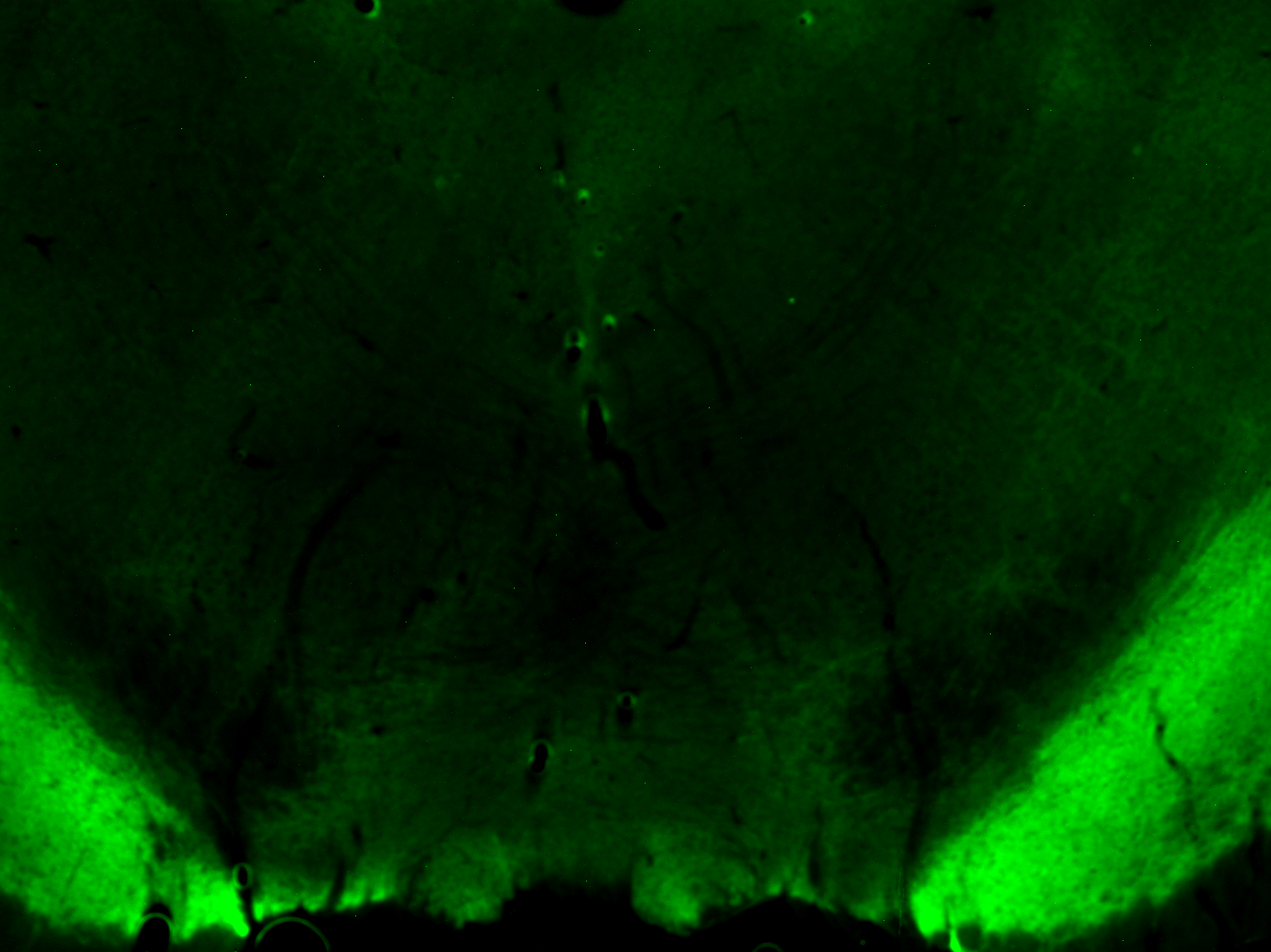

Supplement: Supplementary file 1 [file biomedicines-11-00820-s001.zip › Supplementary_Data_file_S2/GAD65_67/PNG - GAD channel/SHAM-LHb110-GAD65-VTA-2021-0047.zvi - C=1.png]

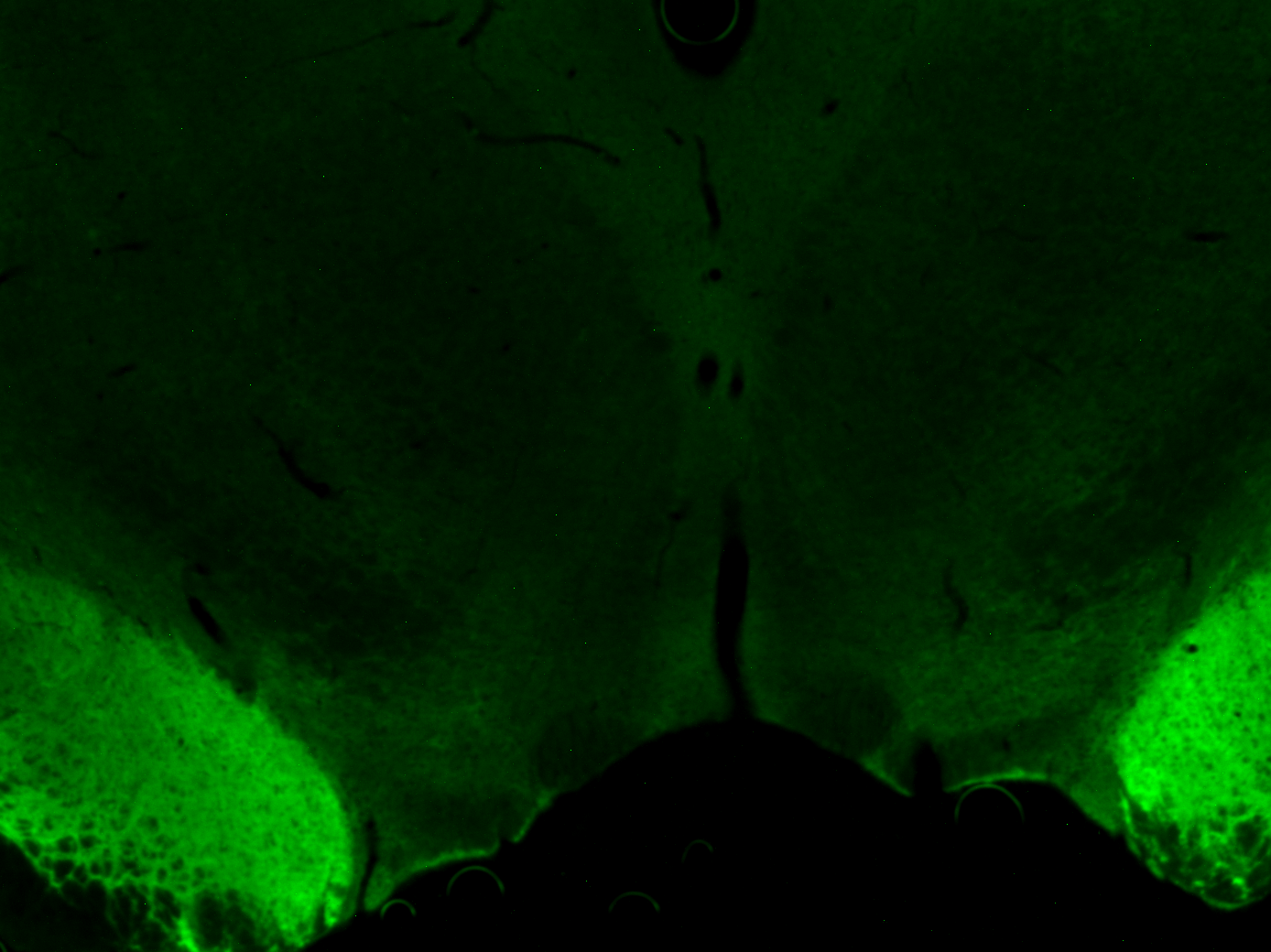

Supplement: Supplementary file 1 [file biomedicines-11-00820-s001.zip › Supplementary_Data_file_S2/GAD65_67/PNG - GAD channel/SHAM-LHb111-GAD65-VTA-2021-0048.zvi - C=1.png]

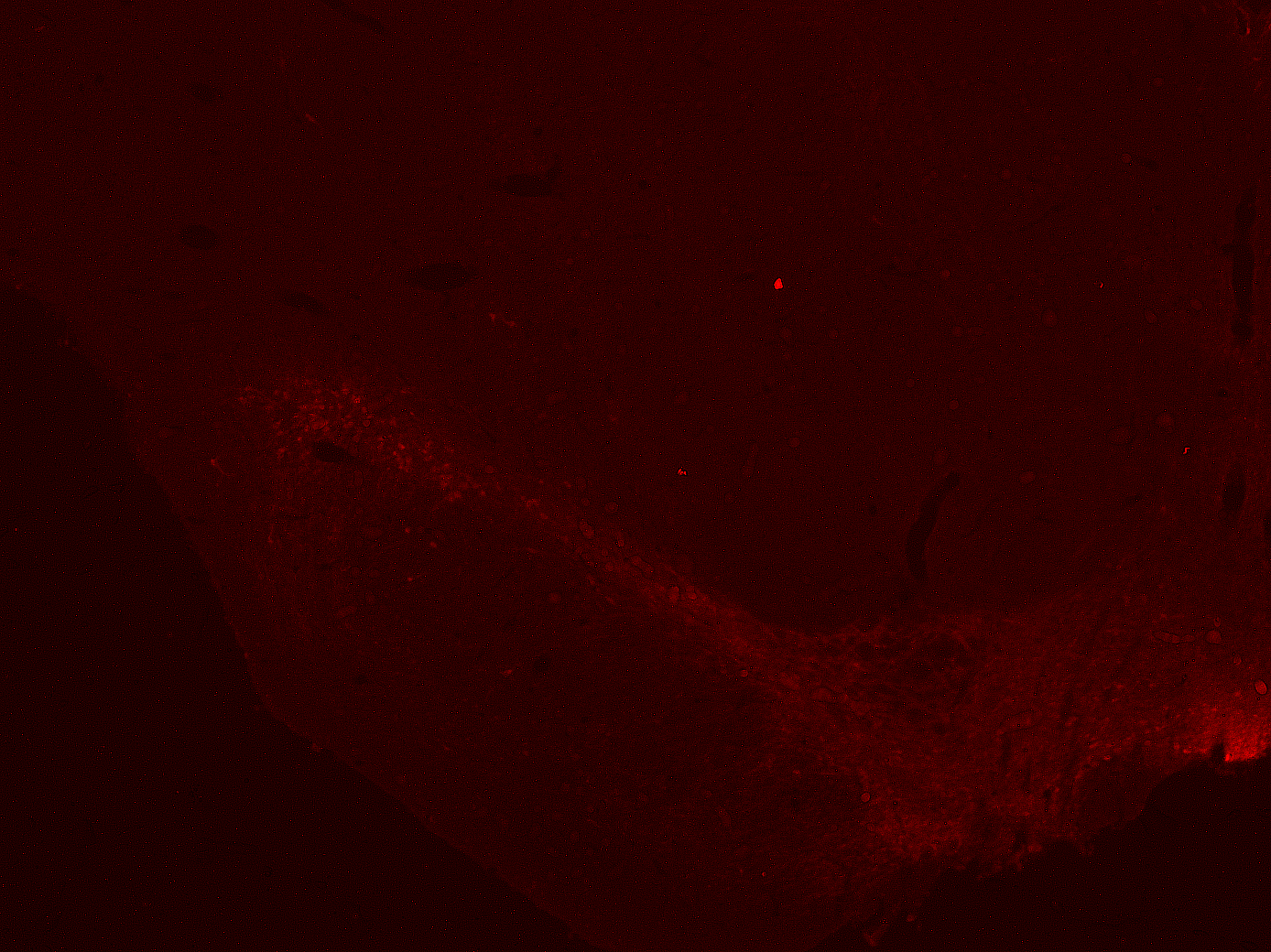

Supplement: Supplementary file 1 [file biomedicines-11-00820-s001.zip › Supplementary_Data_file_S2/TH/PNG - TH channel/CFA-LHb104-TH-568-VTA-2021-0026-Left.zvi - C=1.png]

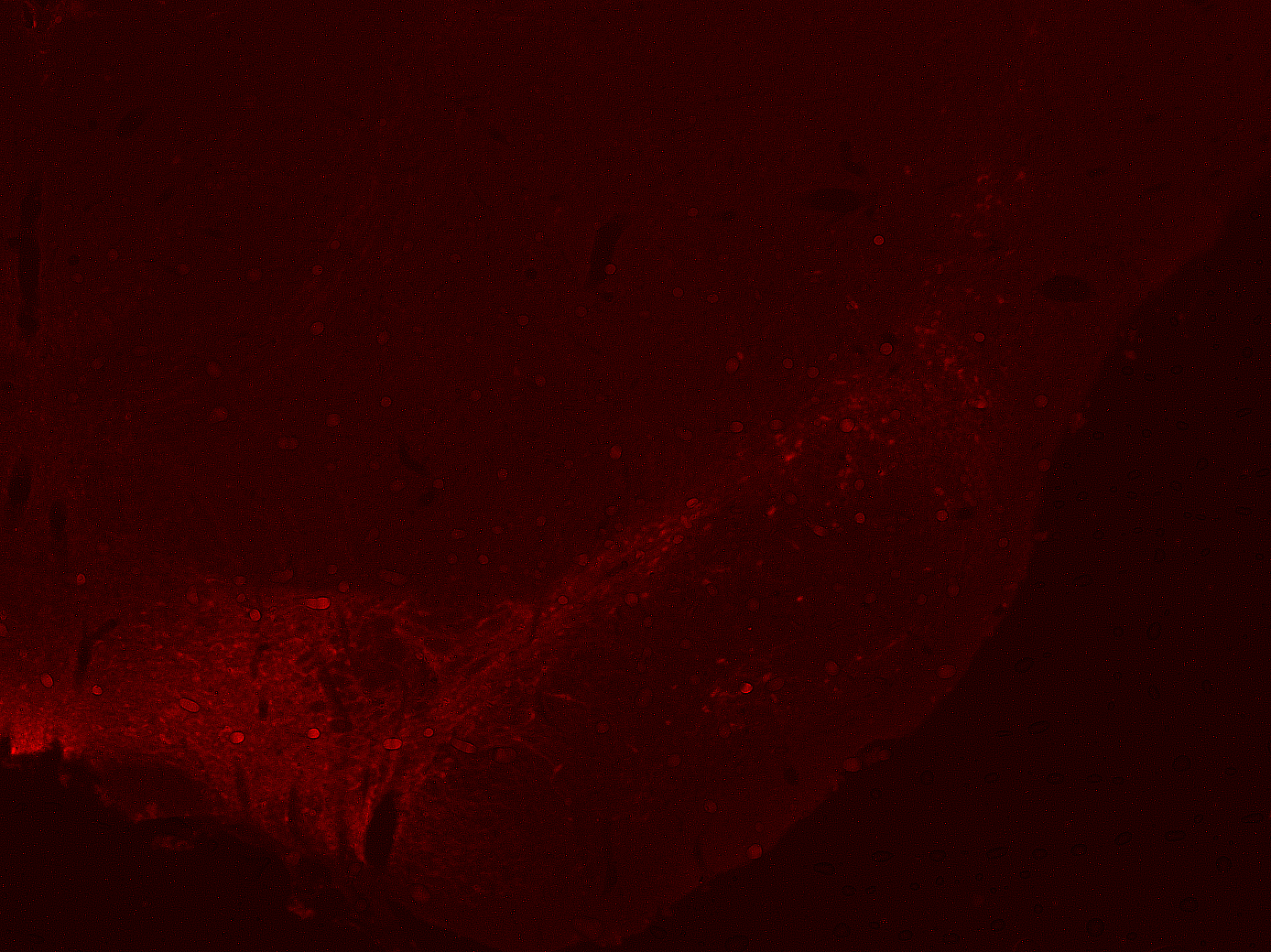

Supplement: Supplementary file 1 [file biomedicines-11-00820-s001.zip › Supplementary_Data_file_S2/TH/PNG - TH channel/CFA-LHb104-TH-568-VTA-2021-0027-Right.zvi - C=1.png]

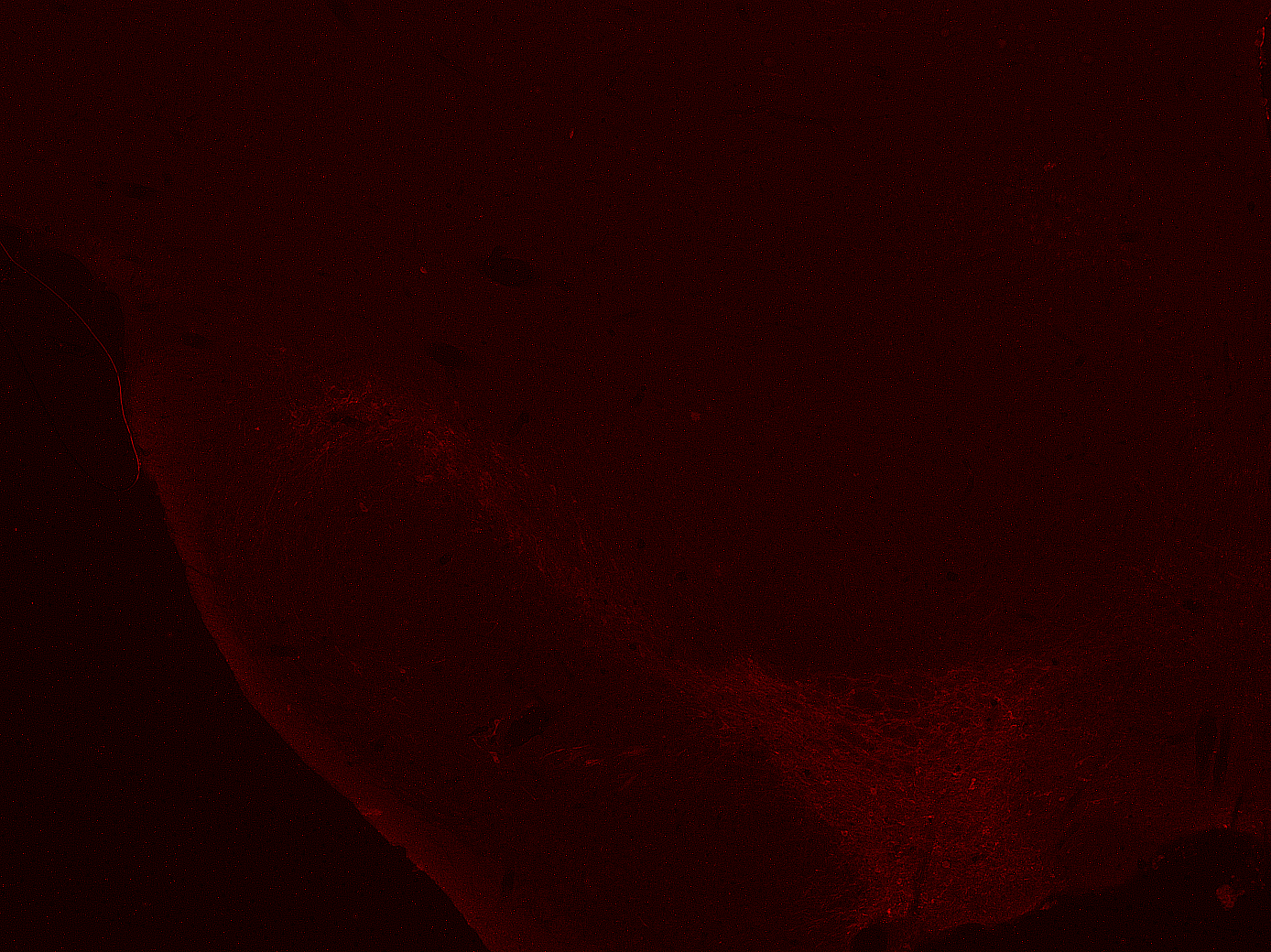

Supplement: Supplementary file 1 [file biomedicines-11-00820-s001.zip › Supplementary_Data_file_S2/TH/PNG - TH channel/CFA-LHb106-TH-568-VTA-2021-0028-Left.zvi - C=1.png]

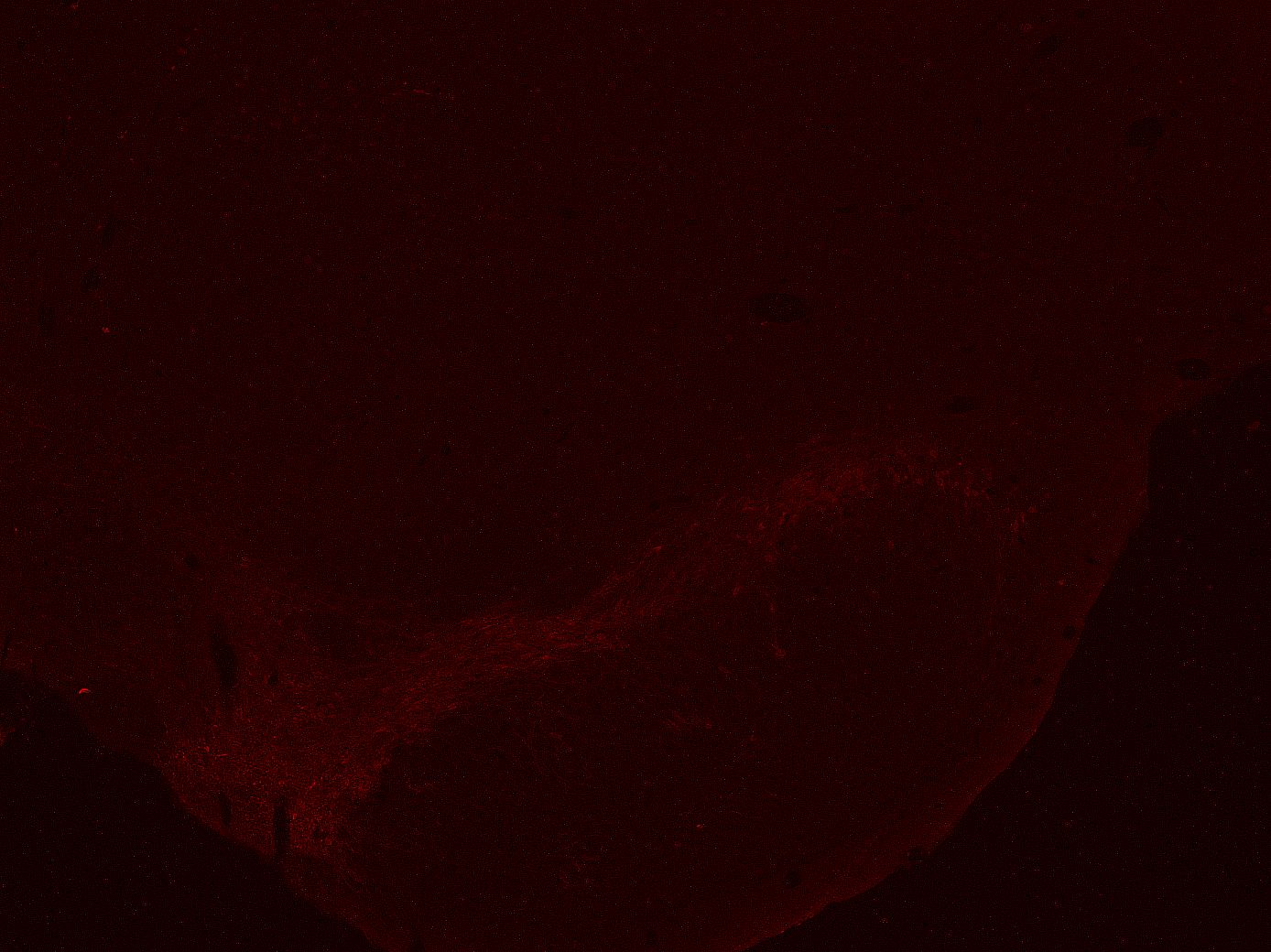

Supplement: Supplementary file 1 [file biomedicines-11-00820-s001.zip › Supplementary_Data_file_S2/TH/PNG - TH channel/CFA-LHb106-TH-568-VTA-2021-0029-Right.zvi - C=1.png]

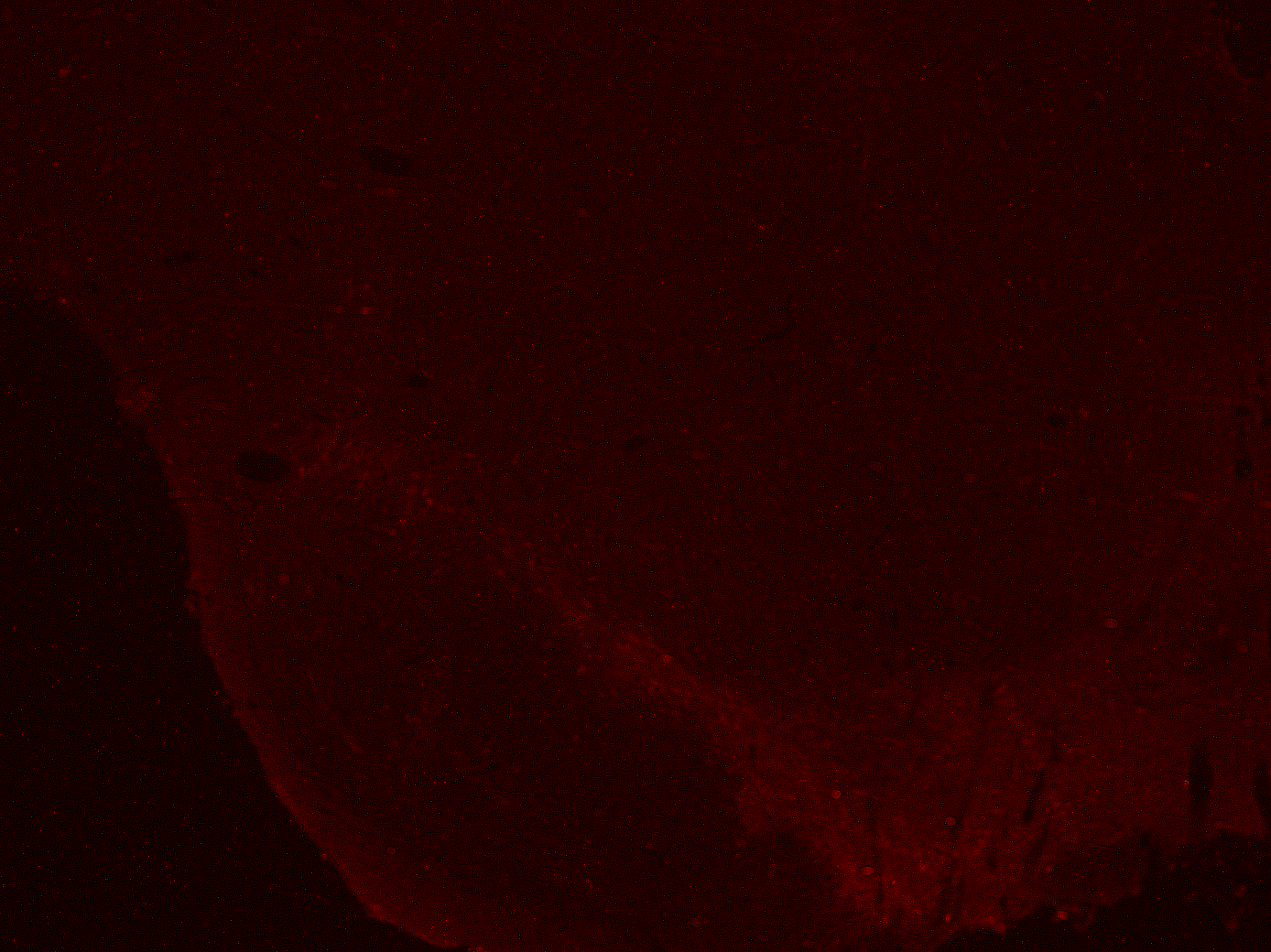

Supplement: Supplementary file 1 [file biomedicines-11-00820-s001.zip › Supplementary_Data_file_S2/TH/PNG - TH channel/CFA-LHb107-TH-568-VTA-2021-0030-Left.zvi - C=1.png]

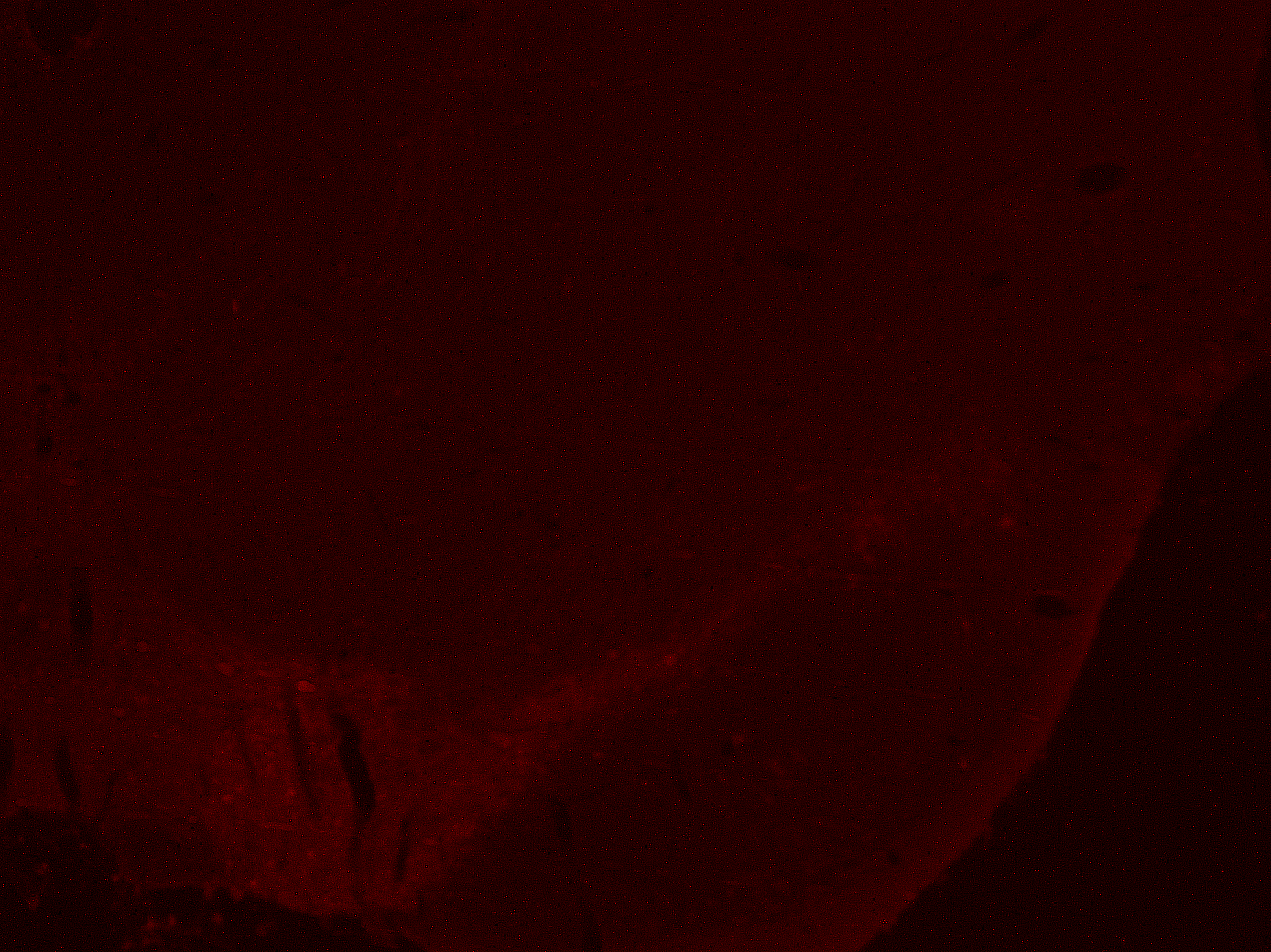

Supplement: Supplementary file 1 [file biomedicines-11-00820-s001.zip › Supplementary_Data_file_S2/TH/PNG - TH channel/CFA-LHb107-TH-568-VTA-2021-0031-Right.zvi - C=1.png]

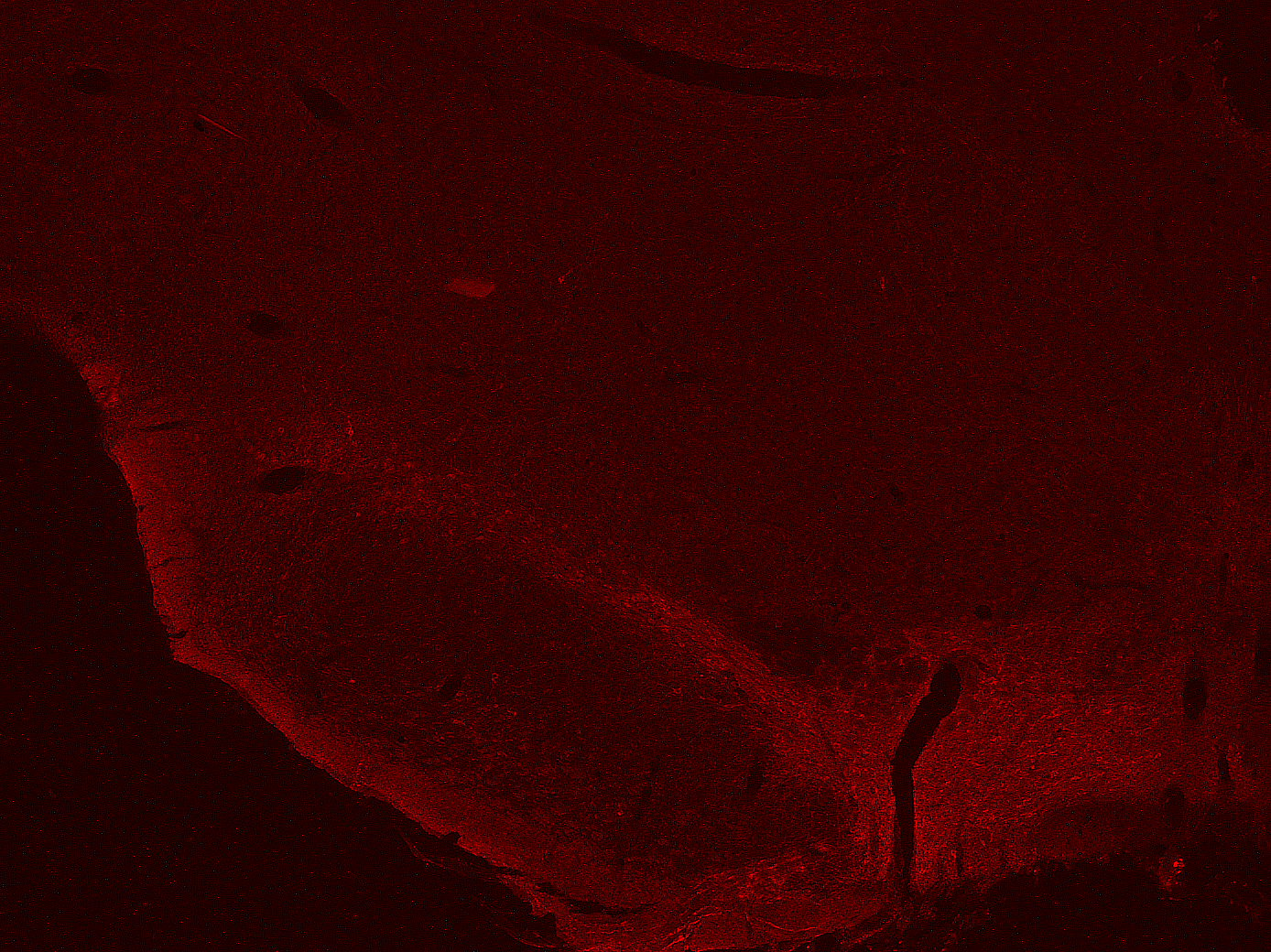

Supplement: Supplementary file 1 [file biomedicines-11-00820-s001.zip › Supplementary_Data_file_S2/TH/PNG - TH channel/CFA-LHb108-TH-568-VTA-2021-0033-Left.zvi - C=1.png]

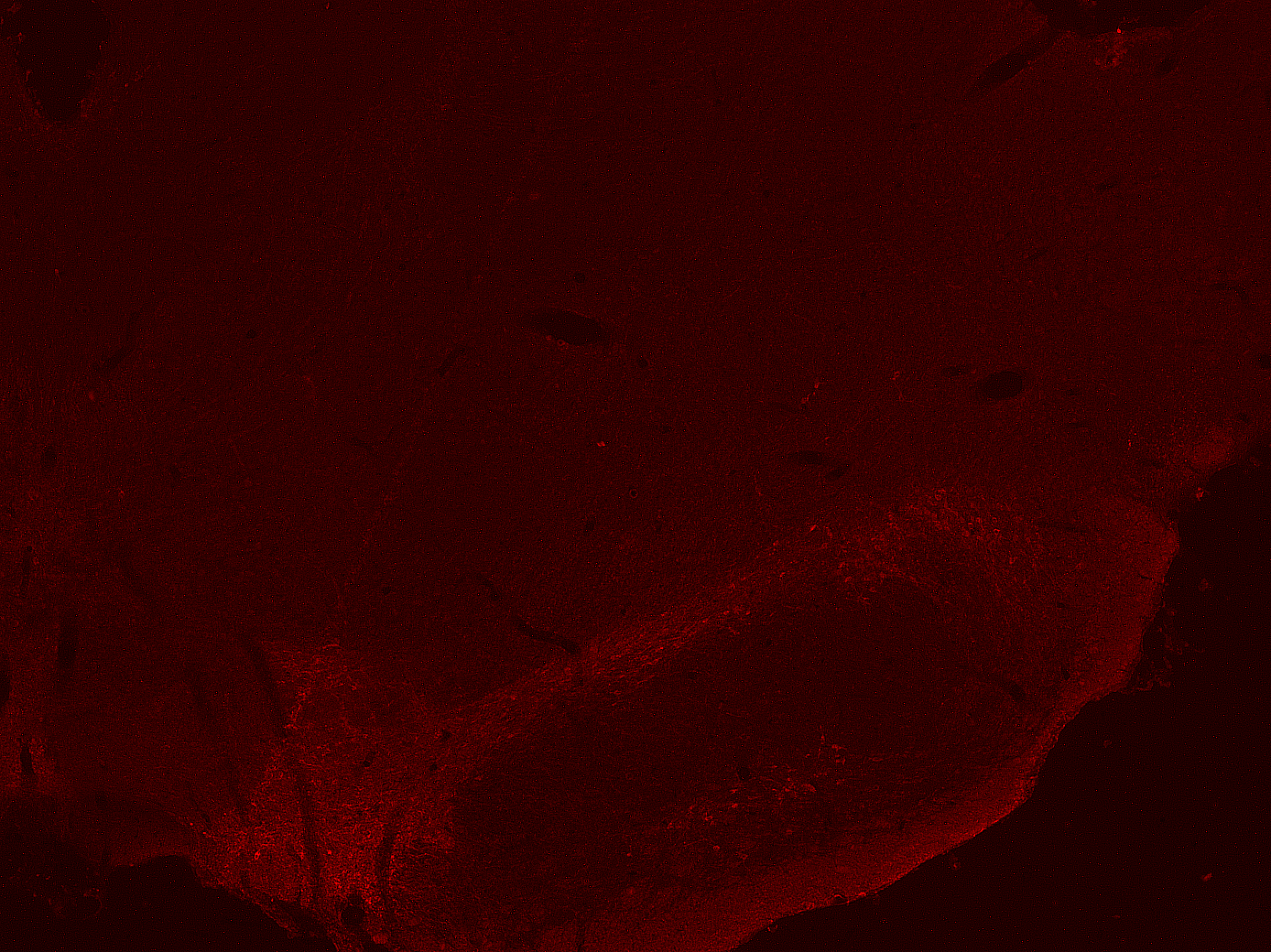

Supplement: Supplementary file 1 [file biomedicines-11-00820-s001.zip › Supplementary_Data_file_S2/TH/PNG - TH channel/CFA-LHb108-TH-568-VTA-2021-0034-Right.zvi - C=1.png]

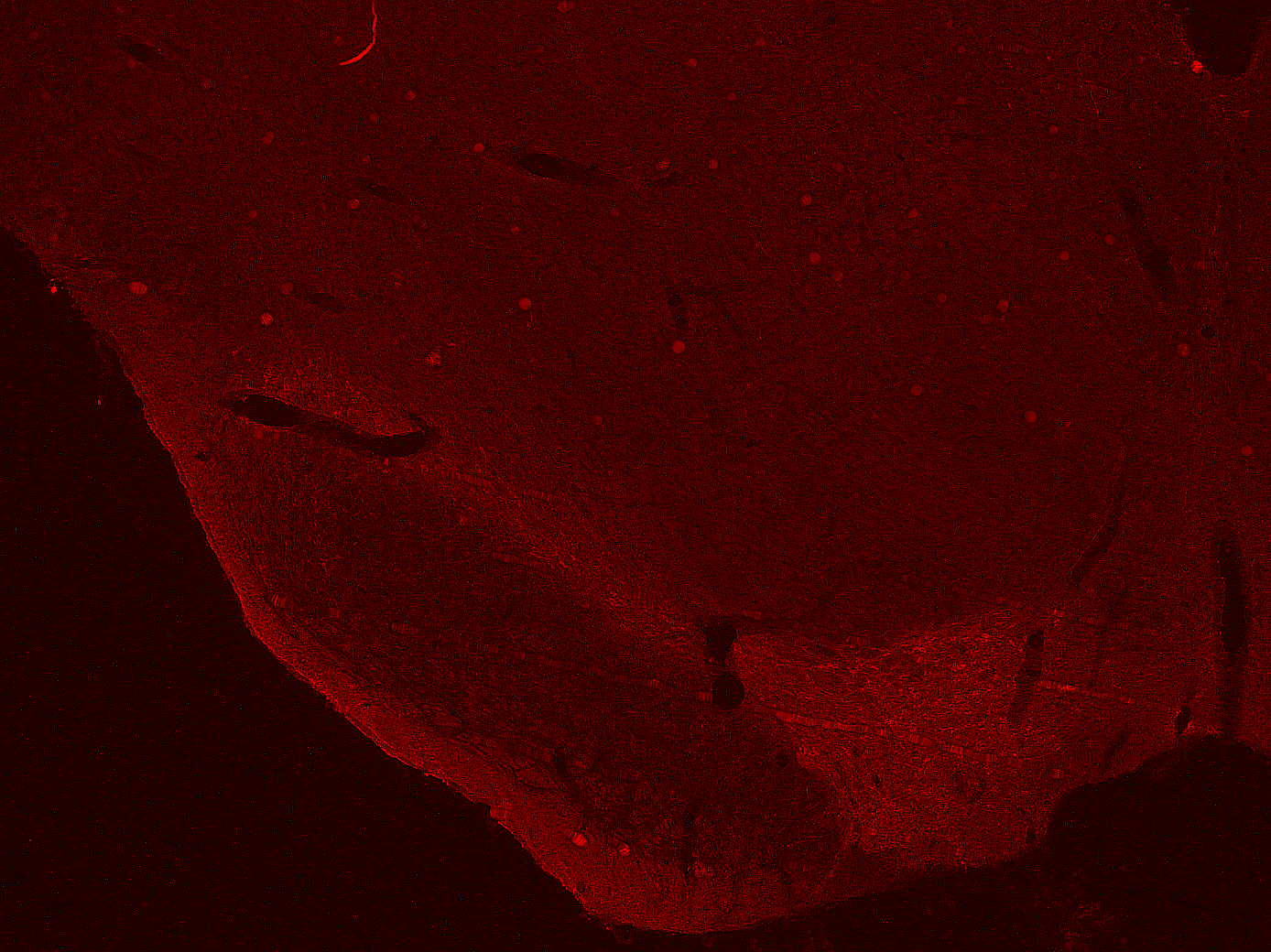

Supplement: Supplementary file 1 [file biomedicines-11-00820-s001.zip › Supplementary_Data_file_S2/TH/PNG - TH channel/CFA-LHb109-TH-568-VTA-2021-0035-Left.zvi - C=1.png]

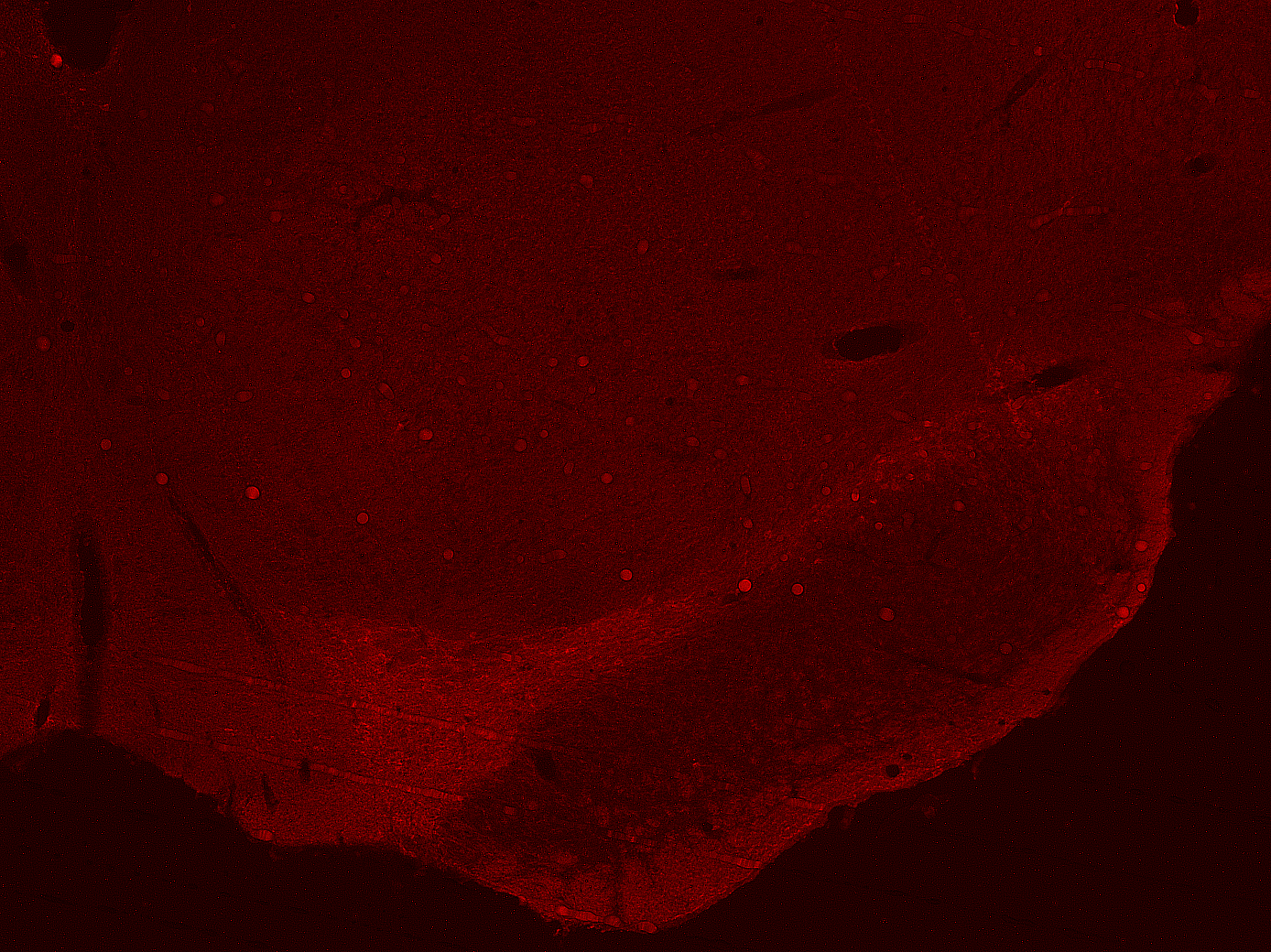

Supplement: Supplementary file 1 [file biomedicines-11-00820-s001.zip › Supplementary_Data_file_S2/TH/PNG - TH channel/CFA-LHb109-TH-568-VTA-2021-0036-Right.zvi - C=1.png]

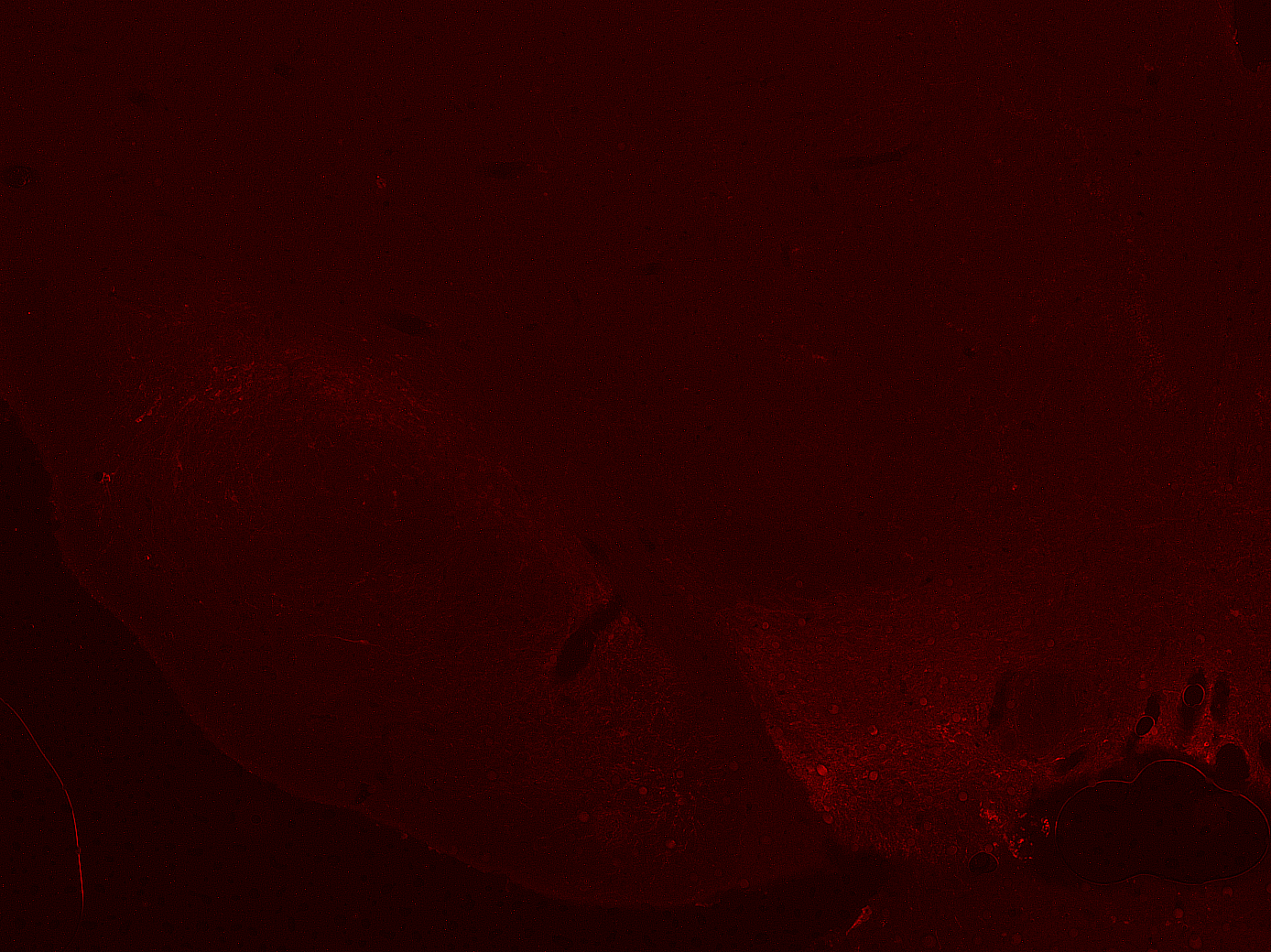

Supplement: Supplementary file 1 [file biomedicines-11-00820-s001.zip › Supplementary_Data_file_S2/TH/PNG - TH channel/SHAM-LHb100-TH-568-VTA-2021-0010-Left.zvi - C=1.png]

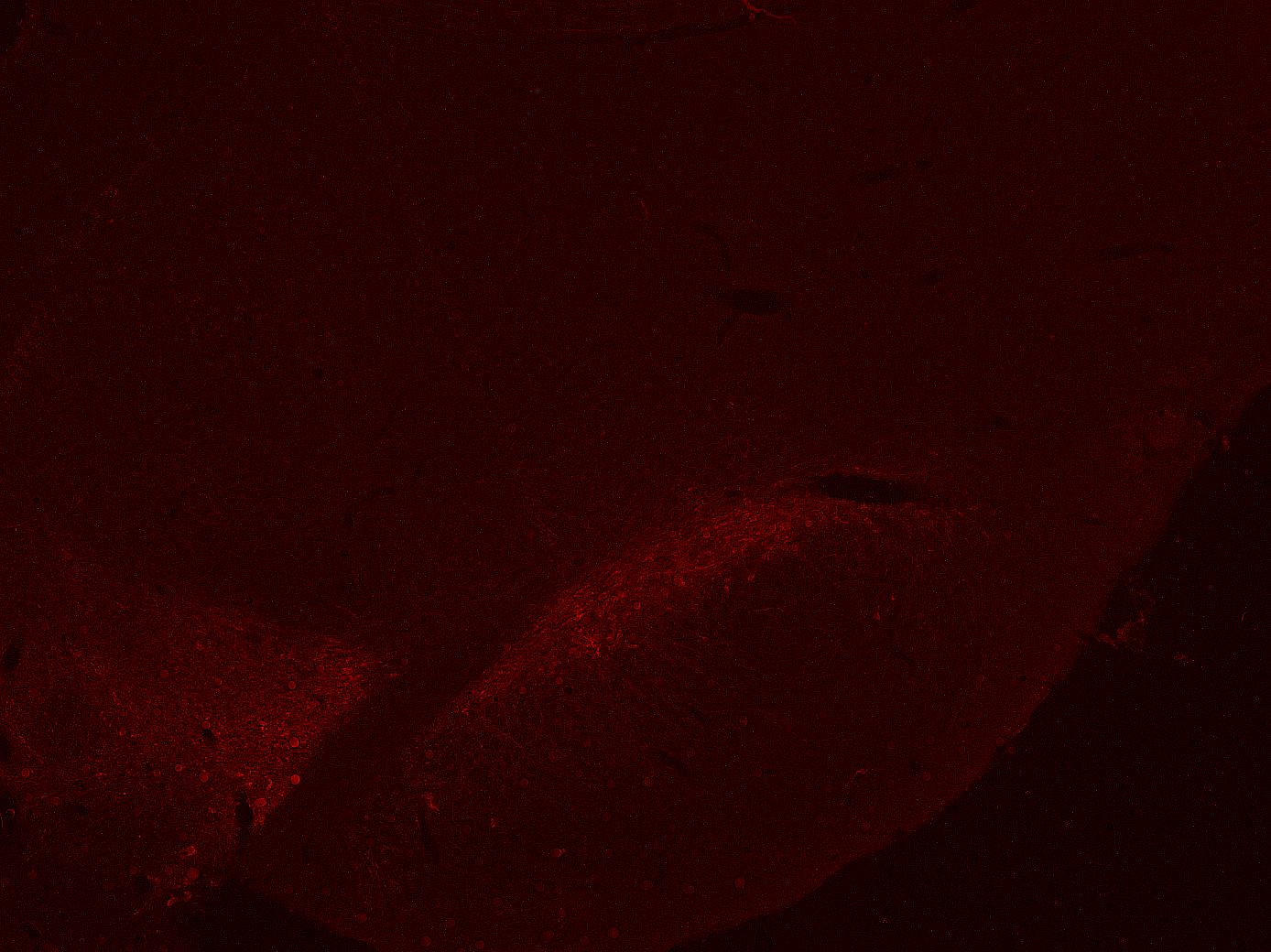

Supplement: Supplementary file 1 [file biomedicines-11-00820-s001.zip › Supplementary_Data_file_S2/TH/PNG - TH channel/SHAM-LHb100-TH-568-VTA-2021-0011-Right.zvi - C=1.png]

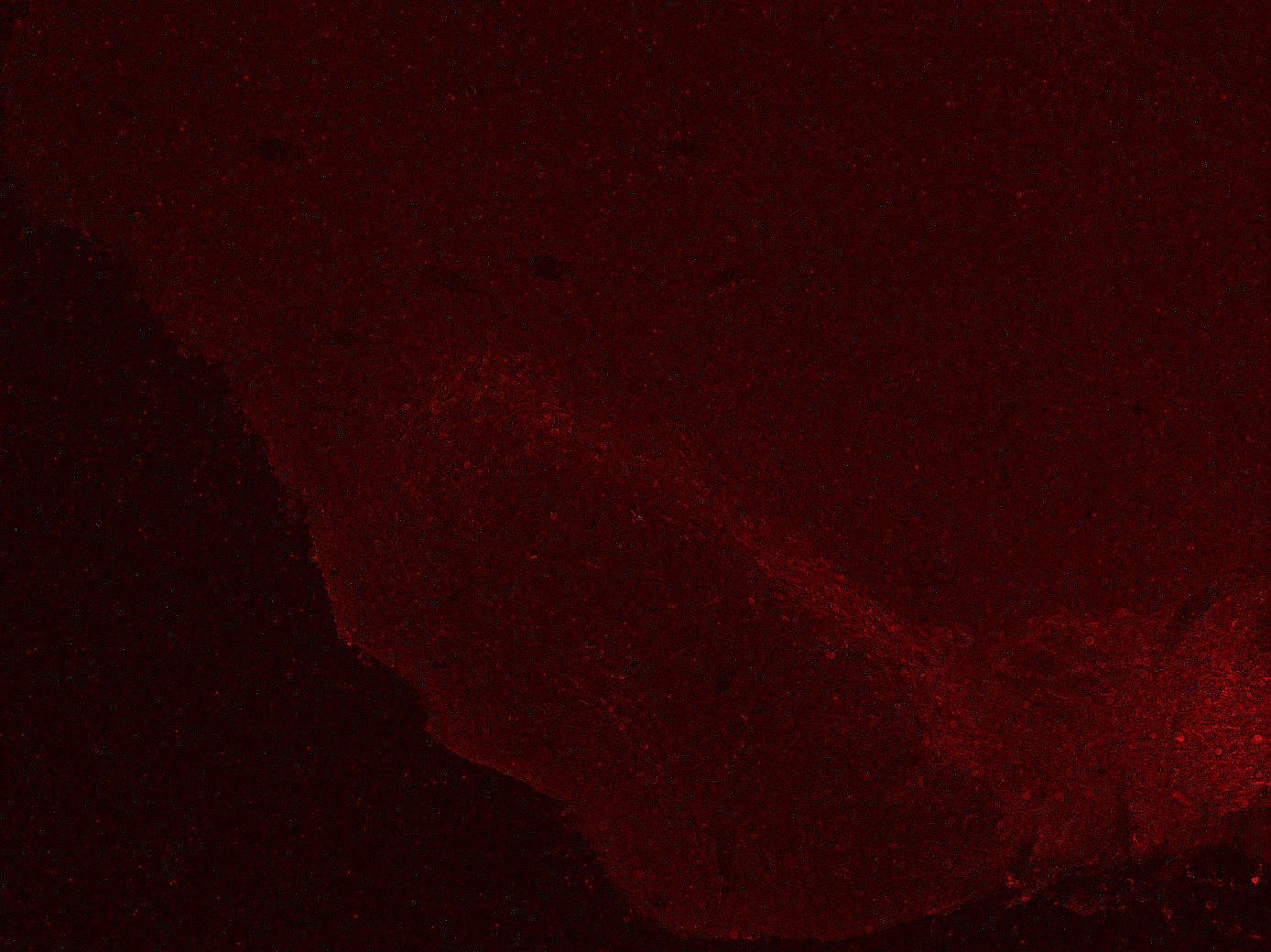

Supplement: Supplementary file 1 [file biomedicines-11-00820-s001.zip › Supplementary_Data_file_S2/TH/PNG - TH channel/SHAM-LHb101-TH-568-VTA-2021-0016-Left.zvi - C=1.png]

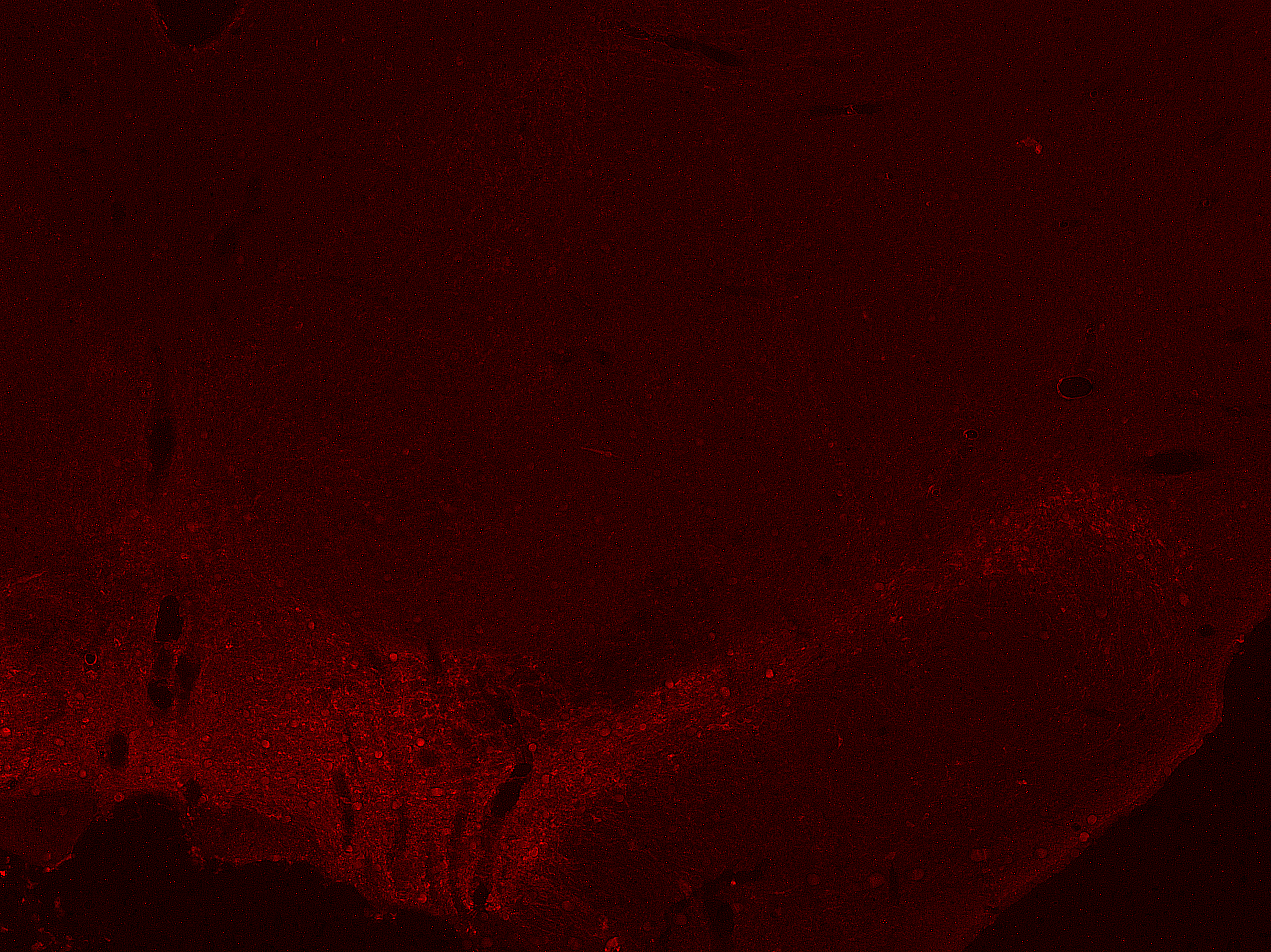

Supplement: Supplementary file 1 [file biomedicines-11-00820-s001.zip › Supplementary_Data_file_S2/TH/PNG - TH channel/SHAM-LHb101-TH-568-VTA-2021-0017-Right.zvi - C=1.png]

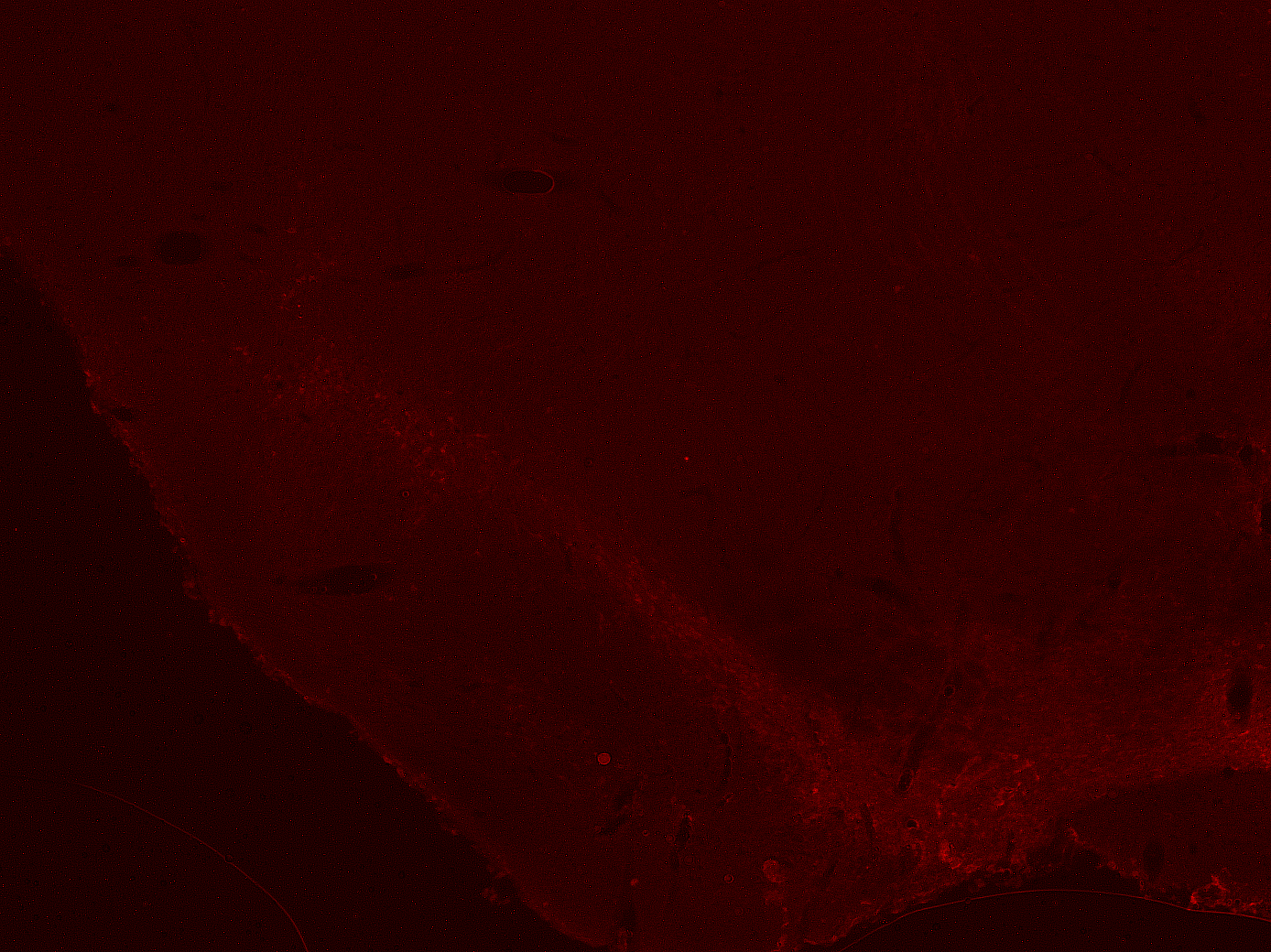

Supplement: Supplementary file 1 [file biomedicines-11-00820-s001.zip › Supplementary_Data_file_S2/TH/PNG - TH channel/SHAM-LHb103-TH-568-VTA-2021-0024-Left.zvi - C=1.png]

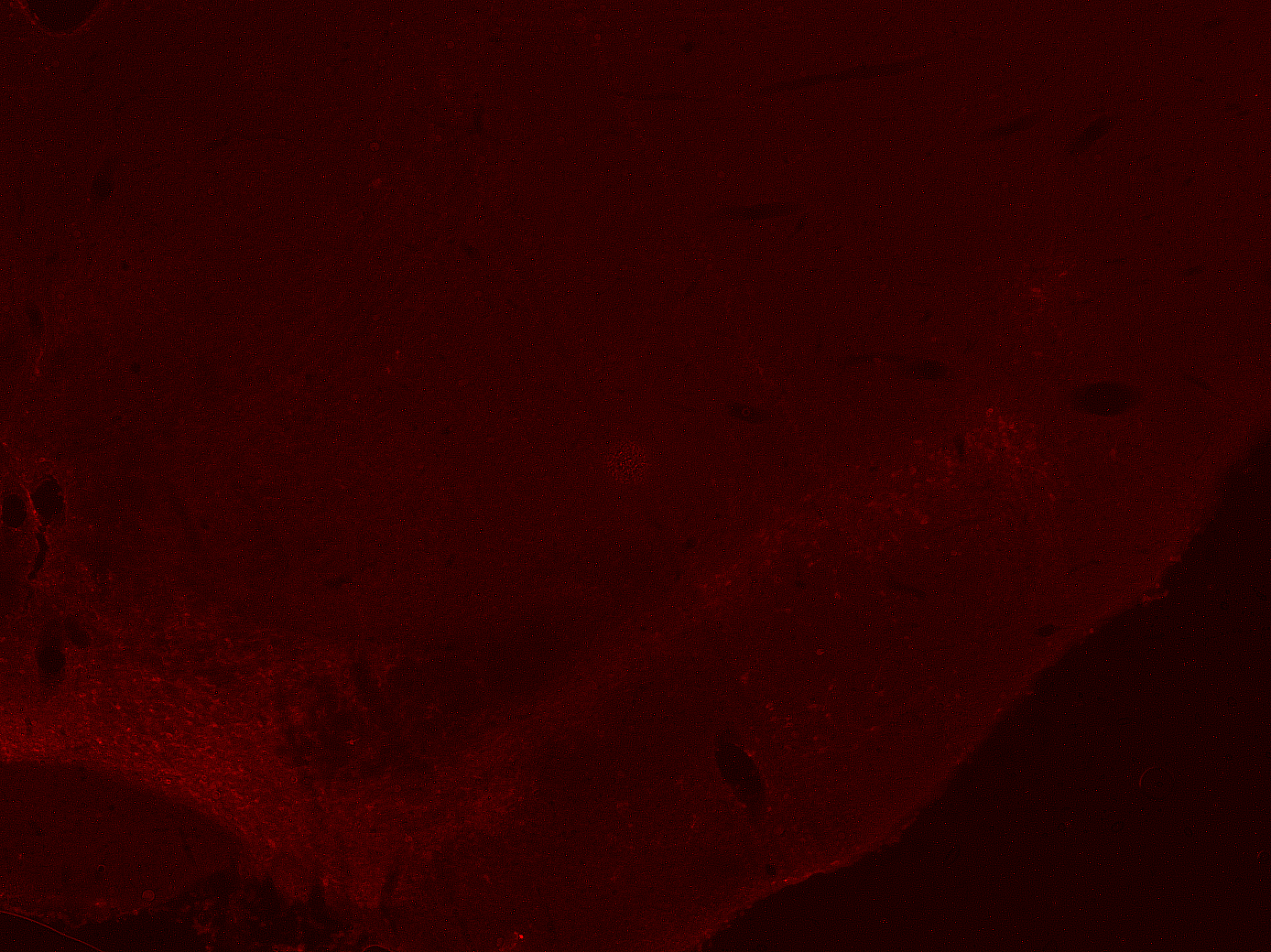

Supplement: Supplementary file 1 [file biomedicines-11-00820-s001.zip › Supplementary_Data_file_S2/TH/PNG - TH channel/SHAM-LHb103-TH-568-VTA-2021-0025-Right.zvi - C=1.png]

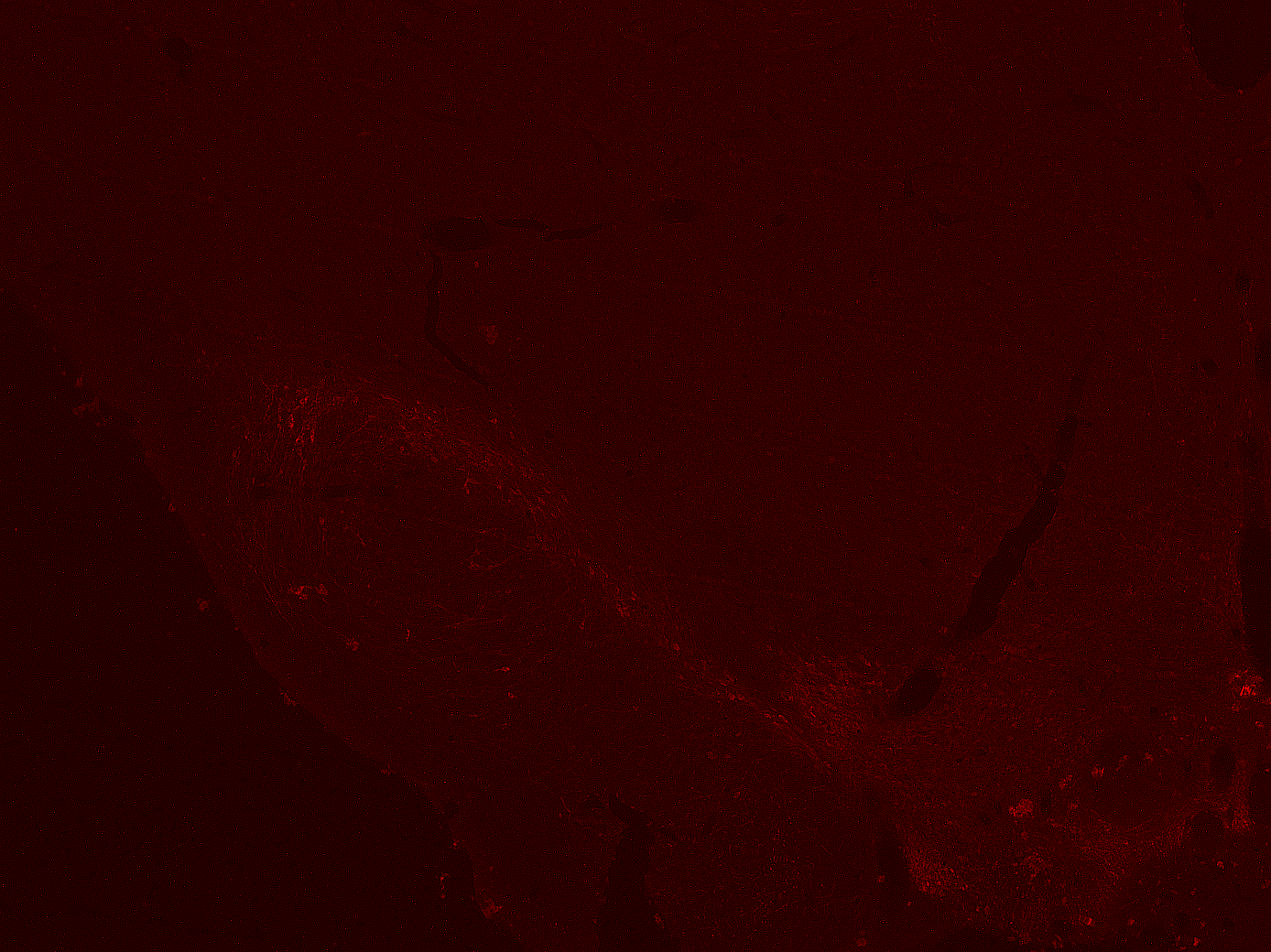

Supplement: Supplementary file 1 [file biomedicines-11-00820-s001.zip › Supplementary_Data_file_S2/TH/PNG - TH channel/SHAM-LHb110-TH-568-VTA-2021-0037-Left.zvi - C=1.png]

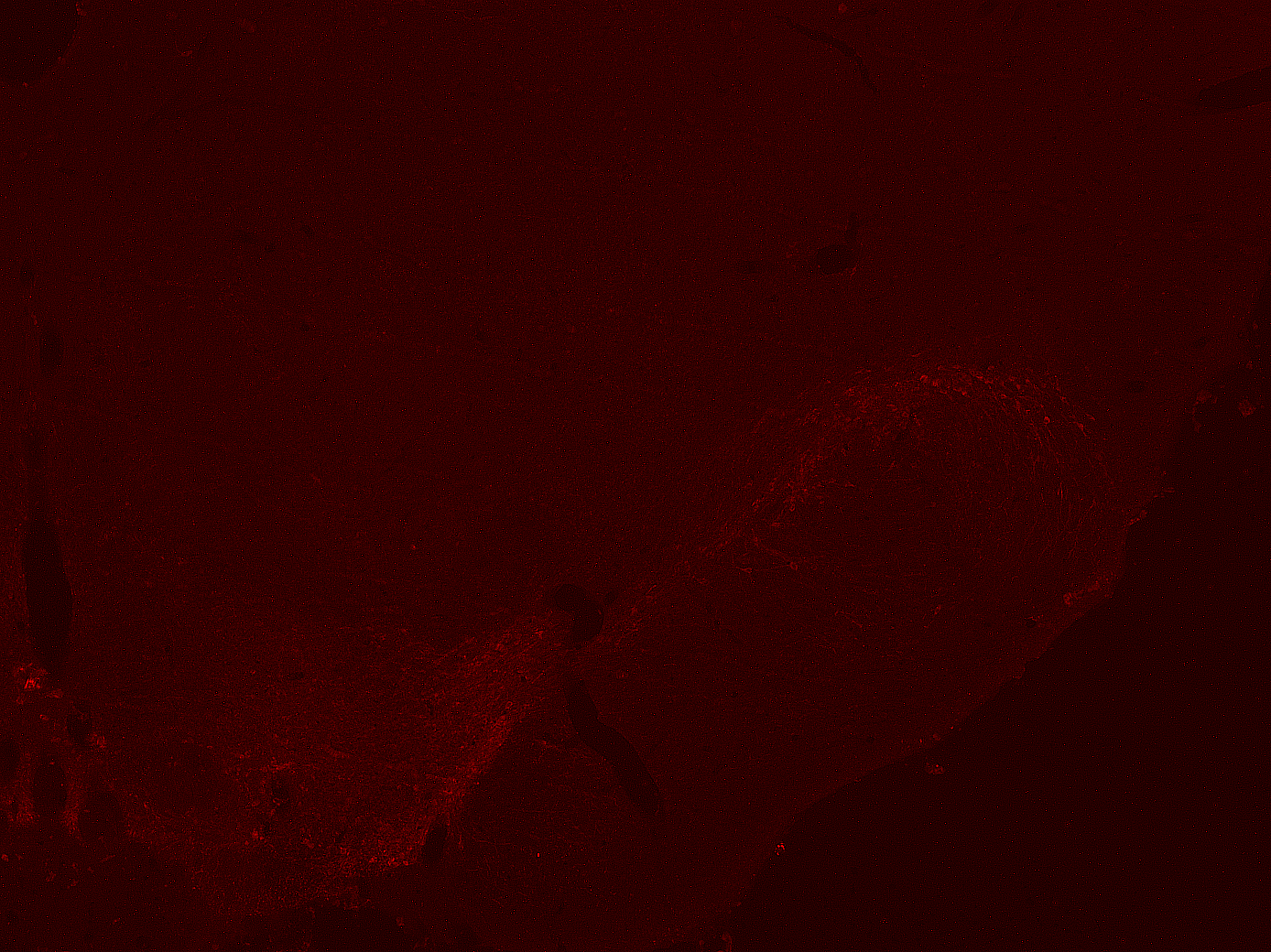

Supplement: Supplementary file 1 [file biomedicines-11-00820-s001.zip › Supplementary_Data_file_S2/TH/PNG - TH channel/SHAM-LHb110-TH-568-VTA-2021-0038-Right.zvi - C=1.png]

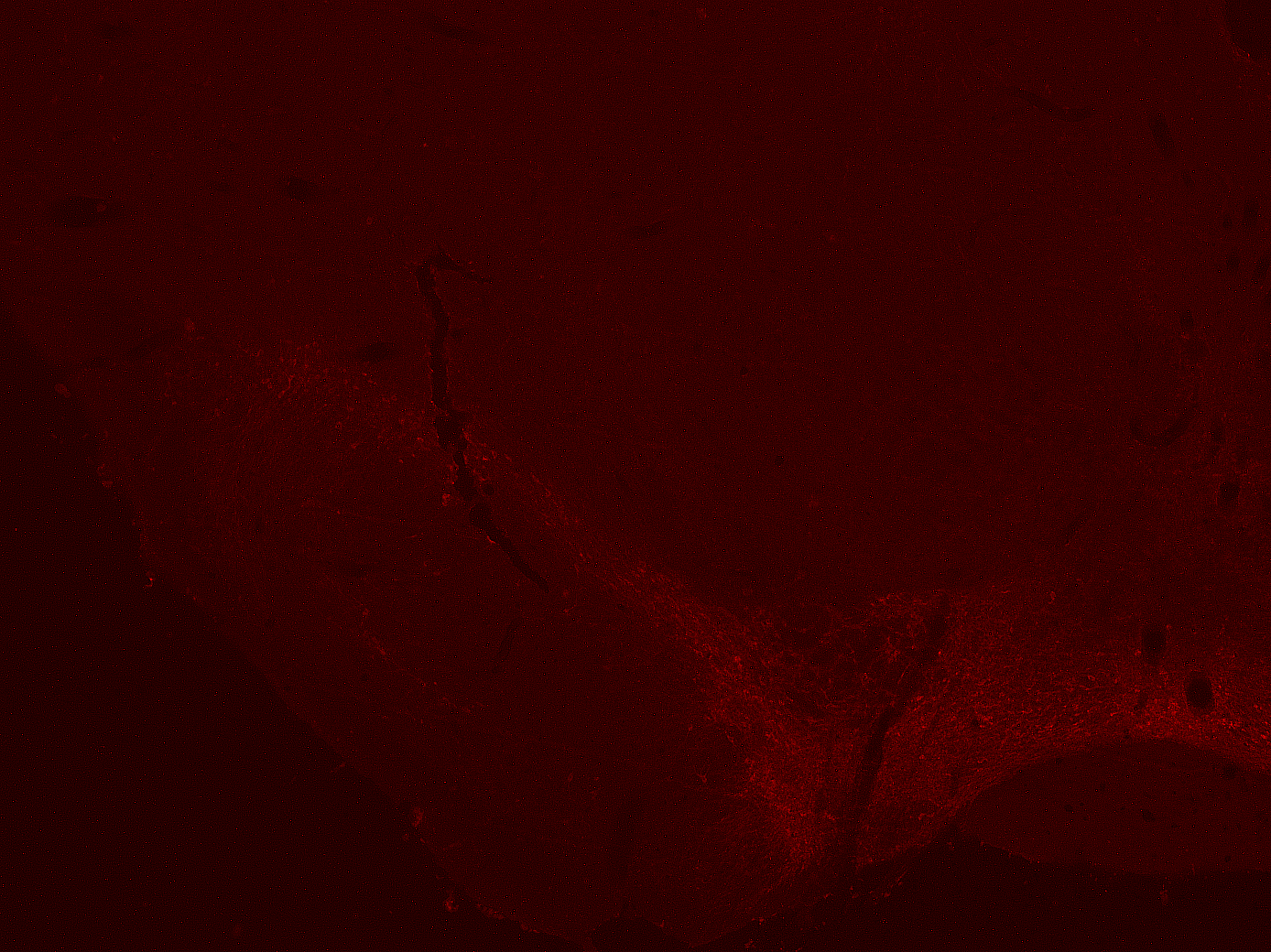

Supplement: Supplementary file 1 [file biomedicines-11-00820-s001.zip › Supplementary_Data_file_S2/TH/PNG - TH channel/SHAM-LHb111-TH-568-VTA-2021-0039-Left.zvi - C=1.png]

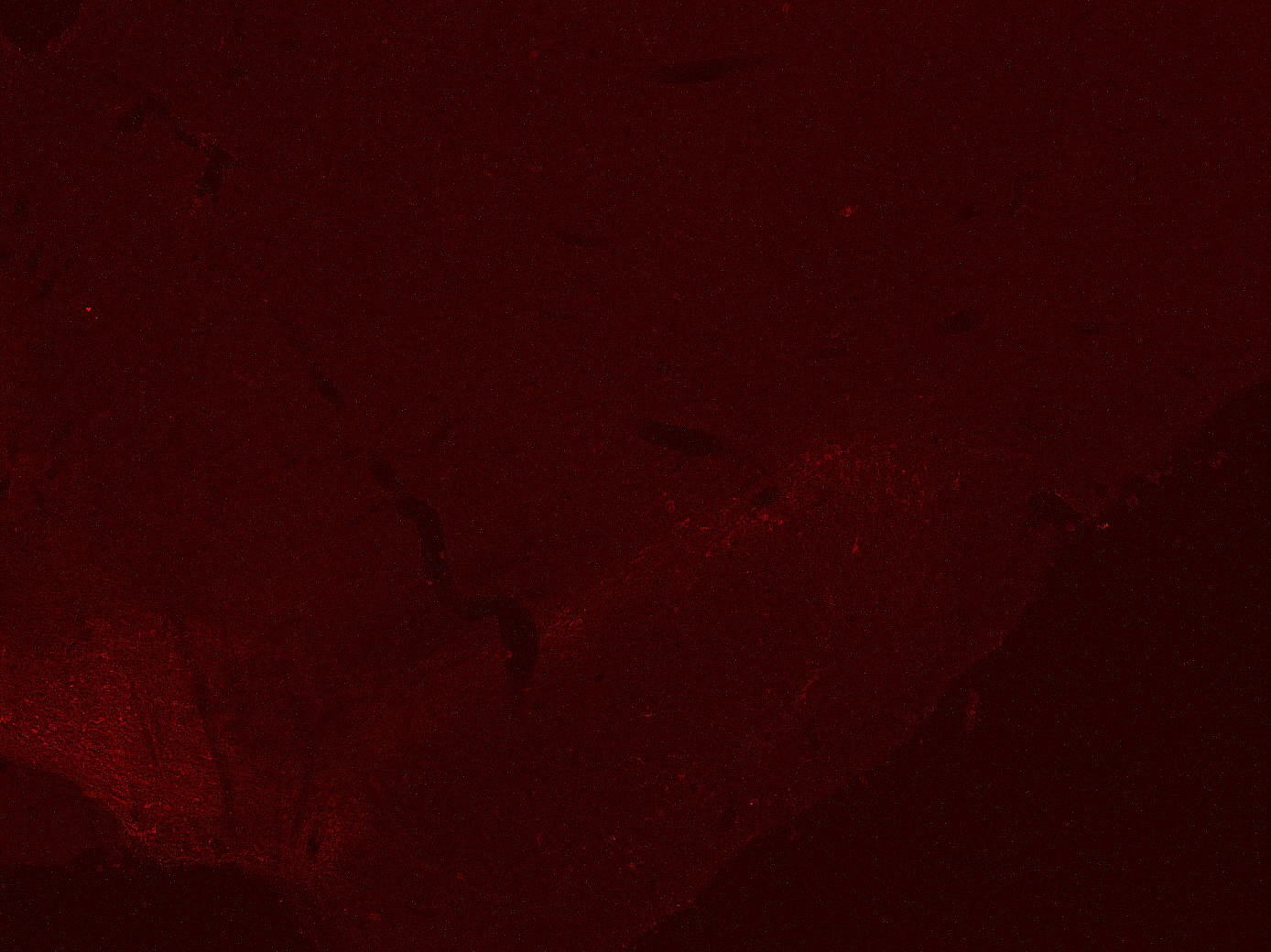

Supplement: Supplementary file 1 [file biomedicines-11-00820-s001.zip › Supplementary_Data_file_S2/TH/PNG - TH channel/SHAM-LHb111-TH-568-VTA-2021-0040-Right.zvi - C=1.png]
